# Shared Decision-Making in Cardiovascular Risk Factor Management: A Systematic Review and Meta-Analysis

**DOI:** 10.1001/jamanetworkopen.2024.3779

**Published:** 2024-03-26

**Authors:** Sabrina Elias, Yuling Chen, Xiaoyue Liu, Sarah Slone, Ruth-Alma Turkson-Ocran, Bunmi Ogungbe, Sabena Thomas, Samuel Byiringiro, Binu Koirala, Reiko Asano, Diana-Lyn Baptiste, Nicole L. Mollenkopf, Nwakaego Nmezi, Yvonne Commodore-Mensah, Cheryl R. Dennison Himmelfarb

**Affiliations:** 1 Johns Hopkins School of Nursing, Baltimore, Maryland; 2 New York University Rory Meyers College of Nursing, New York, New York; 3 Division of General Medicine, Beth Israel Deaconess Medical Center, Harvard Medical School, Boston, Massachusetts; 4 Adelphi University, Garden City, New York; 5 Catholic University of America, Washington, DC; 6 MedStar National Rehabilitation Hospital, Washington, DC; 7 Johns Hopkins Bloomberg School of Public Health, Baltimore, Maryland; 8 Johns Hopkins School of Medicine, Baltimore, Maryland

## Abstract

**Importance:**

The effect of shared decision-making (SDM) and the extent of its use in interventions to improve cardiovascular risk remain unclear.

**Objective:**

To assess the extent to which SDM is used in interventions aimed to enhance the management of cardiovascular risk factors and to explore the association of SDM with decisional outcomes, cardiovascular risk factors, and health behaviors.

**Data Sources:**

For this systematic review and meta-analysis, a literature search was conducted in the Medline, CINAHL, Embase, Cochrane, Web of Science, Scopus, and ClinicalTrials.gov databases for articles published from inception to June 24, 2022, without language restrictions.

**Study Selection:**

Randomized clinical trials (RCTs) comparing SDM-based interventions with standard of care for cardiovascular risk factor management were included.

**Data Extraction and Synthesis:**

The systematic search resulted in 9365 references. Duplicates were removed, and 2 independent reviewers screened the trials (title, abstract, and full text) and extracted data. Data were pooled using a random-effects model. The review was conducted according to the Preferred Reporting Items for Systematic Reviews and Meta-Analyses (PRISMA) reporting guideline.

**Main Outcomes and Measures:**

Decisional outcomes, cardiovascular risk factor outcomes, and health behavioral outcomes.

**Results:**

This review included 57 RCTs with 88 578 patients and 1341 clinicians. A total of 59 articles were included, as 2 RCTs were reported twice. Nearly half of the studies (29 [49.2%]) tested interventions that targeted both patients and clinicians, and an equal number (29 [49.2%]) exclusively focused on patients. More than half (32 [54.2%]) focused on diabetes management, and one-quarter focused on multiple cardiovascular risk factors (14 [23.7%]). Most studies (35 [59.3%]) assessed cardiovascular risk factors and health behaviors as well as decisional outcomes. The quality of studies reviewed was low to fair. The SDM intervention was associated with a decrease of 4.21 points (95% CI, −8.21 to −0.21) in Decisional Conflict Scale scores (9 trials; *I*^2^ = 85.6%) and a decrease of 0.20% (95% CI, −0.39% to −0.01%) in hemoglobin A_1c_ (HbA_1c_) levels (18 trials; *I*^2^ = 84.2%).

**Conclusions and Relevance:**

In this systematic review and meta-analysis of the current state of research on SDM interventions for cardiovascular risk management, there was a slight reduction in decisional conflict and an improvement in HbA_1c_ levels with substantial heterogeneity. High-quality studies are needed to inform the use of SDM to improve cardiovascular risk management.

## Key Points

**Question:**

To what extent is shared decision-making (SDM) used in interventions aimed at improving cardiovascular risk factor management, and how does SDM affect decisional outcomes, cardiovascular risk factors, and health behaviors?

**Findings:**

In this systematic review and meta-analysis of 57 randomized clinical trials that included 88 578 patients on SDM interventions for cardiovascular risk management and 1341 clinicians, SDM interventions were associated with a slight decrease in decisional conflict and an improvement in hemoglobin A_1c_ levels with substantial heterogeneity.

**Meaning:**

These findings may help advance the field of SDM interventions for cardiovascular risk management.

## Teaser

This systematic review and meta-analysis examines the use and outcomes of shared decision-making in interventions to enhance management of cardiovascular risk factors.

## Introduction

Cardiovascular risk factors such as hypertension, diabetes, obesity, and current smoking are modifiable, and the prevention and management of these conditions is important in decreasing cardiovascular disease (CVD)–related morbidity and mortality.^1,2,3^ Shared decision-making (SDM) is a collaborative approach that incorporates the active involvement of patients in decisions concerning their care.^4^ This approach enables patients, in concert with their clinicians, to make informed decisions about their health care that incorporate their goals, values, and preferences.^5^

Previous systematic reviews showed that the implementation of SDM in primary care may be effective in reducing decisional conflict and improving knowledge of diseases and treatment options, awareness of risk, and satisfaction with the decisions made.^6,7,8,9^ The incorporation of SDM in cardiovascular risk prevention and management has the potential to increase patient engagement in lifestyle changes, medication adherence, and glycemic and blood pressure control and lead to improved health outcomes.^5^ Shared decision-making may promote equity by involving clinicians and patients, sharing the best available evidence, and recognizing the needs, values, and experiences of individuals and their families when faced with the task of making decisions.^5^

Although SDM is increasingly embraced in health care and recommended in cardiovascular guidelines,^1,2^ the extent to which SDM is used in research assessing interventions for managing cardiovascular risk factors remains unclear. In addition, the effect of SDM-based interventions that focus on managing cardiovascular risk factors on decisional outcomes, cardiovascular risk factors, and health behaviors remains uncertain.

To address this gap, we performed a systematic review of trials to assess the extent to which SDM is used in interventions aimed at enhancing the management of cardiovascular risk factors (diabetes, hypertension, dyslipidemia, overweight and obesity, and tobacco use) in clinical practice. To achieve this, we developed an evidence map by thoroughly exploring the extensive literature to identify existing interventions (for patients and clinicians) and outcomes (including decisional outcomes, cardiovascular risk factor outcomes, and health behavioral outcomes) studied in trials that used SDM interventions to manage cardiovascular risk factors. Furthermore, we performed meta-analyses to evaluate the association of SDM interventions with the main outcomes of interest, including decisional conflict, hemoglobin A_1c_ (HbA_1c_), and systolic blood pressure (SBP) levels.

## Methods

### Protocol and Guidance

The protocol for this systematic review and meta-analysis was based on the Preferred Reporting Items for Systematic Reviews and Meta-Analyses (PRISMA) reporting guideline and the *Cochrane Handbook for Systematic Reviews of Interventions*.^10^ The protocol was registered with PROSPERO (CRD42021261433).

### Information Sources and Search Strategy

A public health informationist supported the team in the development of the search strategy. The Medline (via PubMed), CINAHL (Cumulative Index of Nursing and Allied Health Literature), Embase, Cochrane, Web of Science, Scopus, and ClinicalTrials.gov databases were searched for all primary studies published through June 24, 2022, without language restrictions. The search strategy included MeSH (Medical Subject Headings) terms, CINAHL headings, and Emtree terms. In addition, we used various combinations of search terms related to the concepts of *shared decision-making*, *decision-making*, *patient participation*, *communication*, and *professional-patient relations* and terms related to cardiovascular health. The full search strategy is presented in eMethods 1 in Supplement 1.

### Eligibility Criteria

Studies were included in this review if they met the following criteria: (1) the population included adults (aged ≥18 years) with cardiovascular risk factors (diabetes, hypertension, dyslipidemia, overweight and obesity, and tobacco use); (2) an SDM-based intervention type was used; (3) outcomes examined included decisional outcomes (decisional conflict, decisional quality, or SDM scores), cardiovascular risk factor outcomes (HbA_1c_, SBP levels, low-density lipoprotein, total cholesterol, smoking cessation, or CVD risk), and health behavioral outcomes (physical activity, healthy diet, or medication management); and (4) a randomized clinical trial (RCT) study design was used. In our systematic review, we identified interventions as SDM based if they met the following criteria based on the published scientific statement^5^: clinicians and patients made decisions together, the best available evidence was used, and decisions were based on patients’ informed values or preferences.

We excluded letters, editorials, and protocols. We also excluded nonrandomized trials, interrupted time-series studies, controlled before-and-after studies, prospective and retrospective comparative cohort studies, case-control or nested case-control studies, cross-sectional studies, reviews, case series, and case reports.

### Study Selection and Data Collection Process

All studies identified via database searches were imported into Covidence, a web-based collaboration software platform, for screening and data extraction.^11^ For the screening, data extraction, and quality appraisal, each article was assigned to 2 investigators from the team (S.E., Y.C., X.L., S.S., R.-A.T.-O., B.O., S.T., S.B., B.K., R.A., D.-L.B., N.L.M., and N.N.) and to a third author also from the team who was not one of the first 2 authors who initially evaluated studies. Titles and abstracts, and later full-text articles, were independently screened for relevance by 2 investigators, and discrepancies in study selection were discussed via consensus in Covidence by a third author. All articles from primary studies that met the eligibility criteria were included (1) to fully appraise the evolution of SDM in research evaluating interventions for managing cardiovascular risk factors and (2) to evaluate the association of SDM with the decision-making process and the management of cardiovascular risk factors. Full-text articles were obtained for all citations that met the inclusion criteria during the screening phase.

Two independent investigators extracted data from the studies included in the systematic review using a standardized data extraction form that was incorporated into Covidence. Disagreements were resolved via consensus in Covidence and by a third author.^11^

### Quality Appraisal

We used the Quality Assessment Tool for Controlled Intervention Studies Criteria (eMethods 2 and eTable 1 in Supplement 1) developed by the National Heart, Lung, and Blood Institute.^12^ This tool addresses 14 elements of quality and risk assessment, which provides an overall rating of good, fair, or poor based on critical appraisal of characteristics relevant to high-quality research studies.^13^ Two researchers independently assessed each article, and a third researcher evaluated any discrepancies in the cross-check for consensus.

### Data Synthesis

Meta-analyses were conducted to assess the pooled effect size of SDM interventions on decisional conflict, HbA_1c_ (percentages), and SBP levels (in millimeters of mercury). We chose these 3 outcomes for meta-analysis due to the consistent measurement methods or tools used across studies, and because they represent the main outcomes commonly reported in trials. Decisional conflict is defined as the individual’s personal uncertainty regarding the choice of options,^14^ which is one of the frequently reported outcomes in studies examining SDM-based interventions. Decisional conflict was evaluated using the Decisional Conflict Scale, a validated measure assessing patient perception of uncertainty in choosing between options, with summary scores ranging from 0 (no decisional conflict) to 100 (extremely high decisional conflict).^14^

### Statistical Analysis

We used means (SDs) of the outcomes measured at the follow-up time points to perform meta-analyses. The effect size is presented as the mean difference between the experimental intervention group and the control group accompanied by the 95% CI (data transformation method presented in eMethods 3 in Supplement 1). Heterogeneity was assessed using the Cochran *Q* test and the Higgins *I*^2^ statistic.^15^ The interpretation of *I*^2^ values followed Cochrane guidelines.^10^ The Hedges test was used to evaluate publication bias, and funnel plots were examined visually. Subgroup meta-analyses were carried out for different study durations as reported across the studies. All meta-analyses were conducted using Stata/SE, version 17.0 (StataCorp LLC).

## Results

### Search Results

**Figure 1.**
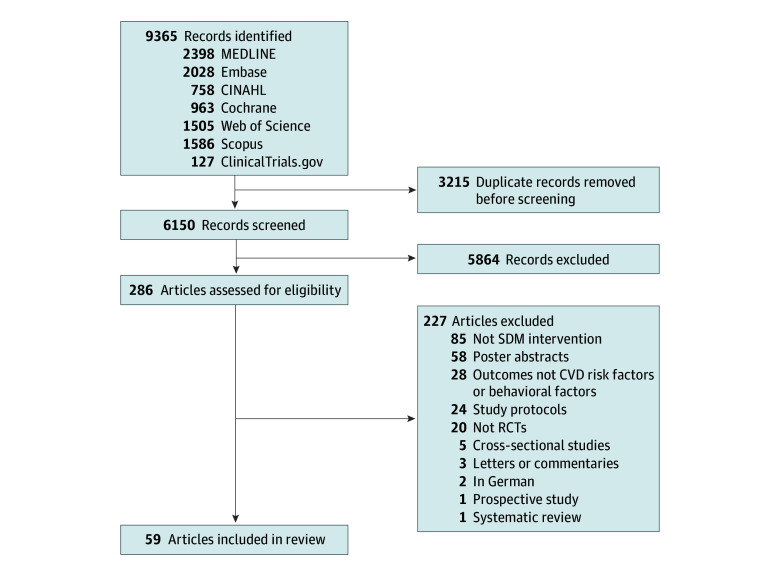
Study Flow Diagram CINAHL indicates Cumulative Index of Nursing and Allied Health Literature; CVD, cardiovascular disease; SDM, shared decision-making; and RCT, randomized clinical trial.

Our systematic search resulted in 9365 references, 3215 of which were duplicates. After the titles and abstracts of the identified references were read, 5863 articles were excluded for not fulfilling the inclusion criteria. Full-text screening was performed on the remaining 286 articles, and 227 were excluded (Figure 1). Thus, 57 RCTs with 88 578 patients and 1341 clinicians were included in this review.^16,17,18,19,20,21,22,23,24,25,26,27,28,29,30,31,32,33,34,35,36,37,38,39,40,41,42,43,44,45,46,47,48,49,50,51,52,53,54,55,56,57,58,59,60,61,62,63,64,65,66,67,68,69,70,71,72,73,74^ A total of 59 articles were included, as 2 RCTs were reported twice.^44,45,70,71^

### Study and Participant Characteristics

The 59 included articles were published between 1988^54^ and 2022^34,42^ (Table 1 and eTable 2 in Supplement 1). Of these studies, 26 (44.1%) were conducted in the US,^20,21,27,28,29,31,34,35,36,37,41,42,46,49,52,53,54,55,58,59,64,68,69,70,71,73^ 9 (15.3%) in Germany,^17,19,25,26,43,60,66,67,72^ 6 (10.2%) in the Netherlands,^23,44,45,50,61,63^ 4 (6.8%) in the United Kingdom,^16,38,56,57^ 3 (5.1%) in China,^22,51,65^ 3 (5.1%) in Spain,^30,32,62^ and the remaining 8 (13.6%) in Australia,^33^ Canada,^18^ Denmark,^39^ Estonia,^47^ Finland,^24^ Greece,^48^ India,^74^ or South Korea.^40^ Sample sizes ranged from 40 participants^52^ to 10 815 participants^60^ across the included studies. The mean age of patients in the included studies ranged from 24.5 years^56^ to 78.9^62^ years. Among the 59 articles, 20 also reported health care clinician characteristics,^18,19,20,23,28,29,30,32,34,36,42,43,44,45,55,59,62,64,69,72^ with sample sizes ranging from 11 participants^34^ to 230 participants.^55^ The mean age of clinicians ranged from 38 years^45^ to 49 years.^23^

**Table 1.** Summary of Study Characteristics

Study	Location	Target and main topic	Purpose and population	Design and setting	Sample size	
					No. of patients	No. of clinicians
Adarkwah et al,^72^ 2016	Germany	Patients and clinicians; multiple CVD risk factors	To compare the new TTE illustration with the established emoticons looking at the degree of SDM in the consultation process and various secondary outcomes of decisional conflict and accessibility	Cluster RCT: practice, clinic, or office	304	32
Applegate et al,^73^ 2021	US	Patients only; multiple CVD risk factors	To determine whether Project ACTIVE improved utilization of preventive care, estimated life expectancy compared with usual care, or both	RCT: practice, clinic, or office; hospital	132	NR
Bailey et al,^71^ 2016	US	Patients only; diabetes	To test the effectiveness of the intervention in clinical practice and assess whether an EHR-linked clinical decision support system slowed increases in modifiable cardiovascular risk among adults with serious mental illness	Pragmatic RCT: practice, clinic, or office	225	NR
Bailey et al,^70^ 2018	US	Patients only; diabetes	To assess the effectiveness of different interventions on knowledge transfer and behavior modification to improve PROMs for T2D	Pragmatic RCT: practice, clinic, or office	225	NR
Boulware et al,^68^ 2020	US	Patients only; hypertension	To assess whether an intervention to help patients prioritize goals for their visit would improve patient-clinician communication and clinical outcomes	Pragmatic, comparative effectiveness RCT: practice, clinic, or office; home	159	NR
Branda et al,^69^ 2013	US	Patients and clinicians; diabetes	To evaluate the effect of PDAs vs usual care on decision-making measures, metabolic control, and medication adherence among patients with T2D	Cluster RCT: practice, clinic, or office; other (nonacademic and rural primary care practice)	103	41
Buhse et al,^66^ 2015	Germany	Patients and clinicians; diabetes	To investigate the cardiovascular risk factor profile among young men (aged 18-50 y) with hypertension in family practices and analyze the effectiveness of a computer-based decision aid promoting SDM in modifying cardiovascular risk factors	RCT: practice, clinic, or office	154	NR
Buhse et al,^67^ 2018	Germany	Patients and clinicians; diabetes	To determine whether communicating personalized statin therapy effects obtained by a prognostic algorithm leads to lower decisional conflict associated with statin use in patients with stable CVD compared with standard (nonpersonalized) therapy effects	Cluster RCT: practice, clinic, or office	279	NR
Cheng et al,^65^ 2021	China	Patients only; diabetes	To compare the new TTE illustration with the established emoticons looking at the degree of SDM in the consultation process and various secondary outcomes such as decisional conflict and accessibility	RCT: practice, clinic, or office; hospital	242	NR
Cooper et al,^64^ 2011	US	Patients and clinicians; hypertension	To compare the effectiveness of patient and physician interventions (separately and in combination with one another) with the effectiveness of minimal interventions, by evaluating the effect of the intervention on (1) patient and physician communication behaviors, (2) patient ratings of the interpersonal process of care, (3) patient adherence to medications, and (4) BP levels and control over 12 mo	RCT: practice, clinic, or office	279	41
Coronado-Vázquez et al,^62^ 2019	Spain	Patients and clinicians; multiple CVD risk factors and medication	To determine the effectiveness of an SDM intervention for medication appropriateness in patients with chronic diseases and polypharmacy	Randomized, multicenter quasi-experimental study: practice, clinic, or office	122	22
Den Ouden et al,^63^ 2017	Netherlands	Patients and clinicians; multiple CVD risk factors	To evaluate in a cluster-randomized practical trial the effect of PDAs vs usual care on decision-making measures, metabolic control, and medication adherence in nonacademic and rural primary care practices and their patients with T2D	Cluster RCT: practice, clinic, or office	153	NR
Denig et al,^61^ 2014	Netherlands	Patients only; diabetes	To evaluate an informed SDM program for individuals with T2D under high-fidelity conditions	Pragmatic RCT: practice, clinic, or office	344	NR
Dwinger et al,^60^ 2020	Germany	Patients only; multiple CVD risk factors	To determine the effectiveness of a PDA for patients with T2D receiving metformin who require treatment intensification	Prospective, pragmatic RCT: community	10 815	NR
Eaton et al,^59^ 2011	US	Patients and clinicians; multiple CVD risk factors	To determine whether an intervention based on patient activation and a physician decision support tool is more effective than usual care for improving adherence to National Cholesterol Education Program guidelines	Cluster RCT: practice, clinic, or office	4105	55
Eckman et al,^58^ 2012	US	Patients only; multiple CVD risk factors	To evaluate the effectiveness of an empowerment self-management intervention on psychological distress and quality of life among patients with poorly controlled T2D	RCT: ambulatory setting	170	NR
Edwards et al,^57^ 2006	UK	Patients only; diabetes	To compare the effectiveness of patient and physician interventions (separately and in combination with one another) on (1) patient and physician communication behaviors, (2) patient ratings of the interpersonal process of care, (3) patient adherence to medications, and (4) BP levels and control over 12 mo	RCT: online	508	NR
Farmer et al,^56^ 2005	UK	Patients only; diabetes	To determine whether real-time telemedicine support can improve glycemic control in T1D	RCT: practice, clinic, or office	93	NR
Grant et al,^55^ 2008	US	Patients only; diabetes	To examine whether the use of diabetes-specific personal health records improves diabetes care management by increasing patient knowledge and engagement in their own care and by facilitating patient-physician communication	RCT: practice, clinic, or office; hospital; community	244	230
Greenfield et al,^54^ 1988	US	Patients only; diabetes	To examine the effects of an intervention designed to increase the involvement of patients with diabetes in medical decision-making on blood glucose control and quality of life	RCT: practice, clinic, or office	59	NR
Heisler et al,^53^ 2014	US	Patients only; diabetes	To evaluate the effectiveness of iDecide in improving key diabetes outcomes compared to delivery by CHWs of the same evidence-based information without tailoring using print consumer booklets developed by the AHRQ	RCT: practice, clinic, or office; home; or other agreed-upon place	188	NR
Hsu et al,^52^ 2016	US	Patients only; diabetes	To test the efficacy of a cloud-based diabetes management program in helping individuals starting basal insulin achieve better glycemic control	RCT: practice, clinic, or office	40	NR
Hu et al,^51^ 2021	China	Patients only; diabetes and medication	To evaluate a combined fasting blood glucose–based dosage self-titration and decision-supported telephone coaching intervention on glycemic control and diabetes self-management skills	RCT: practice, clinic, or office; via telephone calls	869	NR
Jaspers et al,^50^ 2021	Netherlands	Patients only; CVD risk	To determine whether communicating personalized statin therapy effects obtained by a prognostic algorithm leads to lower decisional conflict associated with statin use in patients with stable CVD	RCT: practice, clinic, or office	303	NR
Jouni et al,^49^ 2017	US	Patients only; multiple CVD risk factors	To assess the effect of disclosing CHD genetic risk on LDL-C levels	RCT: practice, clinic, or office	207	NR
Karagiannis et al,^48^ 2016	Greece	Patients only; diabetes	To assess the efficacy of the Diabetes Medication Choice decision aid among patients with T2D in Greece in primary and secondary care practice	Cluster RCT: practice, clinic, or office	204	NR
Kask-Flight et al,^47^ 2021	Estonia	Patients and clinicians; multiple CVD risk factors	To analyze the effectiveness of a computer-based decision aid promoting SDM in changing cardiovascular risk factors among young men (aged 18-50 y) with hypertension in family practices	Cluster RCT: practice, clinic, or office	130	NR
Keyserling et al,^46^ 2014	US	Patients only; smoking	To assess the effectiveness, acceptability, and cost-effectiveness of a combined lifestyle and medication intervention to reduce CHD risk offered in counselor-delivered and web-based formats	RCT: practice, clinic, or office	385	NR
Koelewijn-van Loon et al,^44^ 2009	Netherlands	Patients and clinicians; multiple CVD risk factors	To investigate whether a nurse-led intervention in primary care had a positive effect on lifestyle and 10-y cardiovascular risk	Cluster RCT: practice, clinic, or office	615	24
Koelewijn-van Loon et al,^45^ 2010	Netherlands	Patients and clinicians; multiple CVD risk factors	To determine whether lifestyle and risk perception improved with an intervention to involve patients in cardiovascular risk management by the practice nurse	Cluster RCT: practice, clinic, or office	615	24
Krones et al,^43^ 2008	Germany	Patients and clinicians; CVD risk	To determine the effect of promoting the effective communication of absolute CVD risk and SDM through dissemination of a simple decision aid for use in family practice consultations	Pragmatic, cluster RCT: practice, clinic, or office	1132	91
Kulzer et al,^17^ 2018	Germany	Patients and clinicians; diabetes and medication	To investigate whether taking care of patients with insulin-treated T2D using integrated personalized diabetes management improves glycemic control, PROs, and physician treatment satisfaction and intensifies therapy adjustments	Cluster RCT: practice, clinic, or office	907	NR
Kunneman et al,^42^ 2022	US	Patients and clinicians; diabetes and medication	To determine the effectiveness of an SDM tool vs guideline-informed usual care in translating evidence into primary care, and to explore how the tool changed patient perspectives about diabetes medication decision-making	Mixed methods cluster RCT: practice, clinic, or office	350	99
Lauffenburger et al,^41^ 2019	US	Patients only; diabetes and medication	To evaluate the effect of a telephone-based patient-centered intervention on glycated HbA_1c_ control for individuals with poorly controlled diabetes	Pragmatic RCT: insurance community	1400	NR
Lee et al,^40^ 2016	South Korea	Patients only; smoking	To develop a culturally tailored decision aid for smoking cessation and evaluate its effect on the use of smoking cessation medication	Pragmatic cluster RCT: practice, clinic, or office	414	NR
Maindal et al,^39^ 2014	Denmark	Patients and clinicians; diabetes	To assess whether a 12-wk participant-driven health education program offered to individuals with screening-detected hyperglycemia in Danish primary care would lead to improvements in cardiovascular risk factors, health behavior, and PROs after 3 y	RCT: practice, clinic, or office	509	NR
Mathers et al,^38^ 2012	UK	Patients and clinicians; diabetes	To determine the effectiveness of a PDA on improving decision quality and glycemic control in individuals with diabetes making treatment decisions	Pragmatic cluster RCT: practice, clinic, or office	175	NR
Moin et al,^37^ 2019	US	Patients only; diabetes and medication	To test the effectiveness of a prediabetes SDM intervention	Cluster RCT: practice, clinic, or office	1379	NR
Montgomery et al,^16^ 2003	UK	Patients only; hypertension and medication	To evaluate 2 interventions for assisting patients with newly diagnosed hypertension in the decision whether to start drug therapy for reducing BP	Factorial RCT: practice, clinic, or office; community	217	NR
Mullan et al,^36^ 2009	US	Patients only; diabetes and medication	To determine the ability of a decision aid to promote patient involvement in choosing antihyperglycemic agents and to evaluate the effects of this strategy on medication adherence and patient outcomes	Cluster RCT: practice, clinic, or office	85	40
Naik et al,^35^ 2011	US	Patients only; diabetes	To evaluate the comparative effectiveness of 2 diabetes group clinic interventions on glycated HbA_1c_ levels in primary care	RCT: practice, clinic, or office	87	NR
O’Malley et al,^34^ 2022	US	Patients and clinicians; multiple CVD risk factors and medication	To assess whether an intervention to help patients prioritize goals for their visit would improve patient-clinician communication and clinical outcomes	RCT: practice, clinic, or office	120	11
Peiris et al,^33^ 2015	Australia	Patients and clinicians; CVD risk	To test whether a multifaceted quality improvement intervention comprising computerized decision support, audit/feedback tools, and staff training improved (1) guideline-indicated risk factor measurements and (2) guideline-indicated medications for those at high CVD risk	Cluster RCT: practice, clinic, or office	38 725	NR
Perestelo-Pérez et al,^32^ 2016	Spain	Patients and clinicians; CVD risk and medication	To test whether a multifaceted quality improvement intervention comprising computerized decision support, audit/feedback tools, and staff training improved (1) guideline-indicated risk factor measurements and (2) guideline-indicated medications for those at high CVD risk	Cluster RCT: practice, clinic, or office	168	29
Prabhakaran et al,^74^ 2019	India	Patients and clinicians; multiple CVD risk factors	To evaluate the effectiveness of a nurse-facilitated, mHealth-based EDS for the integrated management of 5 chronic conditions in primary care settings in India as part of the mWellcare trial	Pragmatic RCT: community health centers	3324	NR
Ramallo-Fariña et al,^30^ 2021	Spain	Patients and clinicians; diabetes	To assess the effectiveness of different interventions of knowledge transfer and behavior modification to improve PROMs in patients with T2D	Open, community-based pragmatic, multicenter, controlled trial with random allocation by cluster: practice, clinic, or office	2334	211
Rost et al,^31^ 1991	US	Patients only; diabetes	To determine whether a short intervention that enhanced patient information seeking and decision-making during hospitalization improved metabolic control and functional status in patients with diabetes	RCT: clinical research center	61	NR
Smith et al,^29^ 2008	US	Patients and clinicians; diabetes	To assess the effect of a specialist telemedicine intervention for improving diabetes care using the CCM	RCT: primary care clinic	639	97
Sperl-Hillen et al,^28^ 2018	US	Patients and clinicians; cardiovascular risk	To evaluate whether a clinical decision support intervention can improve 10-y CVD risk trajectory in patients in primary care settings	RCT: primary care clinics	7914	102
Swoboda et al,^27^ 2017	US	Patients; diabetes	To evaluate a telephone-based goal setting and decision support coaching intervention among adults with T2D and to evaluate the effect of these approaches for diet and physical activity behavior changes in relation to an attention control group	RCT: community setting	54	NR
Tinsel et al,^26^ 2013	Germany	Clinicians; hypertension	To implement an evaluated SDM training program for GPs within the context of hypertension treatment, and to examine whether the SDM training enhanced patients’ perceived participation and lowered BP	Cluster RCT: general practices	1120	NR
Tinsel et al,^25^ 2018	Germany	Patients; CVD risk	To investigate the applicability of the DECADE intervention and the potential effects of the intervention on patients with cardiovascular risk factors	RCT: primary care	78	NR
Tusa et al,^24^ 2021	Finland	Patients; CVD risk	To examine the influence of a participatory patient care plan on health-related quality of life and disease-specific outcomes related to diabetes, ischemic heart disease, and hypertension	RCT: primary care	605	NR
Tutino et al,^22^ 2017	China	Patients; diabetes	To test whether the delivery of integrated care augmented by a web-based disease management program and nurse coordinator could improve treatment target attainment and health-related behavior	RCT: hospital	3586	NR
van Steenkiste et al,^23^ 2007	Netherlands	Clinicians and patients; multiple CVD risk factors	To test whether a decision support tool can improve primary prevention of CVD in primary care	Cluster RCT: hospital	490	34
Warner et al,^21^ 2015	US	Clinicians and patients; smoking	To develop and pilot test a decision aid to increase patient involvement in decisions regarding smoking behavior of cigarette smokers scheduled for elective surgery	RCT: academic medical center	130	NR
Weymiller et al,^20^ 2007	US	Clinicians and patients; diabetes	To examine a decision aid tool’s acceptability to patients and its effect on patient knowledge of the information about the potential merits and demerits of the options and decisional conflict	Cluster RCT: metabolic clinic	98	21
Wollny et al,^19^ 2019	Germany	Clinicians and patients; diabetes	To investigate whether an educational intervention facilitated by GPs increased patient-centeredness and perceived SDM in the treatment of patients with poorly controlled T2D	Cluster RCT: primary care	833	108
Yu et al,^18^ 2020	Canada	Clinicians and patients; diabetes	To assess the effect of MyDiabetesPlan on decisional conflict, diabetes distress, health-related quality of life, and patient assessment of chronic illness care at the individual patient level	Cluster RCT: primary care	213	53

Abbreviations: AHRQ, Agency for Healthcare Research and Quality; BP, blood pressure; CCM, chronic care model; CHD, coronary heart disease; CHW, community health worker; CVD, cardiovascular disease; DECADE, decision aid, action planning, and follow-up support for patients to reduce the 10-year risk of cardiovascular diseases; EDS, electronic decision support; EHR, electronic health record; GP, general practitioner; HbA_1c_, hemoglobin A_1c_; LDL-C, low-density lipoprotein cholesterol; NR, not reported; PDA, patient decision aid; PRO, patient-reported outcome; PROM, patient-reported outcome measure; RCT, randomized clinical trial; SDM, shared decision-making; T1D, type 1 diabetes; T2D, type 2 diabetes; TTE, time-to-event.

### Quality Appraisal

Among the included studies, 30 (50.8%) were rated fair,^16,19,20,24,25,28,29,30,33,34,35,37,38,39,41,42,44,45,46,48,49,50,59,62,63,65,66,67,73,74^ 29 (49.2%) were rated poor,^17,18,21,22,23,26,27,31,32,36,40,43,47,51,52,53,54,55,56,57,58,60,61,64,68,69,70,71,72^ and none were rated good. These ratings were primarily driven by inadequate blinding, unclear assessment of adherence to protocols, and high study attrition rates. Details of the quality assessment can be found in eTable 1 in Supplement 1.

### Interventions for Patients and Clinicians

Interventions for patients and clinicians are summarized in Table 2 and eTable 3 in Supplement 1. The SDM interventions were directed toward both patients and clinicians in 29 studies (49.2%),^17,18,19,21,23,30,32,33,34,36,37,38,39,41,42,43,45,52,53,59,61,62,63,64,66,67,72,73,74^ 29 studies (49.2%) exclusively targeted patients,^16,20,22,24,25,27,28,29,31,35,40,44,46,47,48,49,50,51,54,55,56,57,58,60,65,68,69,70,71^ and only 1 study (1.6%) targeted clinicians.^26^ Regarding the frequency of interventions, 18 studies (30.5%) reported using a one-time intervention,^19,20,21,26,32,34,36,40,42,50,57,61,66,68,69,70,71,72^ whereas others included interventions that were performed between 2 and 7 times during the study period.

**Table 2.** Intervention Characteristics of Included Articles

Study	Location	Delivery mode	Frequency; duration	Patient intervention description (technology, decision aid, and messaging)	Clinician intervention description (training, tools, EHR best practice alerts, and PDA use)
Adarkwah et al,^72^ 2016	Germany	In-person consultation and computer	1-Time intervention, follow-up in 3 mo; NR	Decision aid used to show 10-y CVD risk	Training on use of showing risk in ARRIBA-Herz
				In-person consultation focused on specific medications, dose adjustments, and behavioral measures	Advice on how to communicate risk with patients
Applegate et al,^73^ 2021	US	In-person consultation, telephone consultation, and printed material	4 Monthly visits, followed by 2 quarterly visits; 40 min	SDM intervention focused on identifying the highest-priority unfulfilled clinical goals and monitoring action steps to reach them	Formal training on SDM not provided to intervention staff
				Behavior change techniques	Formal training in using behavior change techniques provided
Bailey et al,^71^ 2016	US	Web-based and recorded video	Once before follow-up; 30 min	Decision aid to show recommendations for diabetes management	NR
				Emails and daily messages to send reminders	
Bailey et al,^70^ 2018	US	In-person consultation and online	Once; NR	Interactive online diabetes decision aid for T2D to address decisions about adding therapy to metformin due to poor glycemic control	NR
Boulware et al,^68^ 2020	US	In-person consultation and telephone consultation	Once; NR	Training in SDM skills provided by a CHW	NR
				Workbook and reminder card provided by a CHW	
Branda et al,^69^ 2013	US	In-person consultation	Once; NR	Decision aids during the clinical encounter	NR
Buhse et al,^66^ 2015	Germany	In-person consultation and web-based or recorded video	Once; 90 min	Evidence-based decision aid for patients on the prevention of heart attack	Training DVD, including basic principles of SDM
				Structured patient teaching by diabetes educators	Training focused on evidence-based practice within the decision aid and patient teaching
				Magnet board to visualize the quantity risk	
Buhse et al,^67^ 2018	Germany	In-person consultation	NR	Evidence-based PDA about primary prevention of myocardial infarction and other diabetes-related complications	6-h Training offered to prepare GPs for consultations in terms of SDM
				Patient-held documentation sheet with patient-defined treatment goals	
Cheng et al,^65^ 2021	China	In-person consultation and telephone consultation	Weekly intervention; 6 wk	Brief intake session, 2 structured face-to-face small group discussion sessions, and 4 telephone-based individualized consultation and maintenance sessions	NR
Cooper et al,^64^ 2011	US	In-person consultation; telephone consultation; printed material; photographic novels; newsletter	Bimonthly and monthly; NR	Pocket-sized diaries provided by CHWs to patients to record appointments, medications, and questions	Physician review of videotape of personal interviews with the simulated patient and completion of CD-ROM or workbook exercises
				Bimonthly photographic novels that reinforce the coaching messages; monthly health education newsletter	
Coronado-Vázquez et al,^62^ 2019	Spain	In-person consultation; telephone consultation; web-based or recorded video	1-Time visit to physician’s office; NR	Decision support tool in paper format aimed at helping patients with decision-making by providing information about the secondary risks associated with inappropriate medications in their treatments	Information on the designed decision support tool and a link to a video about the web-based SDM process provided to physicians
Den Ouden et al,^63^ 2017	Netherlands	In-person consultation	2 Times 12 mo apart; NR	OPTIMAL decision support aid provided on how to (1) consider the pros and cons of 2 almost equally effective evidence-based multifactorial treatments; (2) prioritize treatment targets according to the chosen treatment protocol; and (3) select treatment (medication, lifestyle change, or both)	Training on the SDM approach provided to GPs from the intervention group during just one 2-h training session
					Role-plays used to train GPs on the SDM process
Denig et al,^61^ 2014	Netherlands	In-person consultation and printed material	Once; NR	Decision aid presented, which contained several graphs with individually tailored information on risks and treatment options for multiple risk factors; patients received a printed version	Training course in motivational interviewing offered to all practices
Dwinger et al,^60^ 2020	Germany	Telephone consultation	Every 6 wk; 1 y	SDM sharing information on advantages and disadvantages of health behaviors and a joint decision	NR
				Motivational interviewing	
Eaton et al,^59^ 2011	US	In-person consultation; printed material; web-based or recorded video	NR; 1 h	Patient education toolkit consisting of smoking cessation, weight loss, healthy diets, exercise, and lipid-lowering medication adherence materials	1-h Academic session provided to all practices, which detailed using (1) a patient education toolkit and (2) a personal digital assistant–based decision support tool for each physician
Eckman et al,^58^ 2012	US	In-person consultation; printed material	NR; 70 min	Booklet for the foundation for informed medical decision-making	NR
				DVD/VCR video (about 30 min) with similar information to that in the booklet	
Edwards et al,^57^ 2006	UK	Web-based or recorded video	Once; NR	Web-based video with detailed numeric information (absolute or relative risk, numbers needed to treat) and graphics (bar charts, thermometer scales, crowd figure formats)	NR
Farmer et al,^56^ 2005	UK	In-person consultation; telephone consultation; printed material; web-based or recorded video	3 Times over 9 mo; NR	In-person clinical advice and structured counseling provided by a diabetes specialist nurse in response to real-time blood glucose test results	NR
				Nurse telephone call to patients to identify concerns and problems and possible solutions	
Grant et al,^55^ 2008	US	Web-based or recorded video	NR	SDM module for (1) providing patients with their own clinical information linked to tailored decision support and (2) generating a diabetes care plan	NR
Greenfield et al,^54^ 1988	US	In-person consultation	Twice; 20 min	A 20-min session in which the assistant and the patient reviewed standardized educational materials in the diabetes treatment clinic	NR
Heisler et al,^53^ 2014	US	In-person consultation; telephone consultation; printed material; web-based or recorded video	4 Times during 6 wk; 1.5-2 h	Initial one-on-one, face-to-face session with a CHW and a copy of the printed materials to take home	80 h of Initial training in motivational interviewing–based communication approaches and diabetes self-management support provided to CHWs, with 4-8 h of booster training annually
				CHWs contacted participants twice (at 3 and 6 wk after the session after the initial face-to-face session) by telephone to address any additional questions	
Hsu et al,^52^ 2016	US	In-person consultation; live video conference or telehealth; other (secure text messages)	Weekly; 12 wk	Meeting between patients and their health care clinicians during initial visit; for self-management of diabetes, patients received (1) a tablet computer preloaded with the diabetes management program (including the medication regimen and the initial insulin dose) and (2) a glucose meter that was wirelessly connected to the tablet	Tablet to model expert decision-making
					Combination of virtual visits (real-time video and voice communication along with shared screen control), asynchronous text messages, or custom features in the software for making collaborative decisions and communicating dosage recommendations
Hu et al,^51^ 2021	China	In-person consultation; telephone consultation	5 Times over 12 wk; NR	Basal insulin self-titration decision support program that included 1 baseline in-person dosage setting and decision coaching session to empower adjustment, followed by 5 coaching calls delivered by the same nurse	NR
Jaspers et al,^50^ 2021	Netherlands	In-person consultation; telephone consultation; printed material	Once; NR	A personalized health profile in leaflet form	NR
				USB device containing educational videos	
				Structured telephone consultation enforcing uptake of the information	
Jouni et al,^49^ 2017	US	In-person consultation	NR	Modified version of the Statin Choice decision aid to emphasize that CHD risk assessment was probabilistic and not deterministic	NR
				Visit with a genetic counselor and a physician in the cardiovascular health clinic	
Karagiannis et al,^48^ 2016	Greece	In-person consultation	Initial visit, 12 wk, and 24 wk; 6 mo	Greek version of the Diabetes Medication Choice decision aid used by clinicians and patients during the initial clinical encounter	NR
				Physicians presented patients with 7 cards and asked which cards they preferred to discuss	
Kask-Flight et al,^47^ 2021	Estonia	In-person consultation	NR; 3 mo	Computer-based decision aid program, ARRIBA-Herz, with interactive visual prompts used to present 10-y morbidity risk for heart attack and stroke based on the Framingham algorithm	NR
Keyserling et al,^46^ 2014	US	In-person consultation; web-based or recorded video	7 Times; 45-60 min, followed by 15-30 min	Decision aid used to calculate 10-y CVD risk factors, educate participants about their CHD risk factors and the pros and cons of risk-reducing strategies, and show participants the potential CHD risk reduction by changing ≥1 CVD risk factor	NR
				Counseling tailored to the choice of risk-reducing strategy	
Koelewijn-Van Loon et al,^44^ 2009	Netherlands	In-person consultation; telephone consultation; printed material	Twice; 15-20 min	Risk communication tool used by nurses in an in-person meeting to present 10-y risk of CVD mortality, in which they (1) explained the options for risk reduction and gave patients a decision aid to review at home and (2) guided patients in formulating their main personal goals for lifestyle change or medication in telephone consultation using the motivational interviewing method	NR
Koelewijn-Van Loon et al,^45^ 2010	Netherlands	In-person consultation; telephone consultation	Twice; 20 min	Patients in the intervention group received two 20-min face-to-face consultations regarding SDM and a 10-min telephone consultation	2-d Training course involving risk assessment, risk communication, distribution of a decision support tool, and adapted motivational interviewing
Krones et al,^43^ 2008	Germany	In-person consultation	NR	Decision aid to calculate CVD risk for stroke and myocardial infarction and compare age- and sex-adjusted population risk and the effects of their choice	2 CME sessions emphasizing practical communication strategies and materials to be applied during consultation
				In-person counseling with 6 steps	Practice using a script-like decision aid through role-playing
Kulzer et al,^17^ 2018	Germany	In-person consultation	6 times; NR	SDM intervention involving 6 recurring steps: (1) structured assessment and patient education, (2) structured and therapy-adapted SMBG, (3) structured documentation, (4) systematic analysis, (5) personalized treatment, and (6) treatment effectiveness assessment	Training based on a structured curriculum during four 1-h sessions that included video instruction programs and role-play exercises
Kunneman et al,^42^ 2022	US	In-person consultation	Once; NR	Diabetes Medication Choice conversation aid used by patients and clinicians during the clinical encounter; the aid presents general considerations and adverse effects of diabetes medication, and each topic occupies a card	Training on how to use the conversation aid provided during a 10-min group session, with access to an online demonstration and a 1-page storyboard and option to request ad hoc, one-on-one training during the study
				A 1-page handout version of the conversation aid	
Lauffenburger et al,^41^ 2019	US	Telephone consultation	4 Times; 30 min	Consultation conducted by trained clinical pharmacists using a semistructured call guide	Pharmacist training included script development and role-play exercises
				Based on the call, patients identified 1 of 3 strategies to improve diabetes control: (1) treatment intensification, (2) adherence improvement, or (3) lifestyle improvement	
				Simple pillbox and postcard-sized SDM tool used to prime patients for the telephone consultations	
Lee et al,^40^ 2016	South Korea	In-person consultation; web-based or recorded video	Once; 7-min video and 5-15 min of routine medical care and 5-10 min of smoking cessation counseling and prescription	A 7-min video on smoking cessation information and options and a decision aid	NR
Maindal et al,^39^ 2014	Denmark	Other (in-person individual counseling and group session)	2 Interviews and 8 group sessions; 18 h over 3 mo	2 Individual counseling interviews provided by a nurse	Training on target-driven intensive behavioral and pharmacological treatment provided to GPs
				8 Participant-centered group sessions led by nurses, dieticians, physiotherapists, and GPs, covering lifestyles according to participants’ own goals and requests	
				Final individual session with a nurse	
Mathers et al,^38^ 2012	UK	In-person consultation	NR	PDA reviewed by patients before the consultation	Short training session (between 1 and 2 h) for physicians and nurses on how to use the PANDAs decision aid, the principles of SDM, the evidence for various treatment options for poorly controlled T2D, and essential skills in risk communication
				PDA presented in a consultation with the GP or the practice nurse	
Moin et al,^37^ 2019	US	In-person consultation	NR; 35-45 min	Face-to-face SDM visit with a pharmacist who used a decision aid to describe prediabetes and 4 possible options for diabetes prevention	Pharmacist training in SDM and decision aid use, with quarterly refresher training sessions throughout the trial
				Printed summary report with decision and plan provided to patients at the end of the SDM visit	
				Pharmacist prescription given to patients choosing metformin	
Montgomery et al,^16^ 2003	UK	In-person consultation; printed material; web-based or recorded video	NR; 60 min	Simple decision tree to include likely outcomes of treatment options	NR
				Individual absolute CVD risk calculated and combined with utilities using decision analysis software	
				Printed sheet detailing participants’ CVD risk factors and summarizing the decision analysis	
Mullan et al,^36^ 2009	US	In-person consultation	Once; 3 min	Diabetes Medication Choice decision aid tool (6 cards describing the possible effects of the medications on 6 outcomes) used to enable clinicians to discuss with patients the potential advantages and disadvantages of adding an agent from 1 antihyperglycemic classes to their regimen	Brief demonstration from the study coordinator on how to use the decision aid (lasting <3 min; as seen in the video)
				Copy of the cards in the form of a take-home pamphlet provided to patients	
Naik et al,^35^ 2011	US	In-person consultation; other (group session)	Every 3 wk; 3 mo, 1 h each session	A 4-h group sessions focused on the diabetes ABCs, personalized decision-making goals and action plans on lifestyles and diabetes, proactive patient behavior, effective physician-patient communication, and how to develop and obtain feedback on goals and action plans	NR
				10 min of individual interaction with the study clinician after group sessions	
O’Malley et al,^34^ 2022	US	Printed material	Once; NR	Card to prompt patients to reflect on their specific goals for the medical encounter, prioritize those goals, and engage in a discussion with their physician on their concerns and expectations	1 h of Training on the importance of addressing patient concerns and expectations provided to physicians
Peiris et al,^33^ 2015	Australia	Other (software)	Monthly; 48 min	Point-of-care decision support; risk communication interface; clinical audit tool to assess performance on CVD-related indicators; and quality improvement component comprising peer-ranked data feedback and support to develop strategies to improve performance	Intervention practices received an average of 48 min of support per month comprising on-site training, remote clinical webinars, and help desk services
					Clinical staff trained in use of the tools and received access to a technical support desk
					Bimonthly webinars offered with a focus on the practical demonstrations of the tools
Perestelo-Pérez et al,^32^ 2016	Spain	In-person consultation	Once; 1 h	Decision aid applied by physicians in the intervention group	Group sessions of 1 h, in which physicians were trained to apply the decision aid by a member of the research team
				Take-home copy of decision aid provided to patients	
Prabhakaran et al,^74^ 2019	India	Software and text message	NR; 12 mo	mWellcare system (Android application built on the CommCare platform) to generate a longitudinal trend or summary	Centralized training on the current clinical management guidelines and on-site training for orientation to the mWellcare system provided to physicians
				Pamphlets provided by a nurse to each participant	Nurses trained on the management of hypertension, diabetes, depression, and tobacco/alcohol use, and received a 3-d training on using the mWellcare system and another 2 d of on-site supervision and support
				Short message service reminders for scheduled follow-up visits and medication adherence sent to participants	
Ramallo-Fariña et al,^30^ 2021	Spain	In-person consultation, a paper workbook, a website platform, and text message	Every 3 mo; 2 y	An 8-session conventional, group educational program given by a nurse specializing in diabetes	Complex intervention of knowledge transfer and decision support
				Paper workbook used to monitor lifestyle daily	Intervention included (1) an educational and interactive group program of 2 sessions to update clinical management information and promote patient-centered care, (2) an automated decision aid tool, and (3) monthly computerized graphic feedback
				Website platform to upload paper workbook data weekly	
				Text message use to send personalized feedback according to website information	
Rost et al,^31^ 1991	US	In-person consultation	2 Intervention sessions and 4-mo follow-up; 45 min (first session) and 1 h (second session)	Component 1: 45-min patient activation session facilitated by a nurse before the patient’s discharge to introduce a decision tree about treatment choices in managing diabetes-related problems and to discuss facilitators and barriers of active patient participation and potential strategies	NR
				Component 2: 1-h instructional package, which included self-assessment and 3 modules of question-asking skills, completed by patient at home before next outpatient visit	
Smith et al,^29^ 2008	US	Telemedicine consultation	NR	Specialty advice and evidence-based messages regarding medication management for CVD risk provided to patients and clinicians	NR
Sperl-Hillen et al,^28^ 2018	US	Web-based intervention	NR	Evidence-based treatment options for lipid, BP, weight, tobacco, or aspirin management identified and prioritized by the clinical decision support system based on potential benefit to patients	NR
Swoboda et al,^27^ 2017	US	In-person consultation and telephone consultation	1 In-person session and biweekly telephone call until week 16; NR	1 Motivational interviewing and decision support session and 7 biweekly telephone coaching calls	NR
				Multiple-goal intervention: 1 diet goal and 1 physical activity goal established during the first session, and goals in both domains subsequently set during every coaching call	
				Single-goal intervention: a goal for either a diet-related or physical activity–related behavior set during the first session based on individual preference	
Tinsel et al,^26^ 2013	Germany	NR	Once; NR	NR	SDM training program for GPs included information on arterial hypertension, physician-patient communication and risk communication, the process steps of SDM, motivational interviewing, introduction of a decision table listing options to lower cardiovascular risk score, and use of case vignettes for role-plays simulating physician-patient consultations
Tinsel et al,^25^ 2018	Germany	NR	4 Consultation sessions; NR	DECADE brochures that contained evidence-based decision aids and action plans, and 4 structured follow-up consultations	NR
Tusa et al,^24^ 2021	Finland	In-person consultation	1 Health care visit and 12-mo follow-up; 30-60 min with nurse and 30-40 min with GP	Participatory patient care plan formulated in collaboration with the patient, nurse, and physician during the first health care visit	NR
Tutino et al,^22^ 2017	China	In-person consultation and telephone consultation	1 Intervention session and at least 2 follow-up sessions facilitated by a nurse coordinator; 2-4 h of diabetes education	Web-based JADE portal: assessment module including templates for periodic assessment, risk stratification, personalized reporting, and automated decision support plus nurse-coordinated structured follow-up module including templates for documentation of modifiable risk factors, hypoglycemia, and key events to track clinical progress and reinforce adherence	NR
van Steenkiste et al,^23^ 2007	Netherlands	In-person consultation	1 Training and 2 consultations; 8 mo	A 4-h interactive small group training session for clinicians	4-h Interactive small group training session instructed physicians about the risk table and the key recommendations for treatment of patients at high cardiovascular risk
				Decision support tool for patients consisting of a booklet providing education on absolute 10-y CVD risk consultations with clinicians	Role-play used to allow physicians to practice how to use the decision support tool
Warner et al,^21^ 2015	US	In-person consultation	Once; NR	Decision aid consisting of 3 laminated cards with pros and cons of continuing smoking, attempting temporary abstinence, or attempting to quit	Clinicians delivering the decision aid watched an 8-min video demonstrating the use of the decision aid
Weymiller et al,^20^ 2007	US	In-person consultation	Once and 3-mo follow-up; NR	Statin Choice tailored decision aid presenting estimated 10-y cardiovascular risk, absolute risk reduction with use of statin drugs, and disadvantages of using statin drugs	NR
Wollny et al,^19^ 2019	Germany	In-person consultation	Once; NR	Patients in the intervention group received SDM intervention (including patient-centered communication, decision aid) from the trained GPs	Outreach educational peer visit conducted to sensitize patients’ concepts of disease and their views, attitudes, and behaviors by using patient-centered communication
					Additional training on patient communication skills
					Decision aid used HbA_1c_ levels and associated risk factors to visualize the probability of experiencing macrovascular events and present the effect of antidiabetic medication and lifestyle changes on CVD
Yu et al,^18^ 2020	Canada	In-person consultation	NR	Individualized diabetes-specific goals and strategies for patients generated using the MyDiabetesPlan decision aid, which later resulted in an action plan. At 6 mo, patients were provided with a patient-directed how-to guide and video and directed to update MyDiabetesPlan according to their progress before the appointment	One-on-one 60-min tutorial with access to a 1-page how-to guide and 2-min video
					Subsequent review of the resultant action plan with the patient

Abbreviations: BP, blood pressure; CHD, coronary heart disease; CHW, community health worker; CME, continuing medical education; CVD, cardiovascular disease; DECADE, decision aid, action planning, and follow-up support for patients to reduce the 10-year risk of cardiovascular diseases; EHR, electronic health record; GP, general practitioner; HbA_1c_, hemoglobin A_1c_; JADE, Joint Asia Diabetes Evaluation; NR, not reported; PANDAs, Patient and Decision Aids; PDA, patient decision aid; SDM, shared decision-making; SMBG, self-monitoring of blood glucose; T2D, type 2 diabetes.

Regarding interventions for patients, 47 studies (79.7%) conducted individual consultations^16,17,18,19,20,21,22,23,24,25,27,30,31,32,35,36,37,38,39,40,42,43,44,45,46,47,48,49,50,51,52,53,54,56,58,59,61,62,63,65,66,67,68,69,70,72,73^ and 4 studies (6.8%) utilized group sessions.^30,35,39,65^ Almost half the 59 studies (26 [44.1%]) incorporated workbooks, newsletters, cards, diaries, and other printed materials.^16,20,21,23,25,30,31,32,34,36,37,41,42,44,48,50,53,56,58,59,61,64,67,68,73,74^ In terms of digital intervention formats, 19 (32.2%) studies used websites,^16,22,28,30,40,44,46,47,48,49,53,55,56,59,62,66,70,71,72^ 16 (27.1%) used videos,^16,18,29,40,46,50,52,53,55,56,57,58,59,62,66,71^ 11 (18.6%) used telephone-based interventions,^27,41,44,45,50,53,60,62,65,68,73^ 5 (8.5%) used software applications,^16,33,52,72,74^ 4 (4.8%) used text messages,^30,52,71,74^ and 1 (1.7%) used email.^71^ Among the included studies, 3 (5.1%) integrated behavior change techniques (eg, motivational interviewing) into the SDM interventions.^27,60,73^

In terms of interventions for clinicians, 36 studies (61.0%) provided training on using decision aids.^16,18,19,20,21,22,23,25,28,29,30,31,32,36,37,38,40,41,42,43,44,46,47,48,49,59,61,62,63,66,67,69,70,71,72,74^ In addition, 13 studies (22.0%) provided training to enhance SDM skills^17,18,19,26,30,34,37,38,41,63,64,66,67^; 11 (18.6%) focused on improving communication, educational, and motivational interviewing skills^18,19,26,38,43,45,53,59,61,66,72^; and 3 (5.1%) targeted behavior change techniques.^23,39,73^

### Decision Aid Use

A detailed description of the decision aids included in this systematic review is presented in eTable 4 in Supplement 1. Of the 59 articles, 36 (61.0%) used decision aids to enhance SDM.^16,18,19,20,21,22,23,25,28,29,30,31,32,36,37,38,40,41,42,43,44,46,47,48,49,59,61,62,63,66,67,69,70,71,72,74^ In terms of the formats of the decision aids used among the 36 studies, 19 (52.8%) were online based,^18,19,20,22,28,29,30,32,37,42,43,46,48,49,66,67,69,70,71^ 9 (25.0%) were paper based (including postcards, booklet, and brochures),^21,23,25,36,38,41,44,62,63^ 2 (5.6%) were software applications,^72,73^ 2 (5.6%) utilized used computer screens,^47,61^ 2 (5.6%) used decision-making trees,^16,31^ 1 (2.8%) involved a personal digital assistant,^59^ and 1 (2.8%) utilized used video.^40^ Among the 36 included articles with decision aids, 19 (52.8%) were focused on diabetes.^18,19,20,22,29,30,31,36,37,38,41,42,48,61,66,67,69,70,71^

### Risk Factors Targeted and Outcomes

The majority of included studies (32 [54.2%]) concentrated on diabetes only,^17,18,19,20,22,27,28,29,30,31,35,36,37,38,39,41,42,48,51,52,53,54,55,56,57,61,65,66,67,69,70,71^ followed by multiple cardiovascular risk factors (14 [23.7%]),^23,34,44,45,47,49,58,59,60,62,63,72,73,74^ CVD risk (6 [10.2%]),^24,25,32,33,43,50^ hypertension (4 [6.8%]),^16,26,64,68^ and smoking cessation (3 [5.1%])^27,34,54^ (Table 1 and eTable 5 in Supplement 1). In terms of outcomes reported, 8 studies (13.6%) focused solely on decisional outcomes,^18,19,21,49,65,70,71,72^ 35 (59.3%) measured both cardiovascular risk factors and health behaviors as well as decisional outcomes,^16,17,20,23,25,26,27,30,31,32,34,35,36,38,39,42,43,45,46,48,50,51,52,53,54,55,57,58,60,61,64,66,67,68,69^ and 16 (27.1%) evaluated only cardiovascular risk factors and health behaviors.^22,24,28,29,33,37,40,41,44,47,56,59,62,63,73,74^

Multiple studies reported improvements in decisional outcomes after SDM intervention. Improvements following SDM intervention were reported for decisional conflict (7 of 14 [50.0%]) and patient satisfaction with the decisions or treatment (6 of 14 [42.9%]). Other decisional outcomes reported among the articles that demonstrated improvement following SDM intervention included knowledge (13 of 20 [65.0%]), patient-centeredness (6 of 8 [75.0%]), decision quality (5 of 9 [55.6%]), risk perception (4 of 5 [80.0%]), empowerment (4 of 5 [80.0%]), patient activation (4 of 5 [80.0%]), and diabetes-related distress (4 of 7 [57.1%]).

The effect of SDM interventions on cardiovascular risk factors and CVD risk was inconsistent. Among studies measuring the specific outcomes, improvements were reported for the following: tobacco use (5 of 10 [50.0%]), CVD risk (3 of 9 [33.3%]), diabetes (6 of 23 [26.1%]), dyslipidemia (2 of 14 [14.3%]), hypertension (1 of 18 [5.6%]), and overweight and obesity (2 of 13 [15.4%]). The most frequently reported outcomes were HbA_1c_ and SBP levels (as described in the next section).

Improvements following SDM interventions were reported for the following cardiovascular health behaviors: self-management (3 of 5 [60.0%]), medication management (7 of 12 [58.3%]), physical activity (3 of 8 [37.5%]), nutrition and diet (1 of 4 [25.0%]), and adherence (3 of 18 [16.7%]).

### Meta-Analysis for Decisional Conflict, HbA_1c_, and SBP Levels

**Figure 2.**
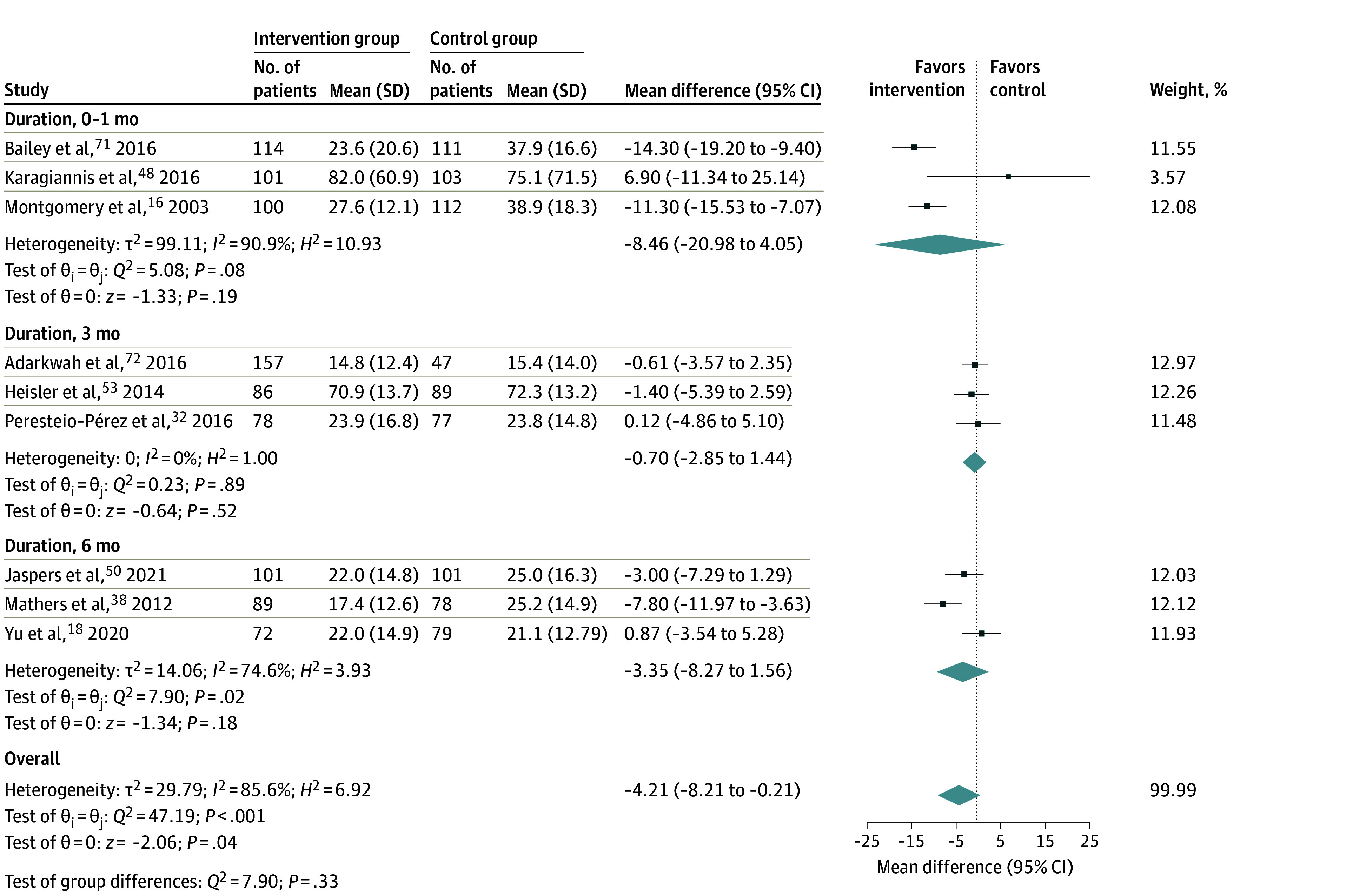
Forest Plot of Mean Differences in Decisional Conflict Between the Shared Decision-Making (SDM)–Based Intervention Group and the Control Group Results of the random-effects Hedges model are presented. The means (SDs) are the outcomes measured at the follow-up time points to perform meta-analyses. The size of the squares is proportional to the weight of each study. Horizontal lines indicate the 95% CI of each study, diamonds are the pooled estimate with 95% CI (weight, 100%), and the vertical dotted line is the line of no effect. Heterogeneity was assessed using the Cochran *Q* test (*H*^2^) and the Higgins *I*^2^ statistic. Interpretation of *I*^2^ values followed Cochrane guidelines (0% to 40%, might not be important; 30% to 60%, may represent moderate heterogeneity; 50% to 90%, may represent substantial heterogeneity; and 75% to 100%, considerable heterogeneity).^10^

The SDM interventions were associated with a decrease of 4.21 points (95% CI, −8.21 to −0.21) in Decisional Conflict Scale scores, with substantial heterogeneity among 9 trials^16,18,32,38,48,50,53,71,72^ (*I*^2^ = 85.6%; *P* < .001; Figure 2). The SDM intervention was associated with a decrease of 0.20% (95% CI, −0.39% to −0.01%) in HbA_1c_ levels, with substantial heterogeneity (*I*^2^ = 84.2%; *P* < .001; eFigure 1 in Supplement 1) observed across 18 trials.^17,24,31,35,36,38,41,42,46,48,51,52,53,54,56,63,66,74^ However, no statistically significant changes were observed in SBP levels for SDM interventions among 10 trials^22,24,25,26,29,34,46,47,63,64^ (mean difference, 0.02 [95% CI, −0.58 to −0.63]; *I*^2^ = 37.0%; eFigure 2 in Supplement 1). Publication bias of these outcomes is presented in eFigures 3 to 5 in Supplement 1.

## Discussion

In this protocol-driven systematic review, we conducted a comprehensive search for RCTs to ensure that only the highest level of evidence regarding the efficacy of SDM interventions was included. This review provides a comprehensive overview of the current state of research on SDM interventions for cardiovascular risk management. The meta-analyses suggested that the SDM intervention was associated with a slight reduction in decisional conflict and HbA_1c_ levels, with substantial heterogeneity.

A limited number of systematic reviews specifically addressing SDM in cardiovascular management have been published.^6,7,8,9^ Some of these reviews focused on multiple cardiac conditions and reported solely on SDM outcomes,^6^ whereas others examined online decision aids for primary CVD prevention^7^ or focused on specific cardiovascular risk factors such as diabetes.^9^ Additionally, some reviews have investigated the effect of computerized decision support systems on cardiovascular risk factors.^8^ To our knowledge, this review is the first comprehensive exploration of SDM in cardiovascular risk factor management. Furthermore, we identified a wide range of SDM interventions that have been tested for managing cardiovascular risk factors. Approximately half of the included studies assessed both SDM process outcomes and cardiovascular risk factors and health behaviors.

A previous systematic review and meta-analysis conducted by Mitropoulou et al^6^ evaluated the effectiveness of interventions to improve SDM in cardiology with a particular focus on SDM process-related outcomes. Mitropoulou et al^6^ found that interventions to increase SDM had a significant effect on reducing decisional conflict and increasing patient knowledge compared with standard of care. Similar to the review by Mitropoulou et al,^6^ we also observed that implementing SDM interventions in cardiovascular risk management has potential to improve various decisional outcomes, including reducing decisional conflict and improving risk perception, empowerment, patient activation, patient-centeredness, disease-related knowledge, distress, decision quality, and satisfaction. In addition, the findings from our meta-analysis of SDM interventions suggest a positive effect on reducing decisional conflict. However, it is important to note that substantial heterogeneity was observed, suggesting variability in the decisional conflict.

In addition to aligning with the findings of Mitropoulou et al,^6^ our findings suggest that use of SDM interventions may also positively affect cardiovascular risk factors such as tobacco use, diabetes, dyslipidemia, hypertension, overweight and obesity, and overall CVD risk. Regarding the association of SDM intervention with HbA_1c_ levels, our meta-analysis suggested a statistically significant reduction of −0.2% across 18 trials. Similar to decisional conflict, substantial heterogeneity was observed, suggesting diversity in the association of SDM interventions with HbA_1c_ levels. However, no statistically significant changes in SBP levels were observed among 10 trials. Although the SDM interventions seemed to be associated with reduced decisional conflict and improved HbA_1c_ levels, the substantial heterogeneity observed warrants further investigation and consideration of potential biases in the reported outcomes. Nevertheless, among trials in our review measuring other cardiovascular risk factors and health behaviors, the findings were inconsistent. This may be explained by the substantial variation across the SDM interventions tested and the methods and time points used to measure those outcomes.

Nearly half of the included studies (29 [49.2%]) implemented interventions targeting both clinicians and patients, whereas the remaining studies focused solely on patients. Despite the protracted nature of cardiovascular risk management efforts, approximately one-third of studies (18 [30.5%]) included one-time SDM interventions without incorporating ongoing interventions to enhance the SDM process. The lack of sustained interventions may hinder the long-term effectiveness of SDM interventions on decisional outcomes and cardiovascular health outcomes.^5^

### Limitations

One limitation of this review is the predominance of studies rated as fair or poor in terms of their methodological quality among the included literature. The absence of studies rated as good suggests potential limitations in the overall quality and rigor of the available evidence. As a result, the reliability and generalizability of these findings may be compromised, as low-quality studies can introduce bias or have methodological flaws that affect the validity of the results. Additionally, the heterogeneity of some outcome measures, such as satisfaction with the decision process and ultimate decisions, across the included studies (eTable 6 and eFigures 6 and 7 in Supplement 1) posed a challenge in synthesizing the findings.

## Conclusions

This systematic review and meta-analysis of 57 RCTs provides a comprehensive overview of the current state of research on SDM interventions for cardiovascular risk management. These findings suggest that the SDM intervention shows promise in alleviating decisional conflict and enhancing HbA_1c_ levels. However, the notable heterogeneity observed underscores the need for additional scrutiny and the careful examination of potential biases inherent in the reported outcomes. High-quality studies are needed to inform the use of SDM to improve cardiovascular risk management in clinical practice.

## Notes

Supplement 1. **eMethods 1.** Search Summary **eMethods 2.** Quality Appraisal **eMethods 3.** Data Synthesis and Data Transformation **eTable 1.** Quality Assessment Using the Quality Assessment of Controlled Intervention Studies Criteria **eTable 2.** Summary of Patient and Clinician Sociodemographic Characteristics and Outcomes **eTable 3.** Summary of Interventions **eTable 4.** Studies Incorporating Decision Aids in the Intervention **eTable 5.** Summary of Outcomes **eTable 6.** Studies Reporting Satisfaction Outcome **eFigure 1.** Forest Plot of HbA_1c_ **eFigure 2.** Forest Plot of Systolic Blood Pressure **eFigure 3.** Funnel Plot of Decisional Conflict **eFigure 4.** Funnel Plot of HbA_1c_ **eFigure 5.** Funnel Plot of Systolic Blood Pressure **eFigure 6.** Forest Plot of Satisfaction About the Decision or Treatment **eFigure 7.** Funnel Plot of Satisfaction About the Decision or Treatment
eReferences

Supplement 2.
Data Sharing Statement
