# Supplementary material for: Shared Decision-Making in Cardiovascular Risk Factor Management: A Systematic Review and Meta-Analysis
Source: JAMA Netw Open. 2024 Mar 26;7(3):e243779. doi: 10.1001/jamanetworkopen.2024.3779 (PMC10966415; doi:10.1001/jamanetworkopen.2024.3779)
Supplement: Supplement 1. — eMethods 1. Search Summary eMethods 2. Quality Appraisal eMethods 3. Data Synthesis and Data Transformation eTable 1. Quality Assessment Using the Quality Assessment of Controlled Intervention Studies Criteria eTable 2. Summary of Patient and Clinician Sociodemographic Characteristics and Outcomes eTable 3. Summary of Interventions eTable 4. Studies Incorporating Decision Aids in the Intervention eTable 5. Summary of Outcomes eTable 6. Studies Reporting Satisfaction Outcome eFigure 1. Forest Plot of HbA1c eFigure 2. Forest Plot of Systolic Blood Pressure eFigure 3. Funnel Plot of Decisional Conflict eFigure 4. Funnel Plot of HbA1c eFigure 5. Funnel Plot of Systolic Blood Pressure eFigure 6. Forest Plot of Satisfaction About the Decision or Treatment eFigure 7. Funnel Plot of Satisfaction About the Decision or Treatment eReferences [file jamanetwopen-e243779-s001.pdf]

# Supplementary Online Content

Elias S, Chen Y, Liu X, et al. Shared decision making in cardiovascular risk factor management: a systematic review and meta-analysis. *JAMA Netw Open*. 2024;7(3):e243779. doi:10.1001/jamanetworkopen.2024.3779

**eMethods 1.** Search Summary

**eMethods 2.** Quality Appraisal

**eMethods 3.** Data Synthesis and Data Transformation

**eTable 1.** Quality Assessment Using the Quality Assessment of Controlled Intervention Studies Criteria

**eTable 2.** Summary of Patient and Clinician Sociodemographic Characteristics and Outcomes

**eTable 3.** Summary of Interventions

**eTable 4.** Studies Incorporating Decision Aids in the Intervention

**eTable 5.** Summary of Outcomes

**eTable 6.** Studies Reporting Satisfaction Outcome

**eFigure 1.** Forest Plot of $HbA_{1c}$

**eFigure 2.** Forest Plot of Systolic Blood Pressure

**eFigure 3.** Funnel Plot of Decisional Conflict

**eFigure 4.** Funnel Plot of $HbA_{1c}$

**eFigure 5.** Funnel Plot of Systolic Blood Pressure

**eFigure 6.** Forest Plot of Satisfaction About the Decision or Treatment

**eFigure 7.** Funnel Plot of Satisfaction About the Decision or Treatment

**eReferences**

This supplementary material has been provided by the authors to give readers additional information about their work.

**eMethods 1.** Search Summary

| **Topic** | Shared Decision Making in Cardiovascular Risk Management | Shared Decision Making in Cardiovascular Risk Management - update | |
|---|---|---|---|
| **Reference Manager** | Covidence | Covidence | |
| **Databases** | MEDLINE via PubMed, CINAHL, Embase, Cochrane, Web of Science, Scopus, ClinicalTrials.gov | MEDLINE via PubMed, CINAHL, Embase, Cochrane, Web of Science, Scopus, ClinicalTrials.gov | |
| **Date Run** | June 9, June 14 | June 23, 2022 | |
| **Total Number of Results** | 8092 | 1273 | 9365 |
| **Number of Duplicates Removed by Stella EndNote** | 2398 | 425 | 2823 |
| **Number of Duplicates Removed by Sherry Zotero** | 146 | - | 146 |
| **Number of Duplicates Removed by Covidence** | 2 | - | 2 |
| **Remaining Number of Results** | 5696 | 848 | 6544 |
| **Search Prepared By** | Stella M. Seal | Stella M. Seal, MLS | |

| | June 9, June 14 | June 23, 2022 | Total |
|---|---|---|---|
| **Registers (n= 104)** | | | |
| Clinicaltrials.gov (n=104) | 104 | 23 | 127 |
| **Databases (n=7,988)** | | | |
| MEDLINE via PubMed (n=2,121) | 2,121 | 277 | 2398 |
| EMBASE (n=1,759) | 1,759 | 269 | 2028 |
| CINAHL (n=662) | 662 | 96 | 758 |
| Cochrane (n= 94) | 867 | 96 | 963 |
| Web of Science (n=1,301) | 1,301 | 204 | 1505 |
| Scopus (n=1,278) | 1,278 | 308 | 1586 |
| Total | 8,092 | 1273 | 9365 |

© 2024 Elias S et al. *JAMA Network Open.*

**PubMed**

| Search number | Query | Results |
|---|---|---|
| 10 | #3 AND #8 AND #9 | 2,121 |
| 9 | (clinical trial[pt] OR randomized controlled trial[pt] OR controlled clinical trial[pt] OR evaluation studies[pt] OR comparative study[pt] OR "intervention studies"[tw] OR Evaluation Studies as Topic[mh] OR program evaluation[mh] OR random allocation[mh] OR random*[tiab] OR double blind*[tiab] OR controlled trial*[tiab] OR clinical trial*[tiab] OR pretest*[tiab] OR pre test*[tiab] OR posttest*[tiab] OR post test*[tiab] OR prepost*[tiab] OR pre post*[tiab] OR controlled before*[tiab] OR "before and after"[tiab] OR interrupted time*[tiab] OR time serie*[tiab] OR intervention*[tiab]) | 5,387,985 |
| 8 | #4 OR (#5 AND #6) OR (#5 AND #7) OR (#6 AND #7) | 84,694 |
| 7 | professional-patient relations[mh] OR ((nurses[mh] OR physicians[mh] OR nurse*[tw] OR physician*[tw] OR clinician*[tw] OR doctor*[tw] OR general practitioner*[tw] OR gps[tw] OR health care professional*[tw] OR healthcare professional*[tw] OR health care provider*[tw] OR healthcare provider*[tw] OR resident*[tw]) AND (patients[mh] OR patient*[tw] OR consumer*[tw] OR people*[tw])) | 852,580 |
| 6 | patient participation[mh] OR patient participation*[tw] OR consumer participation*[tw] OR patient involvement*[tw] OR consumer involvement*[tw] OR ((patient*[ti] OR consumer*[ti]) AND (involvement*[ti] OR involving*[ti] OR participation*[ti] OR participating*[ti])) | 39,461 |
| 5 | decision making[mh:noexp] OR decision support techniques[mh:noexp] OR decision support systems, clinical[mh] OR choice behavior[mh:noexp] OR decision making*[tw] OR decision support*[tw] OR choice behavio*[tw] OR ((decision*[ti] OR choice*[ti]) and (making*[ti] OR support*[ti] OR behavio*[ti])) | 292,055 |
| 4 | "Decision Making, Shared" [mesh] OR shared decision*[tw] OR sharing decision*[tw] OR informed decision*[tw] OR informed choice*[tw] OR decision aid*[tw] OR ((share*[ti] OR sharing*[ti] OR informed*[ti]) and (decision*[ti] OR deciding*[ti] OR choice*[ti])) | 24,471 |
| 3 | #1 OR #2 | 1,674,808 |
| 2 | "Heart Disease Risk Factors"[Mesh] OR ("heart diseases" [mesh] AND "risk factors" [mesh]) OR "Cardiovascular Risk Factors" [tw] OR "Cardiovascular Risk Factor" [tw] OR "Risk Factors for Heart Disease" [tw] OR "Risk Factors for Cardiovascular Disease" [tw] OR "Cardiovascular Risk Score" [tw] OR "Cardiovascular Risk Scores" [tw] OR "Cardiovascular Risk" [tw] OR "Cardiovascular Risks" [tw] OR "Residual Cardiovascular Risk" [tw] OR "Residual Cardiovascular Risks" [tw] | 182,379 |
| 1 | "Overweight"[Mesh] OR "Obesity"[Mesh] OR "Tobacco Use"[Mesh] OR "Smoking"[Mesh] OR "Hypertension"[Mesh] OR "Diabetes Mellitus"[Mesh] OR "Dyslipidemias"[Mesh] OR "Hypercholesterolemia"[Mesh] OR "Tobacco Uses" [tw] OR "Tobacco Chewing" [tw] OR "Tobacco Consumption" [tw] OR "Smoking Behaviors" [tw] OR "Smoking Behavior" [tw] OR "Smoking Habit" [tw] OR "Smoking Habits" [tw] OR "Smoking Behaviors" [tw] OR "Smoking Behavior" [tw] OR "Smoking Habit" [tw] OR "Smoking Habits" [tw] OR "THC Vaping" [tw] OR "E-Cig Use" [tw] OR "E Cig Use" [tw] OR "ECig Use" [tw] OR "Vape" [tw] OR "Vapes" [tw] OR "E-Cigarette Use" [tw] OR "E Cigarette Use" [tw] OR "E-Cigarette Uses" [tw] OR "Nicotine Vaping" [tw] OR "Ecigarette Use" [tw] OR "Electronic Cigarette Use" [tw] OR "Electronic Cigarette Uses" [tw] OR "Overweight" [tw] OR "Obese" [tw] OR "obesity" [tw] OR "High Blood Pressure" [tw] OR "High Blood Pressures" [tw] OR "Elevated blood pressure" [tw] OR " Hypertens*" [tw] OR "Dyslipidemia" [tw] OR "Dyslipoproteinemias" [tw] OR "Dyslipoproteinemia" [tw] OR "Hyperlipemia" [tw] OR "Hyperlipemias" [tw] OR "Hyperlipidemia" [tw] OR "Lipidemia" [tw] OR "Lipidemias" [tw] OR "Lipemia" [tw] OR "Lipemias" [tw] OR "Hypercholesterolemias" [tw] OR "High Cholesterol Levels" [tw] OR "High Cholesterol Level" [tw] OR "Elevated Cholesterol" [tw] OR "Elevated Cholesterols" [tw] OR "Hypercholesteremia" [tw] OR "Hypercholesteremias" [tw] OR smoking [tw] | 1,564,829 |

© 2024 Elias S et al. *JAMA Network Open.*

**Embase**

| No. | Query | Results |
|---|---|---|
| #8 | #1 AND #6 AND #7 | 1759 |
| #7 | #2 OR (#3 AND #4) OR (#3 AND #5) OR (#4 AND #5) | 70726 |
| #6 | 'clinical trial'/exp OR 'randomized controlled trial'/exp OR 'controlled clinical trial'/exp OR 'evaluation study'/exp OR 'comparative study'/exp OR 'intervention study'/exp OR 'program evaluation'/exp OR 'randomization'/exp OR ((((((((((random*:ti,ab,kw OR double:ti,ab,kw) AND blind*:ti,ab,kw OR controlled:ti,ab,kw) AND trial*:ti,ab,kw OR clinical:ti,ab,kw) AND trial*:ti,ab,kw OR pretest*:ti,ab,kw OR pre:ti,ab,kw) AND test*:ti,ab,kw OR posttest*:ti,ab,kw OR post:ti,ab,kw) AND test*:ti,ab,kw OR prepost*:ti,ab,kw OR pre:ti,ab,kw) AND post*:ti,ab,kw OR controlled:ti,ab,kw) AND before*:ti,ab,kw OR 'before and after':ti,ab,kw OR interrupted:ti,ab,kw) AND time*:ti,ab,kw OR time:ti,ab,kw) AND serie*:ti,ab,kw) OR intervention*:ti,ab,kw | 4601654 |
| #5 | 'doctor patient relation'/exp OR 'nurse patient relationship'/exp OR (('nurse'/exp OR 'physician'/exp OR nurse*:ti OR physician*:ti OR clinician*:ti OR doctor*:ti OR 'general practitioners':ti OR gps:ti OR 'health care professionals':ti OR 'healthcare professionals':ti OR 'health care providers':ti OR 'healthcare providers':ti OR resident*:ti) AND ('patient'/exp OR patient*:ti OR consumer*:ti OR people*:ti)) | 535189 |
| #4 | 'patient participation'/exp OR 'patient participation':ti,ab,kw OR 'consumer participation':ti,ab,kw OR 'patient involvement':ti,ab,kw OR 'consumer involvement':ti,ab,kw OR ((patient*:ti OR consumer*:ti) AND (involvement*:ti OR involving*:ti OR participation*:ti OR participating*:ti)) | 46386 |
| #3 | 'clinical decision making'/exp OR 'decision making'/exp OR 'decision support system'/exp OR 'ethical decision making'/exp OR 'family decision making'/exp OR 'medical decision making'/exp OR 'patient decision making'/exp OR 'decision making':ti,ab,kw OR 'decision support':ti,ab,kw OR 'choice behavior':ti,ab,kw OR ((decision*:ti OR choice*:ti) AND (making*:ti OR support*:ti OR behavio*:ti)) | 503272 |
| #2 | 'shared decision making'/exp OR 'shared decision':ti,ab,kw OR 'sharing decision':ti,ab,kw OR 'informed decision':ti,ab,kw OR 'informed choice':ti,ab,kw OR 'decision aid':ti,ab,kw OR ((share*:ti OR sharing*:ti OR informed*:ti) AND (decision*:ti OR deciding*:ti OR choice*:ti)) | 27539 |
| #1 | 'cardiovascular risk'/exp OR 'cardiovascular risk factor'/exp OR 'heart disease risk factor'/exp OR 'hypertension'/exp OR 'diabetes mellitus'/exp OR 'dyslipidemia'/exp OR 'hypercholesterolemia'/exp OR ('heart disease'/exp AND 'risk factor'/exp) OR 'obesity'/exp OR 'tobacco use'/exp OR 'smoking'/exp OR 'cardiovascular risk factors':ti,ab,kw OR 'cardiovascular risk factor':ti,ab,kw OR 'risk factors for heart disease':ti,ab,kw OR 'risk factors for cardiovascular disease':ti,ab,kw OR 'cardiovascular risk score':ti,ab,kw OR 'cardiovascular risk scores':ti,ab,kw OR 'cardiovascular risk':ti,ab,kw OR 'cardiovascular risks':ti,ab,kw OR 'residual cardiovascular risk':ti,ab,kw OR 'residual cardiovascular risks':ti,ab,kw OR 'high blood pressure':ti,ab,kw OR 'high blood pressures':ti,ab,kw OR 'elevated blood pressure':ti,ab,kw OR 'hypertens*':ti,ab,kw OR 'dyslipidemia':ti,ab,kw OR 'dyslipoproteinemias':ti,ab,kw OR 'dyslipoproteinemia':ti,ab,kw OR 'hyperlipemia':ti,ab,kw OR 'hyperlipemias':ti,ab,kw OR 'hyperlipidemia':ti,ab,kw OR 'lipidemia':ti,ab,kw OR 'lipidemias':ti,ab,kw OR 'lipemia':ti,ab,kw OR 'lipemias':ti,ab,kw OR 'hypercholesterolemias':ti,ab,kw OR 'high cholesterol levels':ti,ab,kw OR 'high cholesterol level':ti,ab,kw OR 'elevated cholesterol':ti,ab,kw OR 'elevated cholesterols':ti,ab,kw OR 'hypercholesteremia':ti,ab,kw OR 'hypercholesteremias':ti,ab,kw OR 'tobacco uses':ti,ab,kw OR 'tobacco chewing':ti,ab,kw OR 'tobacco consumption':ti,ab,kw OR 'smoking behaviors':ti,ab,kw OR 'smoking behavior':ti,ab,kw OR 'smoking habit':ti,ab,kw OR 'smoking habits':ti,ab,kw OR 'thc vaping':ti,ab,kw OR 'e-cig use':ti,ab,kw OR 'e cig use':ti,ab,kw OR 'ecig use':ti,ab,kw OR 'vape':ti,ab,kw OR 'vapes':ti,ab,kw OR 'e-cigarette use':ti,ab,kw OR 'e cigarette use':ti,ab,kw OR 'e-cigarette uses':ti,ab,kw OR 'nicotine vaping':ti,ab,kw OR 'ecigarette use':ti,ab,kw OR 'electronic cigarette use':ti,ab,kw OR 'electronic cigarette uses':ti,ab,kw OR 'overweight':ti,ab,kw OR 'obese':ti,ab,kw OR 'obesity':ti,ab,kw | 2889550 |

© 2024 Elias S et al. *JAMA Network Open.*

**CINAHL**

| # | Query | Limiters/Expanders | Last Run Via | Results |
|---|---|---|---|---|
| S8 | S1 AND S6 AND S7 | Expanders - Apply equivalent subjects<br>Search modes - Boolean/Phrase | Interface - EBSCOhost Research Databases<br>Search Screen - Basic Search<br>Database - CINAHL Plus with Full Text | 662 |
| S7 | S2 OR (S3 AND S4) OR (S3 AND S5) OR (S4 AND S5) | Expanders - Apply equivalent subjects<br>Search modes - Boolean/Phrase | Interface - EBSCOhost Research Databases<br>Search Screen - Advanced Search<br>Database - CINAHL Plus with Full Text | 28,336 |
| S6 | (MH "Randomized Controlled Trials") OR (MH "Clinical Trials+") OR (MH "Evaluation Research+") OR (MH "Comparative Studies+") OR (MH "Clinical Research+") OR (MH "Experimental Studies+") OR (MH "Program Evaluation") OR (MH "Random Assignment") OR random* OR double blind* OR controlled trial* OR clinical trial* OR pretest* OR pre test* OR posttest* OR post test* OR prepost* OR pre post* OR controlled before* OR "before and after" OR interrupted time* OR time serie* OR intervention* | Expanders - Apply equivalent subjects<br>Search modes - Boolean/Phrase | Interface - EBSCOhost Research Databases<br>Search Screen - Advanced Search<br>Database - CINAHL Plus with Full Text | 1,371,390 |
| S5 | (MH "Professional-Patient Relations") OR MH Nurse Patient Relations OR MH Physician Patient Relations OR ((MH Nurses+ OR MH Physicians+ OR TI Nurse* OR TI Physician* OR TI Clinician* OR TI Doctor* OR TI General Practitioner* OR TI GPs OR TI Health Care Professional* OR TI Healthcare Professional* OR TI Health Care Provider*OR TI Healthcare Provider* OR TI Resident*) AND (MH Patients+ OR TI Patient* OR TI Consumer* OR TI People*)) | Expanders - Apply equivalent subjects<br>Search modes - Boolean/Phrase | Interface - EBSCOhost Research Databases<br>Search Screen - Advanced Search<br>Database - CINAHL Plus with Full Text | 107,744 |
| S4 | (MH "Consumer Participation") OR AB Consumer Participation* OR TI Consumer Participation* OR AB Patient Participation* OR TI Patient Participation* OR AB Patient Involvement* OR TI Patient Involvement*OR AB Consumer Involvement* OR TI Consumer Involvement*OR ((TI Patient*OR TI Consumer*) AND (TI Participating* OR TI Participation* OR TI Involving* OR TI Involvement*)) | Expanders - Apply equivalent subjects<br>Search modes - Boolean/Phrase | Interface - EBSCOhost Research Databases<br>Search Screen - Advanced Search<br>Database - CINAHL Plus with Full Text | 34,685 |
| S3 | (MH "Decision Making") OR MW Decision Support OR AB Decision Making* OR TI Decision Making* OR AB Decision Support* OR TI Decision Support* OR AB Choice Behavio* OR TI Choice Behavio* OR ((TI Decision* OR TI Choice*) AND (TI Making* OR TI Support* OR TI Behavio*)) | Expanders - Apply equivalent subjects<br>Search modes - Boolean/Phrase | Interface - EBSCOhost Research Databases<br>Search Screen - Advanced Search<br>Database - CINAHL Plus with Full Text | 124,191 |
| S2 | (MH "Decision Making, Shared") OR AB Shared Decision* OR TI Shared Decision* OR AB Sharing Decision* OR TI Sharing Decision* OR AB Informed Decision* OR TI Informed Decision* OR AB Informed Choice* OR TI Informed Choice* OR AB Decision Aid* OR TI Decision Aid* OR ((TI Share* OR TI Sharing OR TI Informed*) AND (TI Decision* OR TI Deciding* OR TI Choice*)) | Expanders - Apply equivalent subjects<br>Search modes - Boolean/Phrase | Interface - EBSCOhost Research Databases<br>Search Screen - Advanced Search<br>Database - CINAHL Plus with Full Text | 18,992 |
| S1 | (MH "Obesity+") OR (MH "Smoking+") OR (MH "Cardiovascular Risk Factors+") OR (MH "Hypertension+") OR (MH "Diabetes Mellitus+") OR (MH "Hyperlipidemia+") OR (MH "Hypercholesterolemia+") OR ((MH "Heart Diseases+") AND (MH "Risk Factors+")) OR | Expanders - Apply equivalent subjects<br>Search modes - Boolean/Phrase | Interface - EBSCOhost Research Databases<br>Search Screen - Advanced Search<br>Database - CINAHL Plus with Full Text | 557,808 |

© 2024 Elias S et al. *JAMA Network Open.*

| | | | | |
|---|---|---|---|---|
| | "Cardiovascular Risk Factors" OR "Cardiovascular Risk Factor" OR "Risk Factors for Heart Disease" OR "Risk Factors for Cardiovascular Disease" OR "Cardiovascular Risk Score" OR "Cardiovascular Risk Scores" OR "Cardiovascular Risk" OR "Cardiovascular Risks" OR "Residual Cardiovascular Risk" OR "Residual Cardiovascular Risks" OR "High Blood Pressure" OR "High Blood Pressures" OR "Elevated blood pressure" OR Hypertens* OR "Dyslipidemia" OR "Dyslipoproteinemias" OR "Dyslipoproteinemia" OR "Hyperlipemia" OR "Hyperlipemias" OR "Hyperlipidemia" OR "Lipidemia" OR "Lipidemias" OR "Lipemia" OR "Lipemias" OR "Hypercholesterolemias" OR "High Cholesterol Levels" OR "High Cholesterol Level" OR "Elevated Cholesterol" OR "Elevated Cholesterols" OR "Hypercholesteremia" OR "Hypercholesteremias" OR “Tobacco Uses” OR “Tobacco Chewing” OR “Tobacco Consumption” OR "smoking" OR “THC Vaping” OR “E Cig Use” OR “ECig Use” OR “Vape” OR “Vapes” OR “E Cigarette Use” OR “Nicotine Vaping” OR “Ecigarette Use” OR “Electronic Cigarette Use” OR “Electronic Cigarette Uses” OR “Overweight” OR “Obese” OR “obesity | | | |

© 2024 Elias S et al. *JAMA Network Open.*

## Cochrane

Search Name: SDM in Cardiovascular Risk Management Date Run: 10/06/2021 05:31:24

Comment: with the addition of obesity & smoking

| ID | Search | Hits |
|---|---|---|
| #1 | MeSH descriptor: [Heart Disease Risk Factors] explode all trees | 94 |
| #2 | MeSH descriptor: [Hypertension] explode all trees | 18921 |
| #3 | MeSH descriptor: [Diabetes Mellitus] explode all trees | 32442 |
| #4 | MeSH descriptor: [Dyslipidemias] explode all trees | 7665 |
| #5 | MeSH descriptor: [Hypercholesterolemia] explode all trees | 3503 |
| #6 | MeSH descriptor: [Overweight] explode all trees | 17146 |
| #7 | MeSH descriptor: [Obesity] explode all trees | 14445 |
| #8 | MeSH descriptor: [Tobacco Use] explode all trees | 272 |
| #9 | MeSH descriptor: [Smoking] explode all trees | 6276 |
| #10 | MeSH descriptor: [Heart Diseases] explode all trees | 53673 |
| #11 | MeSH descriptor: [Risk Factors] explode all trees | 24961 |
| #12 | #10 AND #11 | 5471 |
| #13 | "Cardiovascular Risk Factors" OR "Cardiovascular Risk Factor" OR "Risk Factors for Heart Disease" OR "Risk Factors for Cardiovascular Disease" OR "Cardiovascular Risk Score" OR "Cardiovascular Risk Scores" OR "Cardiovascular Risk" OR "Cardiovascular Risks" OR "Residual Cardiovascular Risk" OR "Residual Cardiovascular Risks" OR "High Blood Pressure" OR "High Blood Pressures" OR "Elevated blood pressure" OR " Hypertens*" OR "Dyslipidemia" OR "Dyslipoproteinemias" OR "Dyslipoproteinemia" OR "Hyperlipemia" OR "Hyperlipemias" OR "Hyperlipidemia" OR "Lipidemia" OR "Lipidemias" OR "Lipemia" OR "Lipemias" OR "Hypercholesterolemias" OR "High Cholesterol Levels" OR "High Cholesterol Level" OR "Elevated Cholesterol" OR "Elevated Cholesterols" OR "Hypercholesteremia" OR "Hypercholesteremias" OR “Tobacco Uses” OR “Tobacco Chewing” OR “Tobacco Consumption” OR "smoking" OR “THC Vaping” OR “E Cig Use” OR “ECig Use” OR “Vape” OR “Vapes” OR “E Cigarette Use” OR “Nicotine Vaping” OR “Ecigarette Use” OR “Electronic Cigarette Use” OR “Electronic Cigarette Uses” OR “Overweight” OR “Obese” OR “obesity” | 106496 |
| #14 | #1 OR #2 OR #3 OR #4 OR #5 OR #6 OR #7 OR #8 OR #9 OR #12 OR #13 | 154627 |
| #15 | MeSH descriptor: [Decision Making, Shared] explode all trees | 36 |
| #16 | ((shared OR sharing OR informed) NEAR/2 (decision* OR deciding OR choice*)) OR "decision aid" OR "decision aids" | 4350 |
| #17 | #15 OR #16 | 4350 |
| #18 | MeSH descriptor: [Decision Making] this term only | 2162 |
| #19 | MeSH descriptor: [Decision Support Techniques] this term only | 831 |
| #20 | MeSH descriptor: [Decision Support Systems, Clinical] explode all trees | 397 |
| #21 | MeSH descriptor: [Choice Behavior] this term only | 1306 |
| #22 | ((decision* OR choice*) NEAR/2 (making OR support* OR behavio*)) | 20836 |
| #23 | #18 OR #19 OR #20 OR #21 OR #22 | 20836 |
| #24 | MeSH descriptor: [Patient Participation] explode all trees | 1427 |
| #25 | ((patient* OR consumer*) NEAR/3 (involvement* OR involving* OR participation* OR participat*)) | 21825 |
| #26 | #24 OR #25 | 21825 |
| #27 | MeSH descriptor: [Professional-Patient Relations] explode all trees | 2657 |
| #28 | ((nurse* OR physician* OR clinician* OR doctor* OR "general practitioner" OR "general practitioners" OR "healthcare professional" OR "healthcare professionals" OR "health care provider" OR "health care providers" OR "healthcare provider" OR "healthcare providers") NEAR/3 (patient* OR consumer* OR people*)) | 27483 |
| #29 | #27 OR #28 | 28238 |
| #30 | #14 AND (#15 OR (#23 AND #26) OR (#23 AND #29) OR (#26 AND #29)) | 867 |

© 2024 Elias S et al. *JAMA Network Open.*

**Web of Science**

| |
|---|
| TS=("Cardiovascular Risk Factors" OR "Cardiovascular Risk Factor" OR "Risk Factors for Heart Disease" OR "Risk Factors for Cardiovascular Disease" OR "Cardiovascular Risk Score" OR "Cardiovascular Risk Scores" OR "Cardiovascular Risk" OR "Cardiovascular Risks" OR "Residual Cardiovascular Risk" OR "Residual Cardiovascular Risks" OR "High Blood Pressure" OR "High Blood Pressures" OR "Elevated blood pressure" OR " Hypertens*" OR "Dyslipidemia" OR "Dyslipoproteinemias" OR "Dyslipoproteinemia" OR "Hyperlipemia" OR "Hyperlipemias" OR "Hyperlipidemia" OR "Lipidemia" OR "Lipidemias" OR "Lipemia" OR "Lipemias" OR "Hypercholesterolemias" OR "High Cholesterol Levels" OR "High Cholesterol Level" OR "Elevated Cholesterol" OR "Elevated Cholesterols" OR "Hypercholesteremia" OR "Hypercholesteremias" OR “Tobacco Uses” OR “Tobacco Chewing” OR “Tobacco Consumption” OR “Smoking Behaviors” OR “Smoking Behavior” OR “Smoking Habit” OR “Smoking Habits” OR “Smoking Behaviors” OR “Smoking Behavior” OR “Smoking Habit” OR “Smoking Habits” OR “smoking” OR “THC Vaping” OR “E-Cig Use” OR “E Cig Use” OR “ECig Use” OR “Vape” OR “Vapes” OR “E-Cigarette Use” OR “E Cigarette Use” OR “E-Cigarette Uses” OR “Nicotine Vaping” OR “Ecigarette Use” OR “Electronic Cigarette Use” OR “Electronic Cigarette Uses” OR “Overweight” OR “Obese” OR “obesity” OR ((cardiovascular OR "heart disease" OR "heart diseases") NEAR/3 risk*)) |
| AND |
| (TS=(((shared OR sharing OR informed) NEAR/2 (decision* OR deciding OR choice*)) OR "decision aid" OR "decision aids") OR (TS=(((decision* OR choice*) NEAR/2 (making OR support* OR behavio*))) AND TS=(((nurse* OR physician* OR clinician* OR doctor* OR "general practitioner" OR "general practitioners" OR "healthcare professional" OR "healthcare professionals" OR "health care provider" OR "health care providers" OR "healthcare provider" OR "healthcare providers") NEAR/3 (patient* OR consumer* OR people*)))) OR (TS=(((decision* OR choice*) NEAR/2 (making OR support* OR behavio*))) AND TS=(((patient* OR consumer*) NEAR/3 (involvement* OR involving* OR participation* OR participat*)))) OR (TS=(((nurse* OR physician* OR clinician* OR doctor* OR "general practitioner" OR "general practitioners" OR "healthcare professional" OR "healthcare professionals" OR "health care provider" OR "health care providers" OR "healthcare provider" OR "healthcare providers") NEAR/3 (patient* OR consumer* OR people*))) AND TS=(((patient* OR consumer*) NEAR/3 (involvement* OR involving* OR participation* OR participat*))))) |
| AND |
| TS=(random* OR "double blind" OR ((clinical OR control* OR program OR evaluation OR comparative) NEAR/2 (trial* OR study OR studies)) OR pretest* OR "pre test" OR posttest* OR "post test" OR prepost* OR "pre post" OR "controlled before" OR "before and after" OR "interrupted time" OR "time serie" OR "time series" OR intervention* OR "program evaluation") |
| Timespan: All years. Indexes: SCI-EXPANDED, SSCI, A&HCI, CPCI-S, CPCI-SSH, BKCI-S, BKCI-SSH, ESCI, CCR-EXPANDED, IC. |
| Searched 06/09/2021 |
| 1301 citations |

© 2024 Elias S et al. *JAMA Network Open.*

**Scopus**

| |
|---|
| TITLE-ABS-KEY ((shared W/2 decision*) OR (sharing W/2 decision*) OR (informed W/2 decision*) OR (shared W/3 deciding) OR (sharing W/2 deciding) OR (informed W/2 deciding) OR (shared W/2 choice*) OR (sharing W/2 choice*) OR (informed W/2 choice*) OR "decision aid" OR "decision aids") OR (TITLE-ABS-KEY ((decision* W/2 making) OR (decision W/2 support*) OR (decision W/2 behavio*) OR (choice* W/2 making) OR (choice* W/2 support*) OR (choice* W/2 behavio*)) AND TITLE-ABS-KEY ((nurse* W/3 patient*) OR (nurse* W/3 consumer*) OR (nurse* W/3 people*) OR (physician* W/3 patient*) OR (physician* W/3 consumer*) OR (physican W/3 people*) OR (clinician* W/3 patient*) OR (clinician* W/3 consumer*) OR (clinician* W/3 people*) OR (doctor* W/3 patient*) OR (doctor* W/3 consumer*) OR (doctor* W/3 people*) OR ("general practitioner" W/3 patient*) OR ("general practitioner" W/3 consumer*) OR ("general practitioner" W/3 people*) OR ("healthcare professional" W/3 patient*) OR ("healthcare professional" W/3 consumer*) OR ("healthcare professional" W/3 people*) OR ("health care provider" W/3 patient*) OR ("health care provider" W/3 consumer*) OR ("health care provider" W/3 people*) OR ("healthcare provider" W/3 patient*) OR ("healthcare provider W/3 consumer*) OR (" healthcare AND provider " W/3 people*))) OR (TITLE-ABS-KEY((decision* W/2 making) OR (decision W/2 support*) OR (decision W/2 behavio*) OR (choice* W/2 making) OR (choice* W/2 support*) OR (choice* W/2 behavio*)) AND TITLE-ABS-KEY((patient* W/3 involvement*) OR (consumer* W/3 involvement*) OR (patient W/3 involving*) OR (consumer W/3 involving*) OR (patient* W/3 participation*) OR (consumer W/3 participation*) OR (patient* W/3 participat*) OR (consumer* W/3 participat*))) OR (TITLE-ABS-KEY((nurse* W/3 patient*) OR (nurse* W/3 consumer*) OR (nurse* W/3 people*) OR (physician* W/3 patient*) OR (physician* W/3 consumer*) OR (physican W/3 people*) OR (clinician* W/3 patient*) OR (clinician* W/3 consumer*) OR (clinician* W/3 people*) OR (doctor* W/3 patient*) OR (doctor* W/3 consumer*) OR (doctor* W/3 people*) OR (" general AND practitioner " W/3 patient*) OR (" general AND practitioner " W/3 consumer*) OR (" general AND practitioner " W/3 people*) OR (" healthcare AND professional " W/3 patient*) OR (" healthcare AND professional " W/3 consumer*) OR (" healthcare AND professional " W/3 people*) OR (" health AND care AND provider " W/3 patient*) OR (" health AND care AND provider " W/3 consumer*) OR (" health AND care AND provider " W/3 people*) OR (" healthcare AND provider " W/3 patient*) OR (" healthcare AND provider W/3 consumer*) OR ("healthcare provider" W/3 people*)) AND TITLE-ABS-KEY ((patient* W/3 involvement*) OR (consumer* W/3 involvement*) OR (patient W/3 involving*) OR (consumer W/3 involving*) OR (patient* W/3 participation*) OR (consumer W/3 participation*) OR (patient* W/3 participat*) OR (consumer* W/3 participat*))) |
| AND |
| TITLE-ABS-KEY (random* OR "double blind" OR (clinical W/2 trial*) OR (clinical W/3 study) OR (clinical W/3 studies) OR (control* W/3 trial*) OR (control* W/2 study) OR (control* W/2 studies) OR (evaluation W/2 trial*) OR (evaluation W/2 study) OR (evaluation W/2 studies) OR (comparative W/2 trial*) OR (comparative W/2 study) OR (comparative AND w/2studies) OR pretest* OR "pre test" OR posttest* OR "post test" OR prepost* OR "pre post" OR "controlled before" OR "before and after" OR "interrupted time" OR "time serie" OR "time series" OR intervention* OR "program evaluation") |
| AND |
| TITLE-ABS-KEY ((cardiovascular W/3 risk) OR ("heart disease" W/2 risk*) OR "Cardiovascular Risk Factors" OR "Cardiovascular Risk Factor" OR "Risk Factors for Heart Disease" OR "Risk Factors for Cardiovascular Disease" OR "Cardiovascular Risk Score" OR "Cardiovascular Risk Scores" OR "Cardiovascular Risk" OR "Cardiovascular Risks" OR "Residual Cardiovascular Risk" OR "Residual Cardiovascular Risks" OR "High Blood Pressure" OR "High Blood Pressures" OR "Elevated blood pressure" OR " Hypertens*" OR "Dyslipidemia" OR "Dyslipoproteinemias" OR "Dyslipoproteinemia" OR "Hyperlipemia" OR "Hyperlipemias" OR "Hyperlipidemia" OR "Lipidemia" OR "Lipidemias" OR "Lipemia" OR "Lipemias" OR "Hypercholesterolemias" OR "High Cholesterol Levels" OR "High Cholesterol Level" OR "Elevated Cholesterol" OR "Elevated Cholesterols" OR "Hypercholesteremia" OR "Hypercholesteremias" OR "Tobacco Uses" OR "Tobacco Chewing" OR "Tobacco Consumption" OR "Smoking Behaviors" OR "Smoking Behavior" OR "Smoking Habit" OR "Smoking Habits" OR "Smoking Behaviors" OR "Smoking Behavior" OR "Smoking Habit" OR "Smoking Habits" OR "smoking" OR "THC Vaping" OR "E-Cig Use" OR "E Cig Use" OR "ECig Use" OR "Vape" OR "Vapes" OR "E-Cigarette Use" OR "E Cigarette Use" OR "E-Cigarette Uses" OR "Nicotine Vaping" OR "Ecigarette Use" OR "Electronic Cigarette Use" OR "Electronic Cigarette Uses" OR "Overweight" OR "Obese" OR "obesity") |
| Searched 06/09/2021 |
| 1278 citations |

© 2024 Elias S et al. *JAMA Network Open.*

**Clinicaltrials.gov**

| |
|---|
| 6 Studies found for: **shared decision making \| obesity** |
| 5 Studies found for: **shared decision making \| overweight** |
| 8 Studies found for: **shared decision making \| smoking** |
| 3 Studies found for: **shared decision making \| tobacco use** |
| 1 Study found for: **shared decision making \| vaping** |
| 9 Studies found for: **shared decision making \| cardiovascular risk** |
| 3 Studies found for: **shared decision making \| heart disease risk factors** |
| 7 Studies found for: **shared decision making \| cardiac risk** |
| 10 Studies found for: **shared decision making \| hypertension** |
| 32 Studies found for: **shared decision making \| diabetes** |
| 8 Studies found for: **shared decision making \| dyslipidemia** |
| 7 Studies found for: **shared decision making \| hyperlipemia** |
| 5 Studies found for: **shared decision making \| Hypercholesterolemia** |
| TOTAL 104 |

© 2024 Elias S et al. *JAMA Network Open.*

**Books (n=6)**

| Author | Title | Year | Journal |
|---|---|---|---|
| Geary, D. F.; Schaefer, F. | Comprehensive Pediatric Nephrology | 2008 | Comprehensive Pediatric Nephrology |
| **Parker, M. S.; Kusmirek…** | **Elements of a Successful Lung Cancer-Screening Program** | **2018** | **Lung Cancer Screening** |
| Beswick, A. D.; Brindle, P… | National Institute for Health and Clinical Excellence: Guidance | 2008 | A Systematic Review of Risk Scoring Methods and Clinical Decision Aids U… |
| **McGrady, A.; Moss, D.** | **Pathways to illness, pathways to health** | **2013** | **Pathways to Illness, Pathways to Health** |
| **Sombra, L. R. S.; Anast…** | **Pharmacologic Therapy For Obesity** | **2021** | **StatPearls** |
| Dalton, J.; Thomas, S.;… | The provision of services in the UK for UK armed forces veterans with PTSD: a rapid evidence synthesis | 2018 | Health Services and Delivery Research |

**Book Chapters (n=20)**

| Author | Title | Year | Journal |
|---|---|---|---|
| **Nelson, H. D.; Cantor, A…** | **Achieving Health Equity in Preventive Services** | **2019** | **AHRQ Comparative Effectiveness Reviews** |
| **Kumanyika, S. K.** | **Assembling Evidence and Informing Decisions** | **2010** | **Bridging the Evidence Gap in Obesity Prevention: A Framework to In** |
| **Jackson, M. L.; Howard…** | **Cognition and daytime functioning in sleep-related breathing disorders** | **2011** | **Human Sleep and Cognition, Pt Ii: Clinical and Applied Research** |
| **Muhlhauser, I.** | **Evidence-based patient education in diabetes and beyond-application to other chronic diseases - From o…** | **2005** | **Embedding Education into Diabetes Practice** |
| **Cheng, S. L.; Liao, H. H…** | **Exploring oral cancer patients' preference in medical decision making and quality of life** | **2017** | **Studies in Health Technology and Informatics** |
| **Lim, A.; Muir, K.; Giaco…** | **Glaucoma** | **2015** | **The Ophthalmol. Clin. Trials Handb.** |
| **Delichatsios, H. K.; Pitt…** | **Integrating Nutrition Education into Clinical Practice** | **2020** | **Nutrition Education: Strategies for Improving Nutrition and Healthy E** |
| **Zhang, Y.; Padman, R.** | **An interactive platform to visualize data-driven clinical pathways for the management of multiple chronic…** | **2017** | **16th World Congress of Medical and Health Informatics: Precision He** |
| **Streja, E.; Streja, D. A.** | **Management of Dyslipidemia in the Elderly** | **2000** | **Endotext** |
| **Leweke, F. M.; Odorfer,…** | **Medical needs in the treatment of psychotic disorders** | **2012** | **Handbook of Experimental Pharmacology** |
| **Panigrahi, R.; Joshi, B. L.** | **Oh no, someone get the NO!** | **2010** | **Core Clinical Competencies in Anesthesiology: A Case-Based Appro** |
| **Kukafka, R.; Jeong, I. C…** | **Optimizing Decision Support for Tailored Health Behavior Change Applications** | **2015** | **15th World Congress on Health and Biomedical Informatics, MEDINF** |
| **Burkhardt, H.; Koffner,…** | **PATIENT EDUCATION IN AN ACUTE CARE SETTING. FEASABILITY AND MEDIUM-TIME EFFECTS OF A S…** | **2009** | **Multimedia in Education and Special Education** |
| **Deffenbacher, J. L.** | **Psychosocial interventions: Anger disorders** | **2003** | **Aggression: Psychiatric Assess. and Treatment** |
| Ambrose, K. R.; Atzeni, F. | The Relevance of Preoperative Education Among Healthcare Providers, Family Caregivers, and Patients With S… | 2018 | Handbook of Systemic Autoimmune Diseases |
| **Bui, T.** | **Renal considerations in palliative medicine** | **2013** | **Hospice Care: Health Serv., Qual. and Clin. Manage.** |
| **Witkop, C. T.** | **Shared decision making and labor management in parturients** | **2014** | **Obes. Dur. Pregnancy in Clinical Practice** |
| **Njunju, E. M.; Mazaba,…** | **Suicidal ideation among adolescents attending junior high school in suriname** | **2017** | **Suicide: A Global View on Suicidal Ideation among Adolescents** |
| **Mitchell, E.; Sullivan, F…** | **Using electronic patient records to inform strategic decision making in primary care** | **2004** | **Medinfo 2004: Proceedings of the 11th World Congress on Medical In** |
| **Rodriguez-Araujo, G.;…** | **Utility of invasive and non-invasive cardiovascular research methodologies in drug development for diab…** | **2019** | **Transl. Res. Methods in Diabetes, Obes., and Nonalcoholic Fatty Live** |

**Conference Abstracts (n=6)**

| Author | Title | Year | Journal |
|---|---|---|---|
| **Paredes, S.; Rocha, T.;…** | **cardioRisk Project: Clinical Validation Applied To a Portuguese Population** | **2015** | **2015 International Conference on Education, Management and Systen** |
| **Tipprom, A.; Soontornp…** | **Development of a Rehabilitation Monitoring System on Web-Based Application for Patients with Knee Pain** | **2018** | **2018 6th International Electrical Engineering Congress** |
| **Andriopoulou, F. G.; Bir…** | **Ef-Zin: A hybrid framework for ubiquitous management of comorbidity and multimorbidity in chronic dis…** | **2013** | **2013 Ieee 13th International Conference on Bioinformatics and Bioeng** |
| **Albini, F.; Liu, X. Q.; Tor…** | **An ICT and mobile Health integrated approach to optimize patients' education on hypertension and its m…** | **2016** | **2016 38th Annual International Conference of the Ieee Engineering in.** |
| **Lee, S.; Bareinboim, E.;…** | **Structural Causal Bandits with Non-Manipulable Variables** | **2019** | **Thirty-Third Aaai Conference on Artificial Intelligence / Thirty-First Inn** |
| **De Mello Ara˙jo, E. F.; S…** | **Using simulations for exploring interventions in social networks modeling physical activity behaviour in…** | **2018** | **8th International Conference on Simulation and Modeling Methodolog** |

© 2024 Elias S et al. *JAMA Network Open.*

| Title | Year | Journal |
|---|---|---|
| Highlights from the 7th Practical Diabetes International Foot Conference: The future of diabetic foot care: Modern management and re... | 2000 | Practical Diabetes International |
| Society of General Internal Medicine - 33rd Annual Meeting | 2010 | Journal of General Internal Medicine |
| Singapore Health and Biomedical Congress, SHBC 2013 | 2013 | Annals of the Academy of Medicine Singapore |
| Abstracts from the 36th Annual Meeting of the Society of General Internal Medicine, SGIM 2013 | 2013 | Journal of General Internal Medicine |
| 2013 CAEP/ACMU Scientific Abstracts, CAEP 2013 | 2013 | Canadian Journal of Emergency Medicine |
| The opportunities and challenges of pragmatic point-of-care randomised trials using routinely collected electronic records: evaluatio... | 2014 | Health Technology Assessment |
| Abstracts from the 37th Annual Meeting of the Society of General Internal Medicine, SGIM 2014 | 2014 | Journal of General Internal Medicine |
| Family Medicine Forum Research Proceedings 2014 | 2015 | Canadian Family Physician |
| HEC 2016 | 2016 | European Journal of Epidemiology |
| Scientific Poster Abstracts Selected for the National Lipid Association 2016 Scientific Sessions | 2016 | Journal of Clinical Lipidology |
| EuroHeartCare 2016 | 2016 | European Journal of Cardiovascular Nursing |
| 延續「腎」命 糖尿病腎病變病人&#3... | 2018 | Tzu Chi Nursing Journal |
| Research on multimorbidity in primary care. Selected abstracts from the EGPRN meeting in Tampere, Finland, 9-12 May 2019 All abstr... | 2019 | European Journal of General Practice |
| General Practice and the Community: Research on health service, quality improvements and training. Selected abstracts from the EG... | 2020 | European Journal of General Practice |
| ICS 2020 LAS VEGAS SCIENTIFIC PROGRAMME | 2020 | Neurourology and Urodynamics |

**Dissertations (n=7)**

| Author | Title | Year |
|---|---|---|
| Greenwood, Deborah A... | Evaluation of a Telehealth Intervention Combining Structured Self-Monitoring of Blood Glucose and Nurse Care Coordination Among People Wit... | 2014 |
| Hebert, Rosemary | Making Homes Smoke-Free: The Impact of an Empowerment Intervention for Parents | 2008 |
| Holman, G. T. | Patient handling: restrictions & conditions | 2007 |
| Kennett, James R. | Improving diabetic outcomes with caring communication: Identifying communication patterning for the human diabetic | 2011 |
| McDonnell, Karen Kane | A Decision Aid to Improve Smoking Abstinence and HRQL for Families Facing Cancer | 2013 |
| Sasichay, T. | The relationship between nurse staffing and patient outcomes | 2001 |
| Schlenk, E. A. | Choice behaviors performed by persons with type II diabetes participating in behavioral analysis with nurses | 1994 |

**Lack Primary Data (n=7)**

| Author | Title | Year | Journal |
|---|---|---|---|
| Jacobson, F. L.; Jaklits... | Computed Tomography Scanning for Early Detection of Lung Cancer | 2018 | Annual Review of M |
| Lands, W. E. M. | Dietary fat and health: The evidence and the politics of prevention: Careful use of dietary fats can improve life and prevent disease | 2005 | Annals of the New Y |
| Harous, S.; Serhani, M.... | Hybrid obesity monitoring model using sensors and community engagement | 2017 | 13th IEEE Internatio |
| Heldt, T.; Zoerle, T.; Tei... | Intracranial Pressure and Intracranial Elastance Monitoring in Neurocritical Care | 2019 | Annual Review of Bi |
| Feliciano, E. M. C.; Kro... | The Obesity Paradox in Cancer: How Important Is Muscle? | 2018 | Annual Review of N |
| Patnode, C. D.; Hender... | U.S. Preventive Services Task Force Evidence Syntheses, formerly Systematic Evidence Reviews | 2021 | Interventions for To |
| | Univentricular Right Heart Support Using a Single Axial Flow Catheter for High Risk Multivessel Percutaneous Coronary Intervention | 2019 | Hirst, Colin |

© 2024 Elias S et al. *JAMA Network Open.*

**eMethods 2.** Quality Appraisal

We used the Quality Assessment Tool for Controlled Intervention Studies Criteria developed by the National Heart, Lung, and Blood Institute of the National Institutes of Health.[1] This tool addresses 14 elements of quality and risk assessment (see below), which provides an overall rating of "good", "fair", or "poor" based on critical appraisal of characteristics that are relevant to high-quality research studies. The greater the risk of bias, the lower the quality rating of the study. High potential for risk of bias translates to a rating of poor quality. A "poor" study was defined as if the study has a "fatal flaw" including high dropout rates, high differential dropout rates, no intention-to-treatment (ITT) analysis, or other unsuitable statistical analysis (e.g., completers-only analysis).[1] A study was rated as "fair" if the study did not meet all criteria and did not have "fatal flaws".[2] Low potential for risk of bias translates to a rating of good quality. A study was defined as "good" if the study did not report on adherence in the treatment group but met all other criteria of the study quality assessment tool.[2] Two researchers independently assessed each article, and any discrepancies in the cross-check were evaluated by a third researcher for consensus.

Quality Assessment Tool for Controlled Intervention Studies Criteria:

Q1. Was the study described as randomized, a randomized trial, a randomized clinical trial, or an RCT?

Q2. Was the method of randomization adequate (i.e., use of randomly generated assignment)?

Q3. Was the treatment allocation concealed (so that assignments could not be predicted)?

Q4. Were study participants and providers blinded to treatment group assignment?

Q5. Were the people assessing the outcomes blinded to the participants' group assignments?

Q6. Were the groups similar at baseline on important characteristics that could affect outcomes (e.g., demographics, risk factors, co-morbid conditions)?

Q7. Was the overall drop-out rate from the study at endpoint 20% or lower of the number allocated to treatment?

Q8. Was the differential drop-out rate (between treatment groups) at endpoint 15 percentage points or lower?

Q9. Was there high adherence to the intervention protocols for each treatment group?

© 2024 Elias S et al. *JAMA Network Open.*

Q10. Were other interventions avoided or similar in the groups (e.g., similar background treatments)?

Q11. Were outcomes assessed using valid and reliable measures, implemented consistently across all study participants?

Q12. Did the authors report that the sample size was sufficiently large to be able to detect a difference in the main outcome between groups with at least 80% power?

Q13. Were outcomes reported or subgroups analyzed prespecified (i.e., identified before analyses were conducted)?

Q14. Were all randomized participants analyzed in the group to which they were originally assigned (i.e., did they use an intention-to-treat analysis)?

Overall quality rating (good, fair, poor). (1) "good": meet all 14 criteria; (2) "fair": did not meet all 14 criteria and had no high dropout rates; (3) “poor”: A “poor” study was defined as if the study has a "fatal flaw"

© 2024 Elias S et al. *JAMA Network Open.*

**eMethods 3.** Data Synthesis and Data Transformation

Data were grouped into different study duration periods based on the trials included for the outcome analysis: immediately to less than 1 month, 3 months, 6 months, 12 months, or 24 months post-intervention. In cases where two follow-up endpoints fell into the same period, the later scores were utilized (e.g., for 3-month and 6-month postoperative endpoints, those at 6 months were used). For decisional conflict, HbA1c, and SBP levels, we used the mean and standard deviations (SD) of the outcomes measured at the follow-up time points to perform meta-analyses. The effect size was presented as the mean difference between experimental intervention and control groups accompanied by the 95% confidence interval (CI). For continuous outcomes such as satisfaction regarding the decision and treatment where scales differed, data were pooled using standardized mean difference (SMD), and their corresponding 95% CIs were computed after standardizing to a scale from 0 to 100, where a higher score indicated a better outcome. Due to the variability in interventions and population demographics, a random-effects model was used for all outcomes.[3]

For studies that only reported standard errors (SE) or 95% CI, confidence interval (CI) or presented the mean change from baseline to the follow-up time point, we employed a transformation method in accordance with Cochrane's handbook [4] to estimate the mean and standard deviations (SD) values. For example, SDs were calculated using the formula: $SD = SE \times \sqrt{n}$. If change scores (such as total scores of decisional conflict scales) were provided, the follow-up scores were calculated by summing the change scores and baseline scores. In instances where the essential data for analysis were not obtainable from articles, supplementary materials, or secondary analysis publications, efforts were made to reach out to corresponding authors for the necessary information. Studies in which analytical data remained inaccessible, despite these efforts, were excluded from the analysis.

Heterogeneity was assessed using Cochran's Q-test and Higgins $I^2$ statistics. The interpretation of $I^2$ values followed Cochrane's handbook guidelines.[4] Hedges' test was employed to evaluate publication bias, and funnel plots were visually examined. Subgroup meta-analyses were carried out for different study durations as reported across the studies. $I^2 < 40\%$ indicated it might not be important, 40%-50% suggested it may represent moderate

heterogeneity, 50%-90% indicated it may represent substantial heterogeneity, and $I^2 > 90\%$ suggested considerable heterogeneity. We assessed publication bias by examining funnel plot asymmetry and performing the Egger test.[5]

© 2024 Elias S et al. *JAMA Network Open.*

**eTable 1.** Quality Assessment Using the Quality Assessment of Controlled Intervention Studies Criteria

| Study | Q1 | Q2 | Q3 | Q4 | Q5 | Q6 | Q7 | Q8 | Q9 | Q10 | Q11 | Q12 | Q13 | Q14 | Overall quality rating | Comment |
|---|---|---|---|---|---|---|---|---|---|---|---|---|---|---|---|---|
| Adarkwah 2016 | Yes | Yes | Yes | Other (CD, NR, NA) | Other (CD, NR, NA) | Other (CD, NR, NA) | Yes | Yes | Yes | Yes | Yes | Other (CD, NR, NA) | Yes | Other (CD, NR, NA) | Poor | Not clear if used ITT analysis, and not reporting of sample size based on power |
| Applegate 2021 | Yes | Yes | Yes | Yes | Other (CD, NR, NA) | No | Yes | Yes | Yes | Yes | Yes | Yes | Yes | Yes | Fair | |
| Bailey 2016 | Yes | No | Yes | Yes | Yes | Yes | Yes | Yes | Yes | Yes | Yes | Yes | Yes | Yes | Poor | Method of randomization was not adequate |
| Bailey 2018 | Yes | Yes | No | No | No | Yes | Yes | Yes | Yes | Yes | Yes | No | Yes | Other (CD, NR, NA) | Poor | Used ITT analysis not reported |
| Boulware 2020 | Yes | Yes | Yes | No | Yes | Yes | No | No | No | Yes | Yes | Yes | Yes | Other (CD, NR, NA) | Poor | Not clear if used ITT analysis, higher then 20% dropout rate, higher then 15% differential drop out rate, and not high adherence to the intervention. |
| Branda 2013 | Yes | Yes | Yes | Yes | Other (CD, NR, NA) | Yes | Yes | Yes | Yes | Yes | Yes | No | Yes | Other (CD, NR, NA) | Poor | Used ITT analysis not reported |
| Buhse 2015 | Yes | Yes | Yes | Yes | Yes | Other (CD, NR, NA) | Yes | Yes | Yes | No | Yes | No | Yes | Yes | Fair | |
| Buhse 2018 | Yes | Yes | Yes | No | Yes | Yes | Yes | Yes | Other (CD, NR, NA) | Yes | Yes | Yes | Yes | Yes | Fair | |
| Cheng 2021 | Yes | Yes | Yes | Other (CD, NR, NA) | Yes | Yes | Yes | Yes | Other (CD, NR, NA) | Yes | Yes | Yes | Yes | Yes | Fair | |
| Cooper 2011 | Yes | Yes | Yes | No | Yes | Yes | Yes | Other (CD, NR, NA) | Other (CD, NR, NA) | Yes | Yes | Other (CD, NR, NA) | Yes | Yes | Poor | Differential drop out rate > 15%, and power not reported |
| Coronado-Vazquez 2019 | Yes | Yes | Other (CD, NR, NA) | Other (CD, NR, NA) | Other (CD, NR, NA) | Yes | Yes | Yes | Yes | Yes | Yes | Yes | Yes | Yes | Fair | |
| Den Ouden 2017 | Yes | Yes | No | No | No | Other (CD, NR, NA) | Yes | Yes | Yes | Yes | Yes | Yes | Yes | Yes | Fair | |

© 2024 Elias S et al. *JAMA Network Open.*

| Study | Q1 | Q2 | Q3 | Q4 | Q5 | Q6 | Q7 | Q8 | Q9 | Q10 | Q11 | Q12 | Q13 | Q14 | Overall quality rating | Comment |
|---|---|---|---|---|---|---|---|---|---|---|---|---|---|---|---|---|
| Denig 2014 | Yes | Yes | Yes | No | Other (CD, NR, NA) | No | No | No | No | Other (CD, NR, NA) | Yes | Yes | Yes | Yes | Poor | Overall drop-out rate > 20% |
| Dwinger 2020 | Yes | Yes | Yes | No | No | Yes | No | No | No | Yes | Yes | Yes | Yes | Yes | Poor | overall drop-out rate > 20% and differential drop out rate > 15% |
| Eaton 2011 | Yes | Yes | Other (CD, NR, NA) | No | Other (CD, NR, NA) | Yes | Yes | Yes | Yes | Yes | Yes | Yes | Yes | Yes | Fair | |
| Eckman 2012 | Yes | Yes | Other (CD, NR, NA) | Other (CD, NR, NA) | Other (CD, NR, NA) | Yes | Yes | Yes | Yes | Yes | Yes | Yes | Yes | Other (CD, NR, NA) | Poor | Used ITT analysis not reported |
| Edwards 2006 | Yes | Yes | Other (CD, NR, NA) | No | No | Yes | Yes | Other (CD, NR, NA) | Other (CD, NR, NA) | Yes | Yes | Yes | Yes | Other (CD, NR, NA) | Poor | No reporting on used ITT analysis, and if the differential drop out rate > 15% or not |
| Farmer 2005 | Yes | Yes | Other (CD, NR, NA) | Yes | Yes | Yes | No | No | Yes | Other (CD, NR, NA) | Yes | Yes | Yes | Yes | Poor | Overall drop-out rate > 20% and differential drop out rate > 15% |
| Grant 2008 | Yes | Yes | Other (CD, NR, NA) | Other (CD, NR, NA) | Other (CD, NR, NA) | No | Other (CD, NR, NA) | Other (CD, NR, NA) | Other (CD, NR, NA) | Other (CD, NR, NA) | Yes | No | Yes | Yes | Poor | Overall drop-out rate and differential drop out rate not reported |
| Greenfield 1988 | Yes | Yes | No | No | Yes | Yes | Yes | Other (CD, NR, NA) | Other (CD, NR, NA) | Yes | Yes | No | Yes | Other (CD, NR, NA) | Poor | No reporting on used ITT analysis, dropout rate and if differential drop out rate > 15%, |
| Heisler 2014 | Yes | Yes | Yes | No | Yes | No | Yes | Yes | Other (CD, NR, NA) | Yes | Yes | Yes | Yes | No | Poor | No ITT analysis or other unsuitable analysis (completers only) |
| Hsu 2016 | Yes | Other (CD, NR, NA) | Other (CD, NR, NA) | Other (CD, NR, NA) | Other (CD, NR, NA) | Yes | No | Yes | Other (CD, NR, NA) | Yes | Yes | Yes | Yes | Yes | Poor | Method of randomization was not reported, drop out rate higher than 20% |
| Hu 2021 | Yes | Yes | Other (CD, NR, NA) | No | Other (CD, NR, NA) | Yes | Yes | Yes | Other (CD, NR, NA) | Yes | Yes | Yes | Yes | Other (CD, NR, NA) | Poor | Used ITT analysis not reported |
| Jaspers 2021 | Yes | Yes | Yes | Other (CD, NR, NA) | Other (CD, NR, NA) | Other (CD, NR, NA) | Yes | Yes | Other (CD, NR, NA) | Other (CD, NR, NA) | Yes | Yes | Yes | Yes | Fair | |
| Jouni 2017 | Yes | Yes | Yes | Other (CD, NR, NA) | Other (CD, NR, NA) | Yes | Yes | Yes | Yes | Yes | Yes | Yes | Yes | Yes | Fair | |

© 2024 Elias S et al. *JAMA Network Open.*

| Study | Q1 | Q2 | Q3 | Q4 | Q5 | Q6 | Q7 | Q8 | Q9 | Q10 | Q11 | Q12 | Q13 | Q14 | Overall quality rating | Comment |
|---|---|---|---|---|---|---|---|---|---|---|---|---|---|---|---|---|
| Karagiannis 2016 | Yes | Yes | No | No | Other (CD, NR, NA) | Yes | Yes | Yes | Yes | Yes | Yes | Yes | Yes | Yes | Fair | |
| Kask-Flight 2021 | Yes | Yes | Other (CD, NR, NA) | Other (CD, NR, NA) | Other (CD, NR, NA) | No | No | Yes | Yes | Yes | Yes | Yes | Yes | Other (CD, NR, NA) | Poor | No clear if used ITT analysis, and higher then 20% dropout rate, no clear info on allocation concealed, blindness, |
| Keyserling 2014 | Yes | Yes | Yes | No | Other (CD, NR, NA) | Yes | Yes | Yes | Yes | Yes | Yes | Yes | Yes | Yes | Fair | |
| Koelewijn-vanLoon 2009 | Yes | Yes | No | No | Other (CD, NR, NA) | No | Yes | Yes | Yes | Other (CD, NR, NA) | Yes | Yes | Yes | Yes | Fair | |
| Koelewijn-vanLoon 2010 | Yes | Yes | Yes | No | Other (CD, NR, NA) | No | Yes | Yes | Yes | Yes | Yes | Yes | Yes | Yes | Fair | |
| Krones 2008 | Yes | Yes | Yes | Other (CD, NR, NA) | No | No | Yes | No | Other (CD, NR, NA) | Other (CD, NR, NA) | Yes | Yes | Yes | Other (CD, NR, NA) | Poor | Not clear if used ITT analysis, higher then 15% differential droop out rate, participants not similar at baseline, not clear if adherence to intervention was high and if other similar interventions were avoided. |
| Kulzer 2018 | Yes | Yes | Other (CD, NR, NA) | Other (CD, NR, NA) | Other (CD, NR, NA) | Other (CD, NR, NA) | Yes | No | Yes | Yes | Yes | Other (CD, NR, NA) | Yes | Yes | Poor | Differential drop rate > 15%, and lack of reporting on many other characteristics. |
| Kunneman 2022 | Yes | Yes | Yes | No | No | Yes | Yes | Yes | Yes | Other (CD, NR, NA) | Yes | No | Yes | Yes | Fair | |
| Lauffenburger 2019 | Yes | Yes | Yes | No | No | Yes | Yes | Yes | Yes | Yes | Yes | Yes | Yes | Yes | Fair | |
| Lee 2016 | Yes | Yes | No | No | No | Yes | Yes | Yes | Yes | Yes | Yes | Yes | No | Other (CD, NR, NA) | Poor | No clear if used ITT analysis, no concealment or blindness and no outcomes reported, or subgroups analyzed prespecified |
| Maindal 2014 | Yes | Yes | Other (CD, NR, NA) | Other (CD, NR, NA) | Yes | Yes | Yes | Yes | Other (CD, NR, NA) | Yes | Yes | No | Yes | Yes | Fair | |

© 2024 Elias S et al. *JAMA Network Open.*

| Study | Q1 | Q2 | Q3 | Q4 | Q5 | Q6 | Q7 | Q8 | Q9 | Q10 | Q11 | Q12 | Q13 | Q14 | Overall quality rating | Comment |
|---|---|---|---|---|---|---|---|---|---|---|---|---|---|---|---|---|
| Mathers 2012 | Yes | Yes | Yes | No | No | Other (CD, NR, NA) | Yes | Yes | Yes | Other (CD, NR, NA) | Yes | Yes | Yes | Yes | Fair | |
| Moin 2019 | Yes | Yes | No | Other (CD, NR, NA) | Other (CD, NR, NA) | No | Yes | Yes | Yes | Yes | Yes | Yes | Yes | Yes | Fair | |
| Montgomery 2003 | Yes | Yes | Yes | No | No | Yes | Yes | No | Yes | Yes | Yes | Yes | Yes | Yes | Fair | Differential drop rate not reported but met all other important features |
| Mullan 2009 | Yes | Yes | Yes | No | No | Other (CD, NR, NA) | Yes | Yes | Yes | Other (CD, NR, NA) | Yes | Yes | Yes | Other (CD, NR, NA) | Poor | Used ITT analysis not reported |
| Naik 2011 | Yes | Yes | Yes | Other (CD, NR, NA) | Other (CD, NR, NA) | Yes | Yes | Yes | Yes | Yes | Yes | No | Yes | Yes | Fair | |
| O'Malley 2022 | Yes | Yes | Other (CD, NR, NA) | No | Other (CD, NR, NA) | Other (CD, NR, NA) | Yes | Yes | Yes | Other (CD, NR, NA) | Yes | Yes | Yes | Yes | Fair | |
| Peiris 2015 | Yes | Yes | Yes | Other (CD, NR, NA) | Yes | Yes | Yes | Yes | Yes | Yes | Yes | Yes | Yes | Yes | Fair | |
| Perestelo-PÈrez 2016 | Yes | Yes | Yes | Other (CD, NR, NA) | Other (CD, NR, NA) | No | No | Yes | Other (CD, NR, NA) | Other (CD, NR, NA) | Yes | Other (CD, NR, NA) | Yes | No | Poor | No ITT analysis |
| Prabhakaran 2019 | Yes | Yes | Yes | No | No | Yes | Yes | Yes | Yes | Yes | Yes | Yes | Yes | Yes | Fair | |
| Ramallo-Fariña 2021 | Yes | Yes | Other (CD, NR, NA) | No | Other (CD, NR, NA) | Other (CD, NR, NA) | Yes | Yes | Yes | Other (CD, NR, NA) | Yes | Other (CD, NR, NA) | Yes | Yes | Fair | |
| Rost 1991 | Yes | Yes | Other (CD, NR, NA) | No | Yes | Yes | Yes | Yes | Other (CD, NR, NA) | Yes | Yes | Yes | Yes | Other (CD, NR, NA) | Poor | Used ITT analysis not reported |
| Smith 2008 | Yes | Yes | Yes | No | Yes | Yes | Yes | Yes | Other (CD, NR, NA) | Yes | Yes | Other (CD, NR, NA) | Yes | Yes | Fair | |
| Sperl-Hillen 2018 | Yes | Yes | No | No | No | Yes | Yes | Yes | Yes | Yes | Yes | Yes | No | Yes | Fair | |
| Swoboda 2017 | Yes | Yes | Yes | Yes | Yes | Yes | Yes | Yes | Other (CD, NR, NA) | Yes | Yes | No | Yes | No | Poor | No ITT analysis |

© 2024 Elias S et al. *JAMA Network Open.*

| Study | Q1 | Q2 | Q3 | Q4 | Q5 | Q6 | Q7 | Q8 | Q9 | Q10 | Q11 | Q12 | Q13 | Q14 | Overall quality rating | Comment |
|---|---|---|---|---|---|---|---|---|---|---|---|---|---|---|---|---|
| Tinsel 2013 | Yes | Yes | Yes | No | Other (CD, NR, NA) | No | No | No | Other (CD, NR, NA) | Other (CD, NR, NA) | Yes | No | Yes | Yes | Poor | Overall drop-out rate > 20% and differential drop rate > 15% |
| Tinsel 2018 | Yes | Yes | Other (CD, NR, NA) | No | No | No | Yes | Yes | Yes | Other (CD, NR, NA) | Yes | No | Yes | Yes | Fair | |
| Tusa 2021 | Yes | Yes | Yes | No | No | Yes | Yes | Yes | Yes | Yes | Yes | Yes | Yes | Yes | Fair | |
| Tutino 2017 | Yes | Yes | No | No | No | Yes | No | Yes | Other (CD, NR, NA) | Other (CD, NR, NA) | Other (CD, NR, NA) | No | Yes | No | Poor | No ITT analysis |
| vanSteenkiste 2007 | Yes | Yes | Yes | No | No | No | Yes | Yes | Yes | Yes | Yes | Yes | Yes | Other (CD, NR, NA) | Poor | Used ITT analysis not reported |
| Warner 2015 | Yes | Yes | Other (CD, NR, NA) | Other (CD, NR, NA) | Other (CD, NR, NA) | Yes | Yes | Yes | Yes | Yes | Yes | Yes | Other (CD, NR, NA) | Other (CD, NR, NA) | Poor | Used ITT analysis not reported |
| Weymiller 2007 | Yes | Yes | Yes | Yes | Other (CD, NR, NA) | Yes | Yes | Yes | Yes | Other (CD, NR, NA) | Yes | Yes | Yes | Yes | Fair | |
| Wollny 2019 | Yes | Yes | Yes | No | Yes | Yes | Yes | Yes | Yes | Other (CD, NR, NA) | Yes | Yes | Yes | Yes | Fair | |
| Yu 2020 | Yes | Yes | No | Other (CD, NR, NA) | Other (CD, NR, NA) | Yes | No | Yes | Yes | Yes | Yes | Yes | Yes | Yes | Poor | Overall drop out rate > 20% |

"CD, NR, NA," stands for "Cannot Determine, Not Relevant, Not Applicable."
ITT: intent-to-treatment
Q1. Was the study described as randomized, a randomized trial, a randomized clinical trial, or an RCT?
Q2. Was the method of randomization adequate (i.e., use of randomly generated assignment)?
Q3. Was the treatment allocation concealed (so that assignments could not be predicted)?
Q4. Were study participants and providers blinded to treatment group assignment?
Q5. Were the people assessing the outcomes blinded to the participants' group assignments?
Q6. Were the groups similar at baseline on important characteristics that could affect outcomes (e.g., demographics, risk factors, co-morbid conditions)?
Q7. Was the overall drop-out rate from the study at endpoint 20% or lower of the number allocated to treatment?
Q8. Was the differential drop-out rate (between treatment groups) at endpoint 15 percentage points or lower?
Q9. Was there high adherence to the intervention protocols for each treatment group?
Q10. Were other interventions avoided or similar in the groups (e.g., similar background treatments)?
Q11. Were outcomes assessed using valid and reliable measures, implemented consistently across all study participants?
Q12. Did the authors report that the sample size was sufficiently large to be able to detect a difference in the main outcome between groups with at least 80% power?
Q13. Were outcomes reported or subgroups analyzed prespecified (i.e., identified before analyses were conducted)?

© 2024 Elias S et al. *JAMA Network Open.*

Q14. Were all randomized participants analyzed in the group to which they were originally assigned (i.e., did they use an intention-to-treat analysis)?
Overall quality rating (good, fair, poor). (1) "good": meet all 14 criteria; (2) "fair": did not meet all 14 criteria and had no high dropout rates; (3) “poor”: A “poor” study was defined as if the study has a "fatal flaw"

© 2024 Elias S et al. *JAMA Network Open.*

**eTable 2.** Summary of Patient and Clinician Sociodemographic Characteristics and Outcomes

| Author Year<br>Country | Patients' and clinician' sociodemographic Characteristics | Outcomes*<br>SDM and Decision making aspects<br>CV risk factors and CVDs<br>CV health behaviors |
|---|---|---|
| Adarkwah<br>2016<br>Germany | **Patients' Characteristics**<br>Age (Mean): I: 57.83 (11.03)<br>C: 58.01 (10.66)<br>Males: I: 87 (55%)<br>C: 88 (60%)<br>Race/Ethnicity: NR<br>Education: Low Education: I: 67 (43), C: 66 (45%)<br><br>**Clinician' Characteristics**<br>Age (Mean): 44.6<br>Males: 24 (59)<br>Race/Ethnicity:<br>White 39 (95)<br>Asian: 1 (2.5)<br>Experience (Mean Years): 14<br>Professions: NR | **Decision making**<br>• Shared decision-making: Confirmed non-inferiority of Time-to-Event illustration<br>• Decisional Conflict Scale: Confirmed non-inferiority of Time-to-Event illustration<br>**CV risk factors and CVDs: NR**<br>**CV health behaviors: NR** |
| Applegate<br>2021<br>United States | Age (Median): I: 50, C: 53<br>Males: I: 87 (55), C: 88 (60)<br>Race/Ethnicity:<br>White I: 6 (9.2), C: 4 (6.0);<br>Black: I: 10 (15.4), C: 15 (22.4);<br>Latino: I: 36 (55.4), Con 41 (61.2);<br>Asians: I: 4 (6.2), C: 3 (4.5)<br>Education: NR | **Decision making: NR**<br>**CV risk factors and CVDs**<br>• Systolic BP: NS<br>• Diastolic BP: NS<br>• LDL cholesterol: NS<br>• BMI: NS<br>• A1c: NS<br>**CV health behaviors:**<br>• Average # fulfilled preventive care goals: 0.53, 95% CI: 0.19–0.86<br>• Number achieved hypertension goal: 1.8, 95% CI: 1.1- 3.2 |
| Bailey<br>2016<br>United States | Age (Mean): I: 53 (13.8), C: 51.6 (11.5)<br>Males: I: 52 (45.6), C: 50 (45.1)<br>Race/Ethnicity:<br>White: 101 (45)<br>Black: 63 (28)<br>Latino: 29 (13)<br>Asians: 14 (6)<br>Native American Or Alaska Native: 3 (1)<br>Education:<br>< High School: I: 14, C: 3<br>High School: I: 29 (25.4), C: 29 (26.1)<br>> High School: I: 56, C: 68 | **Decision making**<br>• Lower decisional conflict: −22.2 [20.6] vs−7.5 [16.6]; p < 0.0001<br>• Self-efficacy: 3.7 (16.7) vs−3.9 (19.2); p < 0.0001<br>• Knowledge gain, % (SD): 35.0 % (22.3) vs 9.9 % (22.2); p < 0.0001<br>• Knowledge confidence, % (SD): 11.0 (12.8) vs 1.6 (8.9); p <0.0001<br>**CV risk factors and CVDs: NR**<br>**CV health behaviors: NR** |

© 2024 Elias S et al. *JAMA Network Open.*

| Author Year Country | Patients' and clinician' sociodemographic Characteristics | Outcomes*<br>SDM and Decision making aspects<br>CV risk factors and CVDs<br>CV health behaviors |
|---|---|---|
| Bailey<br>2018<br>United States | Age (Mean): 53;<br>Males: 52 (46.5%)<br>Race/Ethnicity:<br>White: 53 (46.5)<br>Black: 32 (28.1)<br>Latino: 14 (12.3)<br>Asian: 5 (4.4)<br>Education: Grade School/High School: I: 43 (36); Some College: I: 30 (26); >College Graduate: I: 41 (36) | **Decision making**<br>• Decisional self-efficacy: NS<br>**CV risk factors and CVDs: NR**<br>**CV health behaviors: NR** |
| Boulware<br>2020<br>United States | Age (Mean): 57<br>Males: I: 15 (28.3)<br>C: 13 (24.5)<br>T: 42 (26.1%)<br>Race/Ethnicity:<br>Black: 159 (100)<br>Education: < High School: 62 (39.0)<br>High School: 89 (56.0)<br>> High School: 8 (5) | **Decision making**<br>• Hypertension behavior: NS<br>• Hypertension self-efficacy: NS<br>• Problem-solving: NS<br>• Clinic visit patient-centeredness: NS<br>**CV risk factors and CVDs**<br>• BP outcomes: NS<br>• **CV health behaviors**<br>• BP self-monitor use: NS<br>• Self-management: NS |
| Branda<br>2013<br>United States | **Patients' Characteristics**<br>Age (Mean): I: 57.9 (10.5)<br>C: 57.3 (11.4)<br>Males: I: 37 (70)<br>C: 26 (52)<br>Race/Ethnicity:<br>White I: 53 (100), C: 36 (72)<br>Education: < High School: 22 (44), C: 10 (22); Some College: I: 19 (38), C: 25 (54); College+: I: 9 (18), C: 11 (24)<br><br>**Clinician' Characteristics**<br>Age (Mean): T: 44.6, I: 44.6<br>Males: 24 (59)<br>Race/Ethnicity:<br>White: 39 (95)<br>Asian: 1 (2.5)<br>Experience (Mean Years): I: 14<br>Professions: NR | **Decision making**<br>• Patient engagement in decision making by clinician: 21.4, p=0.01<br>• Discussed about medications: 77% vs. 45%, p<0.001<br>• More knowledge about options: 57% vs. 33%, p=0.002<br>**CV risk factors and CVDs**<br>HbA1c: NS<br>LDL: NS<br>**CV health behaviors**<br>• Adherence: NS |
| Buhse<br>2015<br>Germany | Age (Mean): I: 61.8 (6.5)<br>C: 61.7 (6.5)<br>Females: I: 36 (46.8)<br>C: 36 (46.8) | **Decision making**<br>• Higher levels of risk comprehension: 5.63, 95% CI: 4.82 - 6.44<br>• Realistic expectations: 3.67 95% CI: 3.23 - 4.11 |

© 2024 Elias S et al. *JAMA Network Open.*

| Author Year Country | Patients' and clinician' sociodemographic Characteristics | Outcomes*<br>SDM and Decision making aspects<br>CV risk factors and CVDs<br>CV health behaviors |
|---|---|---|
| | Race/Ethnicity: NR<br>Education: NR | **CV risk factors and CVDs**<br>• Blood pressure - achievement of treatment goal: NS<br>• HbA1c - achievement of treatment goal: 10.1%, p= 0.046<br>• Smoking- achievement of treatment goal: NS<br>**CV health behaviors**<br>• Medication uptake for BP: NS<br>• Medication uptake for statin: NS<br>• Wished to take statins: 28.7%; p Value 0.001 |
| Buhse<br>2018<br>Germany | Age (Mean): I: 59.5, C: 58.7<br>Females: I: 67 (44.4), C: 59 (46.1)<br>Race/Ethnicity: NR<br>Education:<br>< High School: 55 (20)<br>High School: 179 (64)<br>> High School: 45 (16) | **Decision making**<br>• Adequate risk knowledge: AOR 29.3, p=<0.001<br>• Informed choice - statins: AOR 16.6, p= <0.001<br>• Informed choice - BP: AOR 22.2, p= <0.001<br>• Informed choice - HbA1c: AOR 26.0, p= <0.001<br>• Informed choice: smoking: NS<br>• Treatment goal match clinician & patients - BP: -4.0mm Hg, p= 0.005<br>• Treatment Goal match clinician & patients - HBA1c: -0.2, p= 0.003<br>**CV risk factors and CVDs: NR**<br>**CV health behaviors**<br>• Adherence to therapy - antihypertensive: NS<br>• Adherence to therapy - statin: NS |
| Cheng<br>2021<br>China | Age (Mean): I: 56.13, C: 53.91<br>Males: I: 93 (76.86), C: 86 (71.07)<br>Race/Ethnicity: NR<br>Education:<br>< High School: 71 (29.3)<br>High School: 87 (35.9)<br>> High School: 84 (34.7) | **Decision making**<br>• Empowerment level: 0.176, p=0.027<br>• Emotional distress: −0.424, p= 0.027<br>• Diabetes distress: −0.180, p=0.042<br>• Regimen-related distress: −0.397, p= 0.011<br>**CV risk factors and CVDs: NR**<br>**CV health behaviors: NR**<br>**Additional outcome**<br>• Quality of life: 4.151, p=0.004 |
| Cooper<br>2011<br>United States | **Patients' Characteristics**<br>Age (Mean): T: 61.3 I: 59.7 (11.9), C1: 63.7 (11.1), C2: 60.5 (12.0), C3: 62.4 (12.1)<br>Females: 66%<br>Race/Ethnicity:<br>White: 101 (36)<br>Black: 173 (62)<br>Asians: 3 (1)<br>Education: Education, Mean (Sd), Years: I: 11.3 (2.6), C1: 12.2 (2.1), C2: 11.8 (2.4), C3: 12.2 (2.3) | **Decision making**<br>• Physicians participatory decision-making, reported by patients: +6.2 vs. –5.2, p=0.03<br>• Doctor facilitation of patient Involvement: 0.22 vs. −0.17, p = 0.03<br>• Information exchange : 0.32 vs. −0.22, p= 0.005<br>• Patient decision making: NS<br>**CV risk factors and CVDs**<br>• Changes in systolic BP: NS<br>• Changes in diastolic BP: NS<br>**CV health behaviors** |

© 2024 Elias S et al. *JAMA Network Open.*

| Author Year Country | Patients' and clinician' sociodemographic Characteristics | Outcomes*<br>SDM and Decision making aspects<br>CV risk factors and CVDs<br>CV health behaviors |
|---|---|---|
| | **Clinician' Characteristics**<br>Age (Mean): I: 41.8 (6.7), C: 44.3 (10.3)<br>Females: I: 11 (50), C: 11 (58)<br>Race/Ethnicity:<br>White: 18 (43.9)<br>Black: 12 (29.2)<br>Latino: 1 (2.4)<br>Asian: 10 (24.3)<br>Experience (Mean Years): I: 10.0 (6.2), C: 12.6 (8.9)<br>Professions:<br>Physician: 44 (100%) | • Adherence to medications: NS |
| Coronado-Vázquez 2019 Spain | Age (Mean): I: 78.9 (0.94), C: 79.9 (0.73)<br>Males: I: 19 (33.3), C: 25 (38.5)<br>Race/Ethnicity: NR<br>Education: None: 21 (20.1)<br>Primary School: 80 (76.9)<br>Secondary School: 2 (1.9)<br>University: 1 (0.9) | **Decision making: NR**<br>**CV risk factors and CVDs: NR**<br>**CV health behaviors**<br>• Proportion of patients with adapted medication: 32.5% vs. 27.9%, p= 0.008<br>• Probability of medication appropriateness OR=2.8, p=0.008<br>• Appropriateness of medication among patients with good adherence: 62.1% vs. 37.9%, p=0.005<br>• Average of inappropriate medications withdrawn: 0.34, p=0.04 |
| Den Ouden 2017 Netherlands | Age (Mean): I: 70, C: 68.5<br>Males: I: 39(54.2), C: 50(58.8)<br>Race/Ethnicity: NR<br>Education: High: I: 12(16.7), C: 14(17.3)<br>Middle: I: 23(31.9), C: 25(30.9)<br>Low: I: 37(51.4), C: 42(51.9) | **Decision making: NR**<br>**CV risk factors and CVDs**<br>• HbA1c: NS<br>• SBP: NS<br>• Total cholesterol: NS<br>• BMI: NS<br>**CV health behaviors: NR** |
| Denig 2014 Netherlands | Age (Mean): I: 61.8 (8.5), C: 61.5 (8.5)<br>Females: I: 94 (42), C: 54 (46)<br>Race/Ethnicity: NR<br>Education: Not More Than Primary School or Lower Vocational Education: I: 90 (40), C: 45 (38) | **Decision making**<br>• Empowerment: NS<br>• Presentation formats on patient empowerment: NS<br>**CV risk factors and CVDs**<br>• Uncontrolled SBP: NS<br>• Mean SBP: NS<br>• Uncontrolled HbA1c: NS<br>• Mean HbA1c: NS<br>**CV health behaviors**<br>• Lipid regulating drug treatment: NS<br>• Lipid regulating drug treatment with printed version of the decision aid: OR: 3.90, 95% CI: 1.29 to 11.80<br>• SBP intensification of drug treatment: NS |

© 2024 Elias S et al. *JAMA Network Open.*

| Author Year Country | Patients' and clinician' sociodemographic Characteristics | Outcomes*<br>SDM and Decision making aspects<br>CV risk factors and CVDs<br>CV health behaviors |
|---|---|---|
| | | • HbA1c intensification of drug treatment: NS<br>• LDL intensification of drug treatment: NS |
| Dwinger<br>2020<br>Germany | Age (Mean): I: 66.29, C1: 67.28, C2: 66.9 (9.260), C3: 66.60 (9.156)<br>Females, %: I: 54.4, C1: 57.5, C2: 54.7, C3: 55.9<br>Race/Ethnicity: NR<br>Education:<br>Apprenticeship: 63.90%<br>Technical College: 14.10%<br>University: 6.00%<br>Other: 6.90% | **Decision making**<br>• Patient activation: p < 0.001<br>• Health literacy: p < 0.001<br>• Process of behavior change (Stages of Change) p=0.005<br>**CV risk factors and CVDs**<br>• Blood pressure measurement: p < 0.001<br>• BMI: p = 0.009<br>• Smoking: NS<br>**CV health behaviors**<br>• Physical activity (hours per week): p=0.030<br>• Physical activity (metabolic rate per week): p=0.048 |
| Eaton<br>2011<br>United States | **Patients' Characteristics**<br>Age (Mean): I: 54.0 (1.1), C: 52.3 (1.1)<br>Males: I: 39.70%, C: 41.80%<br>Race/Ethnicity:<br>White: I: 95.80%, C: 95.70%<br>Black: I: 1.30%, C: 1.20%<br>Hispanic: I: 1.30%, C: 1.70%<br>Asian: I: 0.70%, C: 0.80%<br>American Indian: I: 0.50%, C: 0.50%<br>Education: NR<br><br>**Clinician' Characteristics**<br>Age (Mean): I: 46.4 (8.4), C: 46.7 (6.3)<br>Males: I: 16 (62%), C: 22 (76%)<br>Race/Ethnicity: NR<br>Experience (Mean Years): I: 15.0 (8.8), C: 15.8 (6.9)<br>Professions: Physicians: 55 (100%) | **Decision making: NR**<br>**CV risk factors and CVDs**<br>• Percentage of patients who achieved LDL goals: NS<br>• Percentage of patients who achieved non-HDL cholesterol goals: NS<br>**CV health behaviors: NR**<br>**Additional outcome**<br>• Screening: NS |
| Eckman<br>2012<br>United States | Age (Mean): T: 59.97, I: 58.49, C: 61.37<br>Females: T: 104 (61.2), I: 57.80%, C: 64.40%<br>Race/Ethnicity:<br>White: 61 (35.9)<br>Black: 109 (64.1)<br>Education:<br>High School Graduate: 58.80% | **Decision making: NR**<br>• Diet information focused on saturated fat and cholesterol intake: NS<br>**CV risk factors and CVDs**<br>• Mean blood pressure: NS<br>• Mean blood pressure: NS<br>• Cigarette smoking: NS |

© 2024 Elias S et al. *JAMA Network Open.*

| Author Year Country | Patients' and clinician' sociodemographic Characteristics | Outcomes*<br>SDM and Decision making aspects<br>CV risk factors and CVDs<br>CV health behaviors |
|---|---|---|
| | | • Average number of cigarettes smoked per day among those who smoked: NS<br>**CV health behaviors**<br>• Take medication: NS<br>• Exercise habits: NS |
| Edwards<br>2006<br>United Kingdom | Age (Mean): NR<br>Females: T: 309 (60.8)<br>Race/Ethnicity: White: 439 (86.4%)<br>Education: NR | **Decision making**<br>• Decisional conflict: NS<br>• Satisfaction with information: NS<br>**CV risk factors and CVDs: NR**<br>**CV health behaviors: NR** |
| Farmer<br>2005<br>United Kingdom | Age (Mean): I: 24.5 (4.2), C: 23.2 ( 4.2)<br>Males: I: 28(59.6), C: 27 (58.7)<br>Race/Ethnicity: NR<br>Education: NR | **Decision making: NR**<br>**CV risk factors and CVDs**<br>• A1C: NS<br>**CV health behaviors: NR** |
| Grant<br>2008<br>United States | Age (Mean): T: 56.1, I: 58.8, C: 53.3<br>Females: T: 49%, I: 43%, C: 56%<br>Race/Ethnicity:<br>White: T: 88%, I: 93%, C: 84%<br>Education: NR | **Decision making: NR**<br>**CV risk factors and CVDs**<br>• Blood pressure changes: NS<br>• LDL-C level: NS<br>**CV health behaviors**<br>• Diabetes mellitus-related medication changes: 53% vs 15% ; p < 0.001<br>• Medication initiation or dosage adjustment for:<br>- hyperglycemia (29% vs 15%;p=0.10)<br>- hypertension (13% vs 0%;p=0.02)<br>- hyperlipidemia (11% vs 0%;p=0.03) |
| Greenfield<br>1988<br>United States | Age (Mean): I: 49.8 (14.7), C: 49.5 (13.0)<br>Females: I: 48%, C: 52%<br>Race/Ethnicity: NR<br>Education: Mean (Sd) Years: I: 13.5 (3.2) Years, C: 13.3 (2.6) Years | **Decision making**<br>• Patient satisfaction: NS<br>• Knowledge: NS<br>• Patient involvement indicators:<br>- Length of visit: NS<br>- # of patient controlling behaviors: 5.06, p< 0.05<br>- Ratio of patient to physician conversational utterances: 6.25, p< 0.05<br>- Effectiveness of patient information seeking: 5.34, p< 0.05<br>**CV risk factors and CVDs**<br>• Glycosylated Hemoglobin: 9.06 vs. 10.61, p<0.01<br>**CV health behaviors**<br>• Self-care: NS<br>• No change or decrease in treatment regimen: 5.58, p<0.01 |

© 2024 Elias S et al. *JAMA Network Open.*

| Author Year Country | Patients' and clinician' sociodemographic Characteristics | Outcomes*<br>SDM and Decision making aspects<br>CV risk factors and CVDs<br>CV health behaviors |
|---|---|---|
| Heisler<br>2014<br>United States | Age (Mean): I: 51, C: 52<br>Females: I: 76%, C: 66%<br>Race/Ethnicity:<br>Black: I: 53%, C: 32%<br>Hispanic: I: 53%, C: 61%<br>Education: Less Than High School: I: 39%, C: 65% | **Decision making**<br>• Medication decisional conflict: NS<br>• Knowledge about antihyperglycemic medications: NS<br>• Satisfaction with clarity of medication information, 22.2 vs 13.0, p = 0.03<br>• Satisfaction with helpfulness of medication information, 21.5 vs 10.2, p = 0.007<br>• Improvement in diabetes distress: 14.1 vs -1.6, p < 0.001<br>• Diabetes care self-efficacy: NS<br>**CV risk factors and CVDs**<br>• HbA1c: NS<br>**CV health behaviors**<br>• Medication adherence: NS |
| Hsu<br>2016<br>United States | Age (Mean): I: 53.3, C: 53.8<br>Males: I: NR C: NR<br>Race/Ethnicity: NR<br>Education: NR | **Decision making**<br>• Satisfaction with diabetes management: 2.1 vs 10.1, p = 0.01<br>**CV risk factors and CVDs**<br>• HbA1c mean decline: p = 0.048<br>• Percentage reaching the glycemic target: NS<br>• Weight: NS<br>**CV health behaviors**<br>• Insulin dose: NS |
| Hu<br>2021<br>China | Age (Mean): I: 53.62 (14.33), C: 54.77 (13.43)<br>Males: I: 263 (60.6%), C: 248(57%)<br>Race/Ethnicity: NR<br>Education: NR | **Decision making**<br>• Diabetes empowerment: 38.53 vs 26.00, p<0.001<br>• Diabetes knowledge:19.15 vs 16.71, p <0.01<br>**CV risk factors and CVDs**<br>• HbA1c: 6.66 vs 7.45, p <0.001<br>• Fasting plasma glucose: 6.33 vs 7.13, p <0.001<br>• Postprandial plasma glucose: 7.72 vs 8.18, p <0.001<br>• Proportion of patients with hypoglycemia events: NS<br>• Changes of body weight: NS<br>• BMI: NS<br>**CV health behaviors**<br>• More diabetes self-care activities: p <0.01 |
| Jaspers<br>2021<br>Netherlands | Age (Mean): I: 66, C1: 66(59-70), C2: 64<br>Males: I: 82%, C1: 86%, C2: 85%<br>Race/Ethnicity: NR<br>Education: NR | **Decision making**<br>• Shared Decision Making: NS<br>• Decisional conflict:<br>individual 10-year absolute CVD risk group vs control: 22 vs 27, p = 0.001<br>CVD-free life-expectancy group vs control: 25 vs 27, p = 0.021 |

© 2024 Elias S et al. *JAMA Network Open.*

| Author Year Country | Patients' and clinician' sociodemographic Characteristics | Outcomes*<br>SDM and Decision making aspects<br>CV risk factors and CVDs<br>CV health behaviors |
|---|---|---|
| | | • Patient Activation: NS<br>• **CV risk factors and CVDs**<br>• Median serum LDL-c levels: NS<br>**CV health behaviors: NR** |
| Jouni<br>2017<br>United States | Age (Mean): I: 59 (4.9), C: 59 (5.2)<br>Males: I: 48 (46.2%), C: 50 (48.5%)<br>Race/Ethnicity: NR<br>Education: College Education Or Higher: I: 53 (56.7%), C: 68 (66%) | **Decision making**<br>• Shared decision-making: NS<br>• Satisfaction with the clinical encounter: NS<br>• Perception of the quality of the discussion: NS<br>• Participation in decision-making: NS<br>• Physician visit satisfaction: NS<br>**CV risk factors and CVDs: NR**<br>**CV health behaviors: NR** |
| Karagiannis<br>2016<br>Greece | Age (Mean): I: 65.8 (11.0), C: 64.4 (11.3)<br>Females: I: 55 (54.5), C: 61 (59.2)<br>Race/Ethnicity: NR<br>Education:<br>≤High School Degree: 147 (73)<br>>High School: 54 (27) | **Decision making**<br>• Overall decisional comfort: NS<br>• Knowledge: NS<br>• Satisfaction with decision made: NS<br>• Satisfaction with the conversation with the clinician: NS<br>**CV risk factors and CVDs**<br>• Change in HbA1c: NS<br>• BMI: NS<br>**CV health behaviors**<br>• Proportion of days covered for all antidiabetic medications: NS<br>• Missed medicine in prior week: NS |
| Kask-Flight<br>2021<br>Estonia | Age (Mean): I: 40.96 (7.22), C: 36.98 (7.93)<br>Males: 100%<br>Race/Ethnicity: NR<br>Education: NR | **Decision making: NR**<br>**CV risk factors and CVDs**<br>• Systolic BP: NS<br>• Systolic BP (Patients with Hypertension Grade 2): − 7.86, p = 0.038<br>• Diastolic BP: NS<br>• Total cholesterol: NS<br>• Reduced the number of cigarettes per day: − 6.05, p = 0.001<br>• BMI: NS<br>**CV health behaviors**: **NR** |
| Keyserling<br>2014<br>United States | Age (Mean): 62 (0.4)<br>Females: 186 (48)<br>Race/Ethnicity:<br>White: 292 (76)<br>Black: 92 (24)<br>Education:<br>< High School: 68(18)<br>High School: 144(37) | **Decision making: NR**<br>**CV risk factors and CVDs**<br>• Framingham Risk Score: −1.0%, p = 0.03<br>• Systolic BP: NS<br>• Diastolic BP: NS<br>• Total cholesterol: NS<br>• HDL-C: NS |

© 2024 Elias S et al. *JAMA Network Open.*

| Author Year Country | Patients' and clinician' sociodemographic Characteristics | Outcomes*<br>SDM and Decision making aspects<br>CV risk factors and CVDs<br>CV health behaviors |
|---|---|---|
| | >High School: 173(45) | • LDL-C: NS<br>• Weight in kg: NS<br>• Smoking: NS<br>• HbA1c, those with diabetes: NS<br>**CV health behaviors**<br>• Dietary intake: NS<br>• Physical activity: NS<br>• Adherence: NS<br>• Taking blood pressure medication: NS<br>• Taking cholesterol medication: NS |
| Koelewijn-van Loon<br>2009<br>Netherlands | **Patients' Characteristics**<br>Age (Mean): I: 56, C: 58<br>Males: I: 130 (43), C: 134 (47)<br>Race/Ethnicity: NR<br>Education: NR<br><br>**Clinician' Characteristics**<br>Age (Mean): I: 38 (7), C: 39 (9)<br>Females: I: 13, C: 11<br>Race/Ethnicity: NR<br>Experience: NR<br>Professions: RNs: 24 (100%) I: 13, C: 11 | **Decision making: NR**<br>**CV risk factors and CVDs**<br>• Cardiovascular 10-year risk: NS<br>• Smoking: NS<br>**CV health behaviors**<br>• Consumption of vegetables/fruit: NS<br>• Physical activity: NS<br>• Intake of fat: NS |
| Koelewijn-van Loon<br>2010<br>Netherlands | **Patients' Characteristics**<br>Age (Mean): T 57, I: 56, C: 58<br>Males: T: 45, I: 43, C: 47<br>Race/Ethnicity: NR<br>Education: NR<br><br>**Clinician' Characteristics**<br>Age (Mean): I: 38, C: 39<br>Females: 24<br>Race/Ethnicity: NR<br>Experience (Mean Years): NR<br>Professions: RNs: 24 (100%) | **Decision making**<br>• Appropriateness of anxiety: 131, $p < 0.01$<br>• More satisfied with the communication (Sum score): 35.4, $p<0.01$<br>• Absolute risk perception (mean): 3.6, $p < 0.01$<br>• Appropriateness of risk perception: NS<br>**CV risk factors and CVDs: NR**<br>• Smoking: OR 1.91, $p = 0.01$<br>**CV health behaviors**<br>• Fat score: NS<br>• Fruit (pieces/week): NS<br>• Vegetables (tablespoons/week): NS<br>• Physical activity: NS |
| Krones<br>2008<br>Germany | **Patients' Characteristics**<br>Age (Mean): I: 59.1, C: 58.6 (12.5)<br>Males: I: 231 (42.0), C: 265 (45.5)<br>Race/Ethnicity: NR<br>Education:<br>No Or Basic Education: 604 (54) | **Decision making**<br>• SDM steps reported: 1.99, $p<0.001$<br>• Patient participation and satisfaction: -0.80, $p<0.001$<br>• Decisional regret was significantly lower at follow-up: 3.39, $p = 0.02$<br>• Knowledge: NS<br>**CV risk factors and CVDs** |

© 2024 Elias S et al. *JAMA Network Open.*

| **Author Year Country** | **Patients' and clinician' sociodemographic Characteristics** | **Outcomes*<br>SDM and Decision making aspects<br>CV risk factors and CVDs<br>CV health behaviors** |
|---|---|---|
| | Medium Education: 337 (30)<br>Higher Education: 182 (16)<br><br>**Clinician' Characteristics**<br>Age (Mean): NR<br>Males: I: 27 (61.4%), C: 26 (55.3%)<br>Race/Ethnicity: NR<br>Experience (Mean Years): NR<br>Professions: Physician: 91 (100%), I: 44 (48%), C: 47 (52%) | • Mean change of CVD risk: NS<br>**CV health behaviors: NR** |
| Kulzer<br>2018<br>Germany | **Patients' Characteristics**<br>Age (Mean): I: 64.5, C: 64.9<br>Males: I: 266 (60.5%), C: 261 (55.9%)<br>Race/Ethnicity: NR<br>Education: <High School: 701 (79)<br>High School/Technical: I: 131 (15)<br>University: 53 (6) | **Decision making**<br>• Diabetes treatment satisfaction, status (Month 12): OR=0.92, p=0.0127<br>• Diabetes treatment satisfaction (Month 12): OR=1.8, p=0.0035<br>• Assessment of the quality of the analysis and discussion of blood glucose values: OR=1.4, p<0.0027<br>• More behavioral/ lifestyle recommendations for diabetes training: OR = 1.8; p = 0.045<br>**CV risk factors and CVDs**<br>• Reduction in HbA1c (at 3, 6, 9 and 12 months, p<0.05); 3 months: 0.2%, p = 0.0054<br>**CV health behaviors**<br>• Therapy adjustments: p < 0.01<br>• Better adherence: OR= 2.4, p=0.0003<br>• Physical activity/exercise: OR = 2.4; p = 0.0063<br>• Nutrition counseling OR = 2.2; p = 0.013 |
| Kunneman<br>2022<br>United States | **Patients' Characteristics**<br>Age (Mean): I: 59 (11), C: 62 (12)<br>Females: I: 72 (38), C: 86 (53)<br>Race/Ethnicity:<br>White: I: 155 (82), C: 139 (86)<br>Black: I: 24 (13), C: 14 (9)<br>Education: High School Or Less: I: 66 (42), C: 50 (35); Vocational/4-Year College Degree: I: 76 (49), C: 84 (59); Graduate Degree: I: 14 (9), C: 8 (6), Adequate Literacy, N(%): I: 21 (13), C: 24 (17)<br><br>**Clinician' Characteristics**<br>Age (Mean): I: 45 (11) , C: 45 (12)<br>Females: I: 26 (54), C: 27 (51)<br>Race/Ethnicity: NR<br>Experience (Mean Years): I: 12, C: 12 | **Decision making**<br>• Knowledge: 6.2, p=0.04<br>• Clinician involvement of patients: 7.3, p=0.003<br>• Decision conflict: NS<br>• Treatment choice: NS<br>• Patient satisfaction: NS<br>• Clinician satisfaction: NS<br>• Encounter length: NS<br>• Perception of being informed: NS<br>• Perception of being supported: NS<br>• Having made a good decision: NS<br>• Being satisfied with the information given: NS<br>**CV risk factors and CVDs**<br>• Glycemic control: NS<br>**CV health behaviors** |

© 2024 Elias S et al. *JAMA Network Open.*

| Author Year Country | Patients' and clinician' sociodemographic Characteristics | Outcomes*<br>SDM and Decision making aspects<br>CV risk factors and CVDs<br>CV health behaviors |
|---|---|---|
| | Professions: Physician: I: 39 (77), C: 46 (87); Advance Practitioner (Pa/Np) : I: 7 (15), C: 6 (11) | • Medication adherence: NS |
| Lauffenburger<br>2019<br>United States | **Patients' Characteristics**<br>Age (Mean): I: 54.9 (8.1), C: 54.6 (8.4)<br>Females: I: 34.60%, C: 39.80%<br>Race/Ethnicity: NR<br>Education: NR | **Decision making: NR**<br>**CV risk factors and CVDs**<br>• Diabetes control: NS<br>• As-treated analyses, diabetes control: -0.48, 95%CI: -0.91, -0.05<br>• As-treated analyses, proportion achieving optimal HbA1c: 1.37, 95%CI: 0.86, 2.17<br>Note: as-treated analysis can introduce bias, therefore these significant results were not included in the intervention and outcomes summary table.<br>**CV health behaviors**<br>• Medication adherence: NS<br>• As-treated analyses, medication adherence: NS<br>• As-treated analyses, proportion achieving optimal adherence: NS |
| Lee<br>2016<br>South Korea | **Patients' Characteristics**<br>Age (Mean): T 48.2 (12.8), I: 48.0 (13.3), C: 48.4 (12.4)<br>Males: T: 381 (92.0), I: 184 (94.4), C: 197 (90.0)<br>Race/Ethnicity: NR<br>Education: NR | **Decision making: NR**<br>**CV risk factors and CVDs: NR**<br>• Smoking cessation after 6 months: NS<br>• Smoking amount reduced after 6 months: NS<br>**CV health behaviors**<br>• Medication prescribed within 1 month: NS<br>• Medication prescribed within 6 months: NS |
| Maindal<br>2014<br>Denmark | **Patients' Characteristics**<br>Age (Mean): I: 62, C: 62<br>Females: T: 47%, I: 152 (47.2), C1: 86 (46.0), C2: 59 (48.0)<br>Race/Ethnicity: NR<br>Education: At Least Vocational Education: I: 207 (68.5) , C1: 118 (66.3), C2: 90 (76.3) | **Decision making**<br>• Patient activation: 5.3, p = 0.017<br>• Perception of received care: NS<br>**CV risk factors and CVDs**<br>• Total cholesterol: -0.24mmol/l, p = 0.027<br>• Modelled cardiovascular risk at 3 years: NS<br>**CV health behaviors: NR** |
| Mathers<br>2012<br>United Kingdom | **Patients' Characteristics**<br>Age (Mean): I: 66 (39-82), C: 62 (42-87)<br>Males: I: 50 (52%), C: 46 (57%)<br>Race/Ethnicity:<br>White: I: 85 (89.5%), C: 71 (88.8%)<br>Education: Duration Of Education (Years) (Sd): I: 12.22 (4.83), C: 1.49 (2.74) | **Decision making**<br>• Decisional Conflict: 17.4 vs 25.2, p<0.001<br>• Better knowledge: 51.6% vs 28.8%, p<0.001<br>• Better realistic expectations:<br>- Risk of hypo: 81.0% vs 5.2%, p<0.001<br>- Risk of weight gain: 70.5% vs 5.3%, p<0.001<br>- Risk of complications: 26.3% vs 5.0% respectively, p<0.001<br>• Autonomous in decision-making: 64.1% vs 42.9%, p=0.012<br>**CV risk factors and CVDs** |

© 2024 Elias S et al. *JAMA Network Open.*

| Author Year Country | Patients' and clinician' sociodemographic Characteristics | Outcomes*<br>SDM and Decision making aspects<br>CV risk factors and CVDs<br>CV health behaviors |
|---|---|---|
| | | • Glycemic control: NS<br>**CV health behaviors: NR** |
| Moin<br>2019<br>United States | **Patients' Characteristics**<br>Age (Mean): I: 55.9 (11.5), C: 56.3 (11.6)<br>Females: I: 58.70%, C: 66.80%<br>Race/Ethnicity:<br>White: I: 52.20%, C: 52.60%<br>Black: I: 13.10%, C: 16.20%<br>Latino: I: 15.20%, C: 14.40%<br>Asian: I: 21.50%, C: 20.20%<br>Education: NR | **Decision making: NR**<br>**CV risk factors and CVDs**<br>• At 12-month adjusted weight loss: − 5.1 lbs, p < 0.001<br>**CV health behaviors**<br>• Use metformin: 19% vs. 1.6%, p < 0.001<br>• Uptake of DPP and/or metformin: 38% vs. 2%, p < 0.001 |
| Montgomery<br>2003<br>United Kingdom | **Patients' Characteristics**<br>Age (Mean): I: 57 (11), C1: 59 (9), C2: 60 (10), C3: 58 (11)<br>Females: I: 49%, C1: 46%, C2: 47%, C3: 49%<br>Race/Ethnicity: NR<br>Education: NR | **Decision making**<br>• Decisional conflict (decision analysis): 27.6 vs 38.9, p <0.001<br>Decisional conflict (video/leaflet): 30.3 vs 36.8, p = 0.021<br>• Knowledge (decision analysis): 73% vs 67%, p = 0.003<br>Knowledge (video/leaflet): 75% vs 65%, p <0.001<br>• Decision quality (decision analysis and video/leaflet): NS<br>• Increased state anxiety (decision analysis and video/leaflet): NS<br>**CV risk factors and CVDs: NR**<br>**CV health behaviors**<br>• Intention to start treatment: NS<br>• Proportion of patients prescribed antihypertensive medication: NS |
| Mullan<br>2009<br>United States | **Patients' Characteristics**<br>Age (Mean): I: 62.1 (10.9), C: 62.2 (12.4)<br>Females: I: 22 (46), C: 18 (49)<br>Race/Ethnicity: NR<br>Education: High School Education Completed: I: 46 (96), C: 34 (94)<br><br>**Clinician' Characteristics**<br>Age (Mean): NR<br>Males: NR<br>Race/Ethnicity: NR<br>Experience (Mean Years): NR<br>Professions: Nurse Practitioner: I: 3 (14), C: 1 (5); Physician: I: 17 (81), C: 13 (68); Residents: I: 1(5), C: 5 (26) | **Decision making**<br>• Involvement in making decisions: 21.8, 95% CI, 13.0-30.5<br>• Improved knowledge: 1.10, 95% CI, 0.11, 2.09<br>• Decisional Conflict: NS<br>• Trust in Physician: NS<br>• Clarity of information: NS<br>• Helpfulness of the information: NS<br>**CV risk factors and CVDs**<br>• HbA1c (at 6 months): NS<br>**CV health behaviors**<br>• Worse adherence (at 6 months): −8.88, 95%CI: −13.6 to −4.14<br>• Did not miss a dose in last week (at 6 months): 0.74, 95%CI: 0.24 to 2.32 |
| Naik<br>2011<br>United States | **Patients' Characteristics**<br>Age (Mean): I: 63.82 (7.9), C: 63.45 (7.8)<br>Males: I: NR C: NR<br>Race/Ethnicity: | **Decision making**<br>• Self-efficacy: 0.84, p=0.02<br>**CV risk factors and CVDs**<br>• HbA1c: difference 0.67%, p=0.03<br>**CV health behaviors: NR** |

© 2024 Elias S et al. *JAMA Network Open.*

| Author Year Country | Patients' and clinician' sociodemographic Characteristics | Outcomes*<br>SDM and Decision making aspects<br>CV risk factors and CVDs<br>CV health behaviors |
|---|---|---|
| | Black I: 15 (33.3), C: 12 (28.6)<br>Education: At Least Some College Education: I: 31 (69), C: 31 (74) | |
| O'Malley<br>2022<br>United States | **Patients' Characteristics**<br>Age (Mean): I: 65.6, C: 66.5<br>Females: I: 52.30%, C: 54.10%<br>Race/Ethnicity:<br>White: I: 43.8, C: 29.5<br>Black: I: 50, C: 62.3<br>Latino: I: 2.1, C: 3.3<br>Education: NR<br><br>**Clinician' Characteristics**<br>Age (Mean): 47<br>Females: 6 (54%)<br>Race/Ethnicity:<br>White: 5 (45%)<br>Experience (Mean Years): NR<br>Professions: Physicians: 11 (100%) | **Decision making**<br>• Shared decision making: NS<br>• Patient activation: 4.4 vs. 3.8, p = 0.047<br>• Patient satisfaction: NS<br>• Patient trust: NS<br>• Physician perceived difficulty of the encounter: NS<br>**CV risk factors and CVDs**<br>• Blood pressure change: NS<br>• Blood pressure control: NS<br>**CV health behaviors**<br>• Medication adherence: NS<br>• Change in adherence over time: NS |
| Peiris<br>2015<br>Autralia | **Patients' Characteristics**<br>Age (Mean): I: 60.7 (12.4), C: 61.3 (12.7)<br>Males: I: 7729 (40%), C: 8536 (44%)<br>Race/Ethnicity: Aboriginal/Torres Strait Islander: T: 19385, I: 3624 (18.7), C: 3292 (17.0)<br>Education: NR | **Decision making: NR**<br>**CV risk factors and CVDs**<br>• Systolic BP: NS<br>• Low-density lipo-protein cholesterol: NS<br>• Total & HDL cholesterol: Risk ratio: 1.19, p = 0.02<br>• **CV health behaviors**<br>• Recommended prescriptions: NS<br>• Treatment escalations for antiplatelet: 17.9% vs 2.7%; p<0.001<br>• Treatment escalations for lipid-lowering: 19.2% vs 4.8%; p<0.001<br>• Treatment escalations for blood pressure–lowering medications: 23.3% vs 12.1%; p=0.02<br>**Additional outcome**<br>• Appropriate screening for CVD risk: Risk ratio: 1.25, p=0.02 |
| Perestelo-Pérez<br>2016<br>Spain | **Patients' Characteristics**<br>Age (Mean): I: 63.9 (9.7), C: 59.6 (12.3)<br>Females: I: 35 (41), C: 28 (34)<br>Race/Ethnicity: NR<br>Education: Primary: I: 63(74.0%), C: 63(78.8%); Secondary: I: 16(18.8%), C: 12(15%); University: I: 6(7.1%), C: 5(6.3%) | **Decision making**<br>• Decisional conflict: NS<br>• Knowledge: 1.11, p = 0.01<br>• Satisfaction with decision-making process: 10.62, p = 0.01<br>• Consultation time: NS<br>• Perception of the 10-year risk of myocardial infarction<br>- without statins: 3.14, p = 0.01<br>- with statins: 2.47, p = 0.08 |

© 2024 Elias S et al. *JAMA Network Open.*

| Author Year Country | Patients' and clinician' sociodemographic Characteristics | Outcomes*<br>SDM and Decision making aspects<br>CV risk factors and CVDs<br>CV health behaviors |
|---|---|---|
| | | **CV risk factors and CVDs: NR**<br>**CV health behaviors**<br>• Adherence: NS<br>• Taking all pills during the last week: NS |
| Prabhakaran<br>2019<br>India | **Patients' Characteristics**<br>Age (Mean): I: 55.8(11.0), C: 54.5 (10.9)<br>Males: I: 1056 (57.3), C: 985 (53.1)<br>Race/Ethnicity: NR<br>Education: Illiterate, N (%): I: 772 (41.9), C: 635 (34.5); Primary Education, N (%): I: 331 (18.0), C: 374 (20.3); Secondary Education And Above, N (%): I: 739 (40.1), C: 847 (45.6) | **Decision making: NR**<br>**CV risk factors and CVDs**<br>• Systolic BP: NS<br>• HbA1c: NS<br>• Total cholesterol: NS<br>• Change in tobacco use: NS<br>• Body mass index: NS<br>**CV health behaviors: NR** |
| Ramallo-Fariña<br>2021<br>Spain | **Patients' Characteristics**<br>Mean Age: T 55.7 (7.1)<br>Female: 51.9%<br>Race/Ethnicity: NR<br>Education: NR<br>Employed: NR | **Decision making**<br>• Knowledge (at 12 and 24 months, PTI): p<0.007<br>Knowledge (at 24 months, PFI): p=0.01<br>Knowledge (at 12 months, CBI): p=0.008<br>• Self-empowerment (at 24 months, PTI): p=0.002<br>Self-empowerment (at 12 months, PFI): p<0.001<br>Self-empowerment (at 12 and 24months, CBI): p<0.008<br>• Distress (at 24 months, PTI): p=0.01<br>Distress (at 24 months, PFI): p=0.03<br>Distress (at 24 months, CBI): p=0.01<br>**CV risk factors and CVDs: NR**<br>• Proportion of patients who quit smoking<br>PTI at 12 and 24 months: p < 0.012<br>PFI: NS<br>CBI at 24 months: p=0.012<br>**CV health behaviors**<br>• Adherence to dietary recommendations PTI: at 12 and 24 months: p<0.01<br>PFI at 12 months: p<0.001<br>CBI at 24 months: p=0.004<br>• Medication adherence (PTI, PFI, CBI): NS |
| Rost<br>1991<br>United States | **Patients' Characteristics**<br>Mean Age: 40<br>Female: 60%<br>Education: 13.2 Y<br>Employed: 52.4% | **Decision making: NR**<br>• Asked for questions: 7.4 vs 3.0, p<0.001<br>**CV risk factors and CVDs:**<br>• Glycosylated hemoglobin levels at follow-up: NS<br>**CV health behaviors: NR** |

© 2024 Elias S et al. *JAMA Network Open.*

| Author Year Country | Patients' and clinician' sociodemographic Characteristics | Outcomes* SDM and Decision making aspects CV risk factors and CVDs CV health behaviors |
|---|---|---|
| | | **Additional outcomes:**<br>• Fewer physical limitations in activities of daily living at 4-month follow-up, $p = 0.02$<br>• Metabolic control ↑ only for intervention patients , $p = 0.02$ |
| Smith<br>2008<br>United States | **Patients' Characteristics**<br>Mean Age: 65<br>Female: 53%<br>Race: NR<br>Education: NR<br><br>**Clinician' Characteristics**<br>Physicians: 100%<br>Male: 64%<br>Median Years In Practice: 13-15 | **Decision making: NR**<br>**CV risk factors and CVDs**<br>• HbA1c: NS<br>• LDL: NS<br>• SBP: NS<br>• Estimated risk of coronary artery: NS<br>• Not smoking or advised to quit: OR 1.80, p=0.04<br>**CV health behaviors: NR** |
| Sperl-Hillen<br>2018<br>United States | **Patients' Characteristics**<br>Mean Age: 59<br>Female: 26%<br>White: 79%<br>Black: 12%<br>Education: NR | **Decision making: NR**<br>**CV risk factors and CVDs:**<br>• Better predicted annual rate of change in absolute 10-year CV risk, -2.24%, $p < 0.001$<br>• 10-year CV risk at 12 months post-index: 22.5% vs 24.4%, $p < 0.03$<br>**CV health behaviors: NR** |
| Swoboda<br>2017<br>United States | **Patients' Characteristics**<br>Mean Age: 56.76 (I); 55.41 (C)<br>Female: 67.57% (I); 70.59% (C)<br>White: 78.38% (I); 70.59% (C)<br>Black: 21.62% (I); 17.65% (C)<br>High School Diploma: 10.81% (I); 5.88% (C)<br>Full-Time Employed: 56.76% (I); 52.94% (C) | **Decision making:**<br>• Diabetes empowerment at week 16, 0.26 vs -0.27, p = 0.045<br>• Diabetes self-efficacy: NS<br>• Diabetes distress: NS<br>**CV risk factors and CVDs: NR**<br>**CV health behaviors:**<br>• Diet quality: NS<br>• **Additional outcome**<br>• Depressive symptoms: NS |
| Tinsel<br>2013<br>Germany | **Patients' Characteristics**<br>Mean Age: 63.8 (I); 65.0 (C)<br>Male: 46.7% (I); 44.7% (C)<br>Race: NR<br>Education: NR<br>Employment Status: NR | **Decision making:**<br>• Perceived participation: NS<br>• Knowledge about hypertension: NS<br>**CV risk factors and CVDs:**<br>• SBP: NS<br>• DBP: NS<br>• Cardiovascular risk score: NS<br>**CV health behaviors:**<br>• Adherence: NS |
| Tinsel | **Patients' Characteristics** | **Decision making:** |

© 2024 Elias S et al. *JAMA Network Open.*

| Author Year Country | Patients' and clinician' sociodemographic Characteristics | Outcomes*<br>SDM and Decision making aspects<br>CV risk factors and CVDs<br>CV health behaviors |
|---|---|---|
| 2018<br>Germany | Mean Age: 65.1 (I); 59.3 (C)<br>Female: 52.4% (I); 60.0% (C)<br>Race: NR<br>9 Years Of Education: 47.6% (I); 44.4% (C) | • Patient activation scores: 3.30, *p* = 0.023<br>**CV risk factors and CVDs:**<br>• Cardiovascular risk score: NS<br>• Systolic blood pressure: NS<br>• Total cholesterol: NS<br>• HDL cholesterol: NS<br>• BMI: NS<br>**CV health behaviors: NR** |
| Tusa<br>2021<br>Finland | **Patients' Characteristics**<br>Age (Mean): I: 69, C: 69<br>Female: I: 54% , C: 50%<br>Race/Ethnicity: NR<br>Education: NR | **Decision making: NR**<br>**CV risk factors and CVDs: NS**<br>• BMI: NS<br>• Haemoglobin A1c: NS<br>• SBP: NS<br>• DBP: NS<br>**CV health behaviors: NR** |
| Tutino<br>2017<br>China | **Patients' Characteristics**<br>Age (Mean): I: 56.1, C:56.8<br>Male: I: 54.4%, C: 54.5%<br>Race/Ethnicity: NR<br>Education: NR | **Decision making: NR**<br>**CV risk factors and CVDs:**<br>• HbA1c: NS<br>• SBP: NS<br>• LDL cholesterol: NS<br>• Blood glucose defaulters: 25.6% vs. 32.0%, p < 0001<br>**CV health behaviors:**<br>• Blood glucose self-monitoring; 50.5% vs. 44.2%, p = 0.005<br>• Physical activity: NS<br>• Adherence to balanced diet: NS |
| van Steenkiste<br>2007<br>Netherlands | **Patients' Characteristics**<br>Mean Age: 54 (I); 54 (C)<br>Male: 45% (I); 45% (C)<br>Race: NR<br>Education: NR<br><br>**Clinician' Characteristics**<br>Mean Age: 49 (I); 47 (C)<br>Male: 38% (I); 35% (C)<br>Mean Work Experience: 19 (I); 16 (C)<br>Full-Time Employment: 38% (I); 21% (C) | **Decision making**<br>• Provider's performance: NS<br>• Patients' risk perception: NS<br>**CV risk factors and CVDs: NR**<br>**CV health behaviors:**<br>• Physical activity in men: OR 3.8, p < 0.05 |
| Warner<br>2015<br>United States | **Patients' Characteristics**<br>Age (Mean): I: 54, C: 53<br>Female: I: 44% , C: 57% | **Decision making:**<br>• Improved measures of decisional quality: 86 vs 76, p= 0.0003<br>• Patient involvement in decision making: 46 vs 23, p< 0.0001 |

© 2024 Elias S et al. *JAMA Network Open.*

| Author Year Country | Patients' and clinician' sociodemographic Characteristics | Outcomes*<br>SDM and Decision making aspects<br>CV risk factors and CVDs<br>CV health behaviors |
|---|---|---|
| | Race/Ethnicity:<br>White: I: 86% , C:95% (C)<br>Education: Some College: I: 65%, C: 62% | • Clinician decisional comfort: 81 vs 74, p=0.034<br>• Smoking knowledge: NS<br>**CV risk factors and CVDs: NR**<br>**CV health behaviors: NS** |
| Weymiller<br>2007<br>United States | **Patients' Characteristics**<br>Age (Mean): I: 64, C:66<br>Female: I: 31%, C: 57%<br>Race/Ethnicity: NR<br>Education:<br>High School Completed: I: 98%, C: 87% | **Decision making:**<br>• Patients favored using the decision aid: OR 2.8, 95% CI, 1.2-6.9<br>• Knowledge: 2.4, 95% CI, 1.5-3.3<br>• Decisional conflict: −10.6, 95% CI, −15.4 to −5.9<br>**CV risk factors and CVDs:**<br>• Estimated cardiovascular risk: OR 22.4, 95% CI, 5.9- 85.6<br>• Potential absolute risk reduction with statin drugs: OR 6.7, 95% CI, 2.2-19.7<br>**CV health behaviors:**<br>• Missing medication dose: OR 3.4, 95% CI, 1.5-7.5 |
| Wollny<br>2019<br>Germany | **Patients' Characteristics**<br>Age (Mean): I: 65.9, C: 65.8<br>Female: I: 44.6%, C: 46.7%<br>Race/Ethnicity: NR<br>Education: NR | **Decision making: NS**<br>Shared decision making: NS<br>Patient-centeredness: NS<br>**CV risk factors and CVDs: NR**<br>**CV health behaviors: NR** |
| Yu<br>2020<br>Canada | **Patients' Characteristics**<br>Age (Mean): 65.9 (I); 65.8 (C)<br>Female: 54.9% (I); 41.4% (C)<br>White: 63.3% (I); 67.6% (C)<br>Race/Ethnicity: NR<br>Education: Bachelor's Degree: 23.3% (I); 16.0% (C)<br><br>**Clinician' Characteristics**<br>Female: 72% (I); 46% (C) | **Decision making**<br>• Reduction in total decisional conflicts: NS<br>• Diabetes distress: NS<br>• Patient assessment of chronic illness care: 0.7, p<0.001<br>**CV risk factors and CVDs: NR**<br>**CV health behaviors: NR**<br>**Additional outcome**<br>• Health-related quality of life: NS |

*Outcomes reported as total difference, or intervention versus control and P value.
NR: Not Reported; NS: Non-significant. I: Intervention Group; C: Control Group; T: Total groups; BP: Blood Pressure; RCTs: Randomized Controlled Trials, OR: Odds Ratio; CVD: Cardiovascular disease

© 2024 Elias S et al. *JAMA Network Open.*

**eTable 3.** Summary of Interventions

| Author, Year, Country | Country | Target | Main Topic | Frequency (F) and duration (D) | Patients' intervention | | | | | | | | | | Clinician' intervention/ training | | | | Decision aid used |
|---|---|---|---|---|---|---|---|---|---|---|---|---|---|---|---|---|---|---|---|
| | | | | | Individual consultation | Group session | Workbook, card, diary, printed materials, etc | Text message | Email | Software/ app | Website | Phone | Video | Behavior change techniques (including motivational | SDM skills | Communication or education skills, motivational interviewing | Behavior change techniques | Training in the use of decision aid | |
| Adarkwah 2016, Germany | Germany | Patients and clinician | multiple CVD risk factors | F: Once in 3 months<br>D: NR | X | | | | | X | X | | | | | X | | X | Arriba software |
| Applegate 2021 United States | United States | Patients | multiple CVD risk factors | F: 4 monthly visits followed by 2 quarterly visits<br>D: 40 min | X | | X | | | | | X | | X | | | X | | None |
| Bailey 2016 United States | United States | Patients | diabetes | F: Once<br>D: 30 min | | | | X | X | | X | | X | | | | | X | Diabetes decision Aid for type 2 diabetes mellitus |
| Bailey 2018, United States | United States | Patients | diabetes | F: Once<br>D: NR | X | | | | | | X | | | | | | | X | Diabetes Decision Aid for type 2 diabetes mellitus |
| Boulware 2020, United States | United States | Patients | hypertension | F: Once<br>D: NR | X | | X | | | | | X | | | | | | | None |
| Branda 2013, United States | United States | Patients and clinician | diabetes | F: Once<br>D: NR | X | | | | | | | | | | | | | X | DA for diabetes medication choice, and statin choice |
| Buhse 2015, Germany | Germany | Patients and clinician | diabetes | F: Once<br>D: 90 min | X | | | | | | X | | X | | X | X | | X | Evidence-based decision aid for patients on the |

© 2024 Elias S et al. *JAMA Network Open.*

| Author, Year, Country | Country | Target | Main Topic | Frequency (F) and duration (D) | Patients' intervention | | | | | | | | | | Clinician' intervention/ training | | | | Decision aid used |
|---|---|---|---|---|---|---|---|---|---|---|---|---|---|---|---|---|---|---|---|
| | | | | | Individual consultation | Group session | Workbook, card, diary, printed materials, etc | Text message | Email | Software/ app | Website | Phone | Video | Behavior change techniques (including motivational | SDM skills | Communication or education skills, motivational interviewing | Behavior change techniques | Training in the use of decision aid | |
| | | | | | | | | | | | | | | | | | | | prevention of heart attack |
| Buhse 2018, Germany | Germany | Patients and clinician | diabetes | F: NR<br>D: NR | X | | X | | | | | | | | X | | | X | An evidence-based patient decision aid |
| Cheng 2021, China | China | Patients | diabetes | F: Weekly intervention<br>D: 6 weeks | X | X | | | | | | X | | | | | | | None |
| Cooper 2011, United States | United States | Patients and clinician | hypertension | F: Bimonthly and monthly<br>D: NR | | | X | | | | | | | | X | | | | None |
| Coronado-Vázquez 2019, Spain | Spain | Patients and clinician | multiple CVD risk factors and medication | F: One time visit to doctor's office<br>D: NR | X | | | | | | X | X | X | | | | | X | A decision support tool in paper format |
| Den Ouden 2017, Netherlands | Netherlands | Patients and clinician | multiple CVD risk factors | F: 2 times 12 months apart<br>D: NR | X | | | | | | | | | | X | | | X | OPTIMAL decision support aid |
| Denig 2014, Netherlands | Netherlands | Patients | diabetes | F: Once<br>D: NR | X | | X | | | | | | | | | X | | X | A decision aid for people with diabetes |
| Dwinger 2020, Germany | Germany | Patients | multiple CVD risk | F: Every six weeks<br>D: 1 year | | | | | | | | X | | X | | | | | None |

© 2024 Elias S et al. *JAMA Network Open.*

| Author, Year, Country | Country | Target | Main Topic | Frequency (F) and duration (D) | Patients’ intervention | | | | | | | | | | Clinician’ intervention/ training | | | | Decision aid used |
|---|---|---|---|---|---|---|---|---|---|---|---|---|---|---|---|---|---|---|---|
| | | | | | Individual consultation | Group session | Workbook, card, diary, printed materials, etc | Text message | Email | Software/ app | Website | Phone | Video | Behavior change techniques (including motivational | SDM skills | Communication or education skills, motivational interviewing | Behavior change techniques | Training in the use of decision aid | |
| | | | factors | | | | | | | | | | | | | | | | |
| Eaton 2011, United States | United States | Patients and clinician | multiple CVD risk factors | F: NR<br>D: 1 hour | X | | X | | | | X | | X | | | X | | X | A personal digital assistant (PDA)-based decision support tool for physician |
| Eckman 2012, United States | United States | Patients | multiple CVD risk factors | F: NR<br>D: 70 min | X | | X | | | | | | X | | | | | | None |
| Edwards 2006, United Kindom | United Kingdom | Patients | diabetes | F: Once<br>D: NR | | | | | | | | | X | | | | | | None |
| Farmer 2005, United Kingdom | United Kingdom | Patients | diabetes | F: 3 times over 9 months<br>D: NR | X | | X | | | | X | | X | | | | | | None |
| Grant 2008, United States | United States | Patients | diabetes | F: NR<br>D: NR | | | | | | | X | | X | | | | | | None |
| Greenfield 1988, United States | United States | Patients | diabetes | F: Twice<br>D: 20 min | X | | | | | | | | | | | | | | None |
| Heisler 2014, United States | United States | Patients | diabetes | F: 4 times during 6 weeks<br>D: 1.5-2 hours | X | | X | | | | X | X | X | | | X | | | None |

© 2024 Elias S et al. *JAMA Network Open.*

| Author, Year, Country | Country | Target | Main Topic | Frequency (F) and duration (D) | Patients' intervention | | | | | | | | | | Clinician' intervention/ training | | | | Decision aid used |
|---|---|---|---|---|---|---|---|---|---|---|---|---|---|---|---|---|---|---|---|
| | | | | | Individual consultation | Group session | Workbook, card, diary, printed materials, etc | Text message | Email | Software/ app | Website | Phone | Video | Behavior change techniques (including motivational | SDM skills | Communication or education skills, motivational interviewing | Behavior change techniques | Training in the use of decision aid | |
| Hsu 2016, United States | United States | Patients | diabetes | F: Weekly<br>D: 12 weeks | X | | | X | | X | | | X | | | | | | None |
| Hu 2021, China | China | Patients | diabetes and medication | F: 5 times over 12 weeks<br>D: NR | X | | | | | | | | | | | | | | None |
| Jaspers 2021, Netherlands | Netherlands | Patients | CVD risk | F: Once<br>D: NR | X | | X | | | | | X | X | | | | | | None |
| Jouni 2017, United States | United States | Patients | multiple CVD risk factors | F: NR<br>D: NR | X | | | | | | X | | | | | | | X | A modified version of the Statin Choice decision aid |
| Karagiannis 2016, Greece | Greece | Patients | diabetes | F: initial visit, 12 weeks, and 24 weeks<br>D: 6 months | X | | X | | | | X | | | | | | | X | Greek version of the Diabetes Medication Choice Decision Aid |
| Kask-Flight 2021, Estonia | Estonia | Patients and clinician | multiple CVD risk factors | F: NR<br>D: 3 months | X | | | | | | X | | | | | | | X | A computer-based DA program, ARRIBA HERZ |
| Keyserling 2014, United States | United States | Patients | smoking | F: 7 times<br>D: 45-60 min followed by 15-30 min | X | | | | | | X | | X | | | | | X | A decision aid for CVD |

© 2024 Elias S et al. *JAMA Network Open.*

| Author, Year, Country | Country | Target | Main Topic | Frequency (F) and duration (D) | Patients' intervention | | | | | | | | | | Clinician' intervention/ training | | | | Decision aid used |
|---|---|---|---|---|---|---|---|---|---|---|---|---|---|---|---|---|---|---|---|
| | | | | | Individual consultation | Group session | Workbook, card, diary, printed materials, etc | Text message | Email | Software/ app | Website | Phone | Video | Behavior change techniques (including motivational | SDM skills | Communication or education skills, motivational interviewing | Behavior change techniques | Training in the use of decision aid | |
| Koelewijn-Van Loon 2009, Netherlands | Netherlands | Patients and clinician | multiple CVD risk factors | F: Twice<br>D: 15-20 min | X | | X | | | | X | X | | | | | | X | A decision aid for CVD |
| Koelewijn-Van Loon 2010, Netherlands | Netherlands | Patients and clinician | multiple CVD risk factors | F: Twice<br>D: 20 min | X | | | | | | | X | | | | X | | | None |
| Krones 2008, Germany | Germany | Patients and clinician | CVD risk | F: NR<br>D: NR | X | | | | | | | | | | | X | | X | A decision aid for CVD |
| Kulzer 2018, Germany | Germany | Patients and clinician | diabetes and medication | F: 6 times<br>D: NR | X | | | | | | | | | | X | | | | None |
| Kunneman 2022, United States | United States | Patients and clinician | diabetes and medication | F: Once<br>D: NR | X | | X | | | | | | | | | | | X | A Diabetes Medication Choice conversation aid |
| Lauffenburger 2019, United States | United States | Patients | diabetes and medication | F: Four times<br>D: 30 min | | | X | | | | | X | | | X | | | X | A postcard sized SDM tool |
| Lee 2016, South Korea | South Korea | Patients | smoking | F: Once<br>D: 7-minute video & 5-15 minutes of routine medical care & 5-10 minutes of smoking cessation counseling | X | | | | | | X | | X | | | | | X | A decision aid |

© 2024 Elias S et al. *JAMA Network Open.*

| Author, Year, Country | Country | Target | Main Topic | Frequency (F) and duration (D) | Patients' intervention | | | | | | | | | | Clinician' intervention/ training | | | | Decision aid used |
|---|---|---|---|---|---|---|---|---|---|---|---|---|---|---|---|---|---|---|---|
| | | | | | Individual consultation | Group session | Workbook, card, diary, printed materials, etc | Text message | Email | Software/ app | Website | Phone | Video | Behavior change techniques (including motivational | SDM skills | Communication or education skills, motivational interviewing | Behavior change techniques | Training in the use of decision aid | |
| | | | | and prescription | | | | | | | | | | | | | | | |
| Maindal 2014, Denmark | Denmark | Patients and clinician | diabetes | F: 2 interviews and 8 group session D: 18 h over 3 months | X | X | | | | | | | | | | | X | | None |
| Mathers 2012, United Kingdom | United Kingdom | Patients and clinician | diabetes | F: NR D: NR | X | | | | | | | | | | X | X | | X | A PANDAs decision aid |
| Moin 2019, United States | United States | Patients | diabetes and medication | F: NR D: 35-45 min | X | | X | | | | | | | | X | | | X | A decision aid for diabetes prevention |
| Montgomery 2003, England, United Kindom | United Kingdom | Patients | hypertension and medication | F: NR D: 60 min | X | | X | | | X | X | | X | | | | | X | A simple decision tree |
| Mullan 2009, United States | United States | Patients | diabetes and medication | F: Once D: 3 min | X | | X | | | | | | | | | | | X | The Diabetes Medication Choice decision aid tool |
| Naik 2011, United States | United States | Patients | diabetes | F: Every 3 weeks D: 3 months, 1 | X | X | | | | | | | | | | | | | None |

© 2024 Elias S et al. *JAMA Network Open.*

| Author, Year, Country | Country | Target | Main Topic | Frequency (F) and duration (D) | Patients' intervention | | | | | | | | | | Clinician' intervention/ training | | | | Decision aid used |
|---|---|---|---|---|---|---|---|---|---|---|---|---|---|---|---|---|---|---|---|
| | | | | | Individual consultation | Group session | Workbook, card, diary, printed materials, etc | Text message | Email | Software/ app | Website | Phone | Video | Behavior change techniques (including motivational | SDM skills | Communication or education skills, motivational interviewing | Behavior change techniques | Training in the use of decision aid | |
| | | | | hour/each session | | | | | | | | | | | | | | | |
| O'malley 2022, United States | United States | Patients and clinician | multiple CVD risk factors and medication | F: Once<br>D: NR | | | X | | | | | | | | X | | | | None |
| Peiris 2015, Australia | Australia | Patients and clinician | CVD risk | F: Monthly<br>D: 48 min | | | | | | X | | | | | | | | | None |
| Perestelo-Pérez 2016, Spain | Spain | Patients and clinician | CVD risk and medication | F: Once<br>D: 1 hour | X | | X | | | | | | | | | | | X | A decision aid for CVD |
| Prabhakaran 2019, India | India | Patients and clinician | multiple CVD risk factors | F: NR<br>D: 12 months | | | X | X | | X | | | | | | | | X | mWellcare system |
| Ramallo-Fariña 2021, Spain | Spain | Patients and clinician | diabetes | F: Every 3 months<br>D: 2 years | X | X | X | X | | | X | | | | X | | | X | A decision-making aid for diabetes prevention |
| Rost 1991, United States | United States | Patients | diabetes | F: 2<br>D: 45 min + 1hr | X | | X | | | | | | | | | | | X | Decision tree |
| Smith 2008, United States | United States | Patients and clinician | diabetes | F: NR<br>D: NR | | | | | | | | | X | | | | | X | Electronic decision support system |

© 2024 Elias S et al. *JAMA Network Open.*

| Author, Year, Country | Country | Target | Main Topic | Frequency (F) and duration (D) | Patients' intervention | | | | | | | | | | Clinician' intervention/ training | | | | Decision aid used |
|---|---|---|---|---|---|---|---|---|---|---|---|---|---|---|---|---|---|---|---|
| | | | | | Individual consultation | Group session | Workbook, card, diary, printed materials, etc | Text message | Email | Software/ app | Website | Phone | Video | Behavior change techniques (including motivational | SDM skills | Communication or education skills, motivational interviewing | Behavior change techniques | Training in the use of decision aid | |
| Sperl-Hillen, 2018 United States | United States | Patients and clinician | diabetes | F: NR<br>D: NR | | | | | | | X | | | | | | | X | EHR-integrated, point-of-care clinical decision support system |
| Swoboda 2017, United States | United States | Patients | diabetes | F: one in-person session and bi-weekly phone call till week 16<br>D: NR | X | | | | | | | X | | X | | | | | None |
| Tinsel 2013, Germany | Germany | Clinician | hypertension | F: Once<br>D: NR | | | | | | | | | | | X | X | | | None |
| Tinsel 2018, Germany | Germany | Patients | CVD risk | F: 4 consultations<br>D: NR | X | | X | | | | | | | | | | | X | DECADE brochure |
| Tusa 2021, Finland | Finland | Patients | CVD risk | F: One health care visit<br>D: 30-60 minutes with nurse and 30-40 minutes with general practitioner | X | | | | | | | | | | | | | | None |
| Tutino 2017, China | China | Patients and clinician | diabetes | F: One intervention session and at least 2 follow-up facilitated by a nurse coordinator<br>D: 2-4 hours of | X | | | | | | X | | | | | | | X | Web-based disease management program |

© 2024 Elias S et al. *JAMA Network Open.*

| Author, Year, Country | Country | Target | Main Topic | Frequency (F) and duration (D) | Patients' intervention | | | | | | | | | | Clinician' intervention/ training | | | | Decision aid used |
|---|---|---|---|---|---|---|---|---|---|---|---|---|---|---|---|---|---|---|---|
| | | | | | Individual consultation | Group session | Workbook, card, diary, printed materials, etc | Text message | Email | Software/ app | Website | Phone | Video | Behavior change techniques (including motivational | SDM skills | Communication or education skills, motivational interviewing | Behavior change techniques | Training in the use of decision aid | |
| | | | | diabetes education | | | | | | | | | | | | | | | |
| van Steenkiste 2007, Netherlands | Netherlands | Patients and clinician | multiple CVD risk factors | F: One training and two consultations D: 8 months | X | | X | | | | | | | | | | X | X | Decision support tool |
| Warner 2015, United States | United States | Patients and clinician | smoking | F: Once D: NR | X | | X | | | | | | | | | | | X | Decision aid |
| Weymiller 2007, United States | United States | Patients and clinician | diabetes | F: Once and 3-month follow-up D: NR | X | | X | | | | | | | | | | | X | Statin Choice decision aid |
| Wollny 2019, Germany | Germany | Patients and clinician | diabetes | F: Once D: NR | X | | | | | | | | | | X | X | | X | Electronic decision aid |
| Yu 2020, Canada | Canada | Patients and clinician | diabetes | F: NR D: NR | X | | | | | | | | X | | X | X | | X | Web-based patient decision aid |

**X: Reported in this study. NR: not reported. F: Frequency. D: duration. SMD: shared decision-making. CVD: cardiovascular disease.**

© 2024 Elias S et al. *JAMA Network Open.*

**eTable 4.** Studies Incorporating Decision Aids in the Intervention

| Author, Year, Country | Target and main topic | Decision aid | Format |
|---|---|---|---|
| Adarkwah 2016, Germany | Patients and clinician, multiple CVD risk factors | Arriba software | Software application |
| Bailey 2016, United States | Patients only, diabetes | Diabetes decision Aid for type 2 diabetes mellitus | Online |
| Bailey 2018, United States | Patients only, diabetes | Diabetes Decision Aid for type 2 diabetes mellitus | Online |
| Branda 2013, United States | Patients and clinician, diabetes | Decision aid for diabetes medication choice, and statin choice | Online |
| Buhse 2015, Germany | Patients and clinician, diabetes | Evidence-based decision aid for patients on the prevention of heart attack | Online |
| Buhse 2018, Germany | Patients and clinician, diabetes | An evidence-based patient decision aid | Online |
| Coronado-Vázquez 2019, Spain | Patients and clinician, multiple CVD risk factors and medication | A decision support tool in paper format | Paper-based |
| Den Ouden 2017, Netherlands | Patients and clinician, multiple CVD risk factors | OPTIMAL decision support aid | Paper-based |
| Denig 2014, Netherlands | Patients only, diabetes | A decision aid for people with diabetes | Computer-based |
| Eaton 2011, United States | Patients and clinician, multiple CVD risk factors | A personal digital assistant -based decision support tool for physician | Personal digital assistant (PDA)-based |
| Jouni 2017, United States | Patients only, multiple CVD risk factors | A modified version of the Statin Choice decision aid | Online |
| Karagiannis 2016, Greece | Patients only, diabetes | Greek version of the Diabetes Medication Choice Decision Aid | Online |
| Kask-Flight 2021, Estonia | Patients and clinician, multiple CVD risk factors | A computer-based decision aid program, ARRIBA HERZ | Computer-based |
| Keyserling 2014, United States | Patients only, smoking | A decision aid for CVD | Online |
| Koelewijn-Van Loon 2009, Netherlands | Patients and clinician, multiple CVD risk factors | A decision aid for CVD | Booklet-based |
| Krones 2008, Germany | Patients and clinician, CVD risk | A decision aid for CVD | Online |
| Kunneman 2022, United States | Patients and clinician, diabetes and medication | A Diabetes Medication Choice conversation aid | Online |
| Lauffenburger 2019, United States | Patients only, diabetes and medication | A postcard sized SDM tool | Postcard-based |
| Lee 2016, | Patients only, smoking | A decision aid | Video-based |

© 2024 Elias S et al. *JAMA Network Open.*

| | | | |
|---|---|---|---|
| South Korea | | | |
| Mathers 2012,United Kingdom | Patients and clinician, diabetes | A PANDAs decision aid | Postcard-based |
| Moin 2019, United States | Patients only, diabetes and medication | A decision aid for diabetes prevention | Online |
| Montgomery 2003, England, United Kingdom | Patients only, hypertension and medication | A simple decision tree | Decision tree |
| Mullan 2009, United Sates | Patients only, diabetes and medication | The Diabetes Medication Choice decision aid tool | Postcard-based |
| Perestelo-Pérez 2016, Spain | Patients and clinician, CVD risk and medication | A decision aid for CVD | Online |
| Prabhakaran 2019, India | Patients and clinician, multiple CVD risk factors | mWellcare system | Software application |
| Ramallo-Fariña 2021, Spain | Patients and clinician, diabetes | A decision-making aid for diabetes prevention | Online |
| Rost 1991, United States | Patients only, diabetes | Decision tree | Decision tree |
| Smith 2008, United States | Patients and clinician, diabetes | Electronic decision support system | Online |
| Sperl-Hillen 2018, United States | Patients and clinician, cardiovascular risk | EHR-integrated, point-of-care clinical decision support system | Online |
| Tinsel 2018, Germany | Patients, CVD risk | DECADE Brochure | Brochure-based |
| Tutino 2017, China | Patients, diabetes | Web-based disease management program | Online |
| van Steenkiste 2007, Netherlands | Clinician and patients, multiple CVD risk factors | Decision support tool | Booklet-based |
| Warner 2015, United States | Clinician and patients, smoking | Decision aid | Postcard-based |
| Weymiller 2007, United States | Clinician and patients, diabetes | Decision aid | Online |
| Wollny 2019, Germany | Clinician and patients, diabetes | Electronic decision aid | Online |
| Yu 2020, Canada | Clinician and patients, diabetes | Web-based patient decision aid | Online |

CVD: cardiovascular disease; SDM: Shared decision making; GP: general practitioner; SBP: systolic blood pressure, DBP: diastolic blood pressure.

© 2024 Elias S et al. *JAMA Network Open.*

**eTable 5.** Summary of Outcomes

| Outcomes | Adarkwah 2016 | Applegate 2021 | Bailey 2016 | Bailey 2018 | Boulware 2020 | Branda 2013 | Buhse 2015 | Buhse 2018 | Cheng 2021 | Cooper 2011 | Coronado-Vázquez 2019 | Den Ouden 2017 | Denig 2014 | Dwinger 2020 | Eaton 2011 | Eckman 2012 | Edwards 2006 | Farmer 2005 | Grant 2008 | Greenfield 1988 | Heisler 2014 | Hsu 2016 | Hu 2021 | Jaspers 2021 | Jouni 2017 | Karagiannis 2016 | Kask-Flight 2021 | Keyserling 2014 | Koelewijn-Van Loon 2009 | Koelewijn-Van Loon 2010 |
|---|---|---|---|---|---|---|---|---|---|---|---|---|---|---|---|---|---|---|---|---|---|---|---|---|---|---|---|---|---|---|
| **Decisional outcomes** | | | | | | | | | | | | | | | | | | | | | | | | | | | | | | |
| SDM | + | | | | | | | | | | | | | | | | | | | | | | | NS | NS | | | | | |
| Patient centeredness | | | | | NS | + | | | | + | | | | | | | | | | | | | | | | | | | | |
| Decisional Conflict | + | | + | | | | | | | | | | | | | | NS | | | | NS | | | + | | NS | | | | |
| Decision quality | | | | | | | | + | | + | | | | | | | | | | + | | | | | NS | | | | | |
| Self-efficacy | | | + | NS | NS | | | | | | | | | | | | | | | | NS | | | | | | | | | |
| Empowerment | | | | | | | | | + | | | | NS | | | | | | | | | | + | | | | | | | |
| Patient activation | | | | | | | | | | | | | | + | | | | | | | | | | NS | | | | | | |
| Knowledge | | | + | | | + | + | + | | | | | | + | | NS | | | | NS | NS | | + | | | NS | | | | |
| Distress | | | | | | | | | + | | | | | | | | | | | | + | | | | | | | | | + |
| Satisfaction | | | | | | | | | | | | | | | | | NS | | | NS | NS | + | | | NS | NS | | | | + |
| Risk Perception & Realistic expectations | | | | | | | + | | | | | | | | | | | | | | | | | | | | | | | + |
| Behavior change commendations | | | | | NS | | | | | | | | | + | | | | | | + | | | | | | | | | | |
| Encounter length | | | | | | | | | | | | | | | | | | | | NS | | | | | | | | | | |
| **Cardiovascular risk factors outcomes** | | | | | | | | | | | | | | | | | | | | | | | | | | | | | | |
| Diabetes | | NS | | | | NS | + | | | | | NS | NS | | | | | NS | | + | NS | + | + | | | NS | | NS | | |
| Hypertension | | NS | | | NS | | NS | | | NS | | NS | NS | | | NS | | | NS | | | | | | | | + | NS | | |
| Dyslipidemia | | NS | | | | NS | | | | | | NS | | | NS | | | | NS | | | | | NS | | | NS | NS | | |
| Obesity | | NS | | | | | | | | | | NS | | + | | NS | | | | | | ns | NS | | | NS | NS | NS | | |
| Tobacco Use | | | | | | | + | | | | | | | + | | | | | | | | | | | | | + | NS | NS | + |
| CVD Risk | | | | | | | | | | | | | | | | | | | | | | | | | | | | + | NS | |
| **Cardiovascular health behaviors outcomes** | | | | | | | | | | | | | | | | | | | | | | | | | | | | | | |
| Physical Activity | | | | | | | | | | | | | | + | | NS | | | | | | | | | | | | NS | NS | NS |
| Diet | | | | | | | | | | | | | | | | | | | | | | | | | | | | NS | NS | NS |
| Self-management | | | | | NS | | | | | | | | | + | | | | | | NS | | | + | | | | | | | |
| Adherence | | | | | | NS | NS | NS | | NS | | | | | | NS | | | | | NS | | | | | NS | | NS | | |
| Medication management | | | | | | + | | | | | + | | NS | | | | | | + | + | | NS | | | | | | NS | | |

NS: non-significant. +: There was a statistically significant positive relationship or effect observed in the analysis. SDM: shared decision-making. CVD: cardiovascular disease.

© 2024 Elias S et al. *JAMA Network Open.*

**eTable 5.** Summary of Outcomes (continued)

| Outcomes | Krones 2008 | Kulzer 2018 | Kunneman 2022 | Lauffenburger 2019 | Lee 2016 | Maindal 2014 | Mathers 2012 | Moin 2019 | Montgomery 2003 | Mullan 2009 | Naik 2011 | O'malley 2022 | Peiris 2015 | Perestelo-Pérez 2016 | Prabhakaran 2019 | Ramallo-Fariña 2021 | Rost 1991 | Smith 2008 | Sperl-Hillen 2018 | Swoboda 2017 | Tinsel 2013 | Tinsel 2018 | Tusa 2021 | Tutino 2017 | van Steenkiste 2007 | Warner 2015 | Weymiller 2007 | Wollny 2019 | Yu 2020 |
|---|---|---|---|---|---|---|---|---|---|---|---|---|---|---|---|---|---|---|---|---|---|---|---|---|---|---|---|---|---|
| **Decisional outcomes** | | | | | | | | | | | | | | | | | | | | | | | | | | | | | |
| SDM | + | | | | | | | | | | | NS | | | | | | | | | | | | | | | | NS | |
| Patient centeredness | | | + | | | | | | | + | | | | | | | + | | | | | | | | | + | | NS | |
| Decisional Conflict | + | | NS | | | | + | | + | NS | | | | NS | | | | | | | | | | | | | + | | NS |
| Decision quality | | + | NS | | | | | | NS | NS | | | | | | | | | | | | | | | | + | | | |
| Self-efficacy | | | | | | | | | | | + | | | | | | | | | NS | | | | | | | | | |
| Empowerment | | | | | | | | | | | | | | | | + | | | | + | | | | | | | | | |
| Patient activation | | | | | | + | | | | | | + | | | | | | | | | | + | | | | | | | |
| Knowledge | NS | | + | | | | + | | + | + | | | | + | | + | | | | | NS | | | | | NS | + | | |
| Distress | | | | | | | | | NS | | | | | | | + | | | | NS | | | | | | | | | NS |
| Satisfaction | + | + | NS | | | | | | | | | NS | | + | | | | | | | | | | | NS | | | | + |
| Risk Perception & Realistic expectations | | | | | | | + | | | | | | | + | | | | | | | | | | | NS | | | | |
| Behavior change recommendations | | + | | | | | | | | | | | | | | | | | | | | | | | | | | | |
| Encounter length | | | NS | | | | | | | | | | | NS | | | | | | | | | | | | | | | |
| **Cardiovascular risk factors outcomes** | | | | | | | | | | | | | | | | | | | | | | | | | | | | | |
| Diabetes | | + | NS | NS | | | NS | | | NS | + | | | | NS | | NS | NS | | | | | NS | NS | | | | | |
| Hypertension | | | | | | | | | | | | NS | NS | | NS | | | NS | | | NS | NS | NS | NS | | | | | |
| Dyslipidemia | | | | | | + | | | | | | | + | | NS | | | NS | | | | NS | | NS | | | | | |
| Obesity | | | | | | | | + | | | | | | | NS | | | | | | | NS | NS | | | | | | |
| Tobacco Use | | | | | NS | | | | | | | | | | NS | + | | NS | | | | | | | | | | | |
| CVD Risk | NS | | | | | NS | | | | | | | | | | | | NS | + | | NS | NS | | | | | + | | |
| **Cardiovascular health behaviors outcomes** | | | | | | | | | | | | | | | | | | | | | | | | | | | | | |
| Physical Activity | | + | | | | | | | | | | | | | | | | | | | | | | NS | + | | | | |
| Diet | | + | | | | | | | | | | | | | | | | | | NS | | | | NS | | | | | |
| Self-management | | | | | | | | | | | | | | | | | | | | | | | | + | | | | | |
| Adherence | | + | NS | NS | | | | | NS | - | | NS | | NS | | + | | | | | NS | | | | | | + | | |
| Medication management | | + | | | NS | | | + | NS | | | | + | | | | | | | | | | | | | | | | |

NS: non-significant. +: There was a statistically significant positive relationship or effect observed in the analysis. SDM: shared decision-making. CVD: cardiovascular disease.

© 2024 Elias S et al. *JAMA Network Open.*

**eTable 6.** Studies Reporting Satisfaction Outcome

| Author, Year, Country | Country | Target | Main Topic | Measurements | Score range | Time measured | Effect size | CG: n | CG: mean | CG: SD | IG 1: n | IG : mean | IG: SD | P value |
|---|---|---|---|---|---|---|---|---|---|---|---|---|---|---|
| Edwards 2006, United Kindom | United Kingdom | Patients and family | diabetes | Four further questions addressed satisfaction with information and the value of the information to participants in making treatment decisions | NR | Immediately | NR | NR | NR | NR | NR | NR | NR | NR |
| Greenfield 1988, United States | United States | Patients | diabetes | 12-item patient satisfaction with the interpersonal style of the physician, the technical quality of care and care in general | 5-60 | Immediately | 0.2 (95% CI: -4.2 to 4.6) | 26 | 45.9 | 9.1 | 33 | 46.1 | 7.9 | NR |
| Heisler 2014, United States | United States | Patients | diabetes | Satisfaction with clarity of medication information | 0-100 | Immediately | NR | 95 | 88.4 | 16 | 92 | 89 | 17.5 | NR |
| Hsu 2016, United States | United States | Patients | diabetes | Diabetes Treatment Satisfaction Questionnaire | 0-48 | 2 weeks | NR | 20 | 36.4 | 8.9 | 20 | 42 | 3.8 | NR |
| Jouni 2017, United States | United States | Patients | multiple CVD risk factors | Physician visit satisfaction was assessed by a 6-statement survey adapted from the Consumer Assessment of Health Plans and Systems | 0-6 | 6 months | NR | 103 | 5.99 | 0.1 | 104 | 5.97 | 0.17 | 0.32 |
| Karagiannis 2016, Greece | Greece | Patients | diabetes | Satisfaction with decision made | 1 item | Immediately | NR | 103 | 35.9% (n=37) strongly agree | NR | 101 | 51.5% (n=52) strongly agree | NR | NR |
| Koelewijn-Van Loon 2010, Netherlands | Netherlands | Patients and providers | multiple CVD risk factors | 10-item satisfaction with communication | 10-50 | 12 weeks | | 258 | 35.4 | 8.6 | 264 | 37.9 | 8.4 | |

© 2024 Elias S et al. *JAMA Network Open.*

| Author, Year, Country | Country | Target | Main Topic | Measurements | Score range | Time measured | Effect size | CG: n | CG: mean | CG: SD | IG 1: n | IG : mean | IG: SD | P value |
|---|---|---|---|---|---|---|---|---|---|---|---|---|---|---|
| Krones 2008, Germany | Germany | Patients and providers | CVD risk | Patient Participation Scale | NR | Immediately | -0.80 (95% CI: -1.23 to -0.37) | 536 | 7.56 | NR | 501 | 6.76 | NR | <0.001 |
| Kulzer 2018, Germany | Germany | Patients and providers | diabetes and medication | Diabetes treatment satisfaction questionnaire | 0-48 | 12 months | OR 0.92 (95% CI 0.13 to 1.7) | 399 | 30 | 6.1 | 370 | 30.9 | 5.5 | 0.0127 |
| Kunneman 2022, United States | United States | Patients and providers | diabetes and medication | Patient satisfaction with conversation | NR | Immediately | NR | 129 | 96% (n=124) | NR | 152 | 96% (n=146) | NR | 0.98 |
| O'malley 2022, United States | United States | Patients and providers | multiple CVD risk factors and medication | 5-point Likert scale with 5 questions ranging from excellent to poor | 5-25 | Immediately | NR | 69 | 23.6 | 1.9 | 51 | 23.8 | 2.3 | 0.63 |
| Perestelo-Pérez 2016, Spain | Spain | Patients and providers | CVD risk and medication | Satisfaction with the decision-making process (SDMP)*: the 12-item questionnaire developed by Barry et al. for patients with benign prostatic hyperplasia was slightly modified to reflect a context of cardiovascular risk | 0-100 | Immediately | OR 10.62 (95% CI: 2.60 to 18.63) | 73 | 61.56 | 17.37 | 80 | 70.4 | 17.62 | 0.18 |
| van Steenkiste 2007, Netherlands | Netherlands | Patients and providers | multiple CVD risk factors | Five performance indicators: Appropriate assessment | NR | Immediately | NR | 214 | Probability 0.82 (95% CI: 0.64 to 0.92) | NR | 276 | Probability 0.85 (95% CI: 0.71 to 0.93) | NR | NR |
| Yu 2020, Canada | Canada | Patients and providers | diabetes | Patient Assessment of Chronic Illness Care | 0-5 | 12 months | 0.71 (95% CI: 0.38 to 1.04) | 79 | 3.22 | 1.08 | 72 | 3.16 | 1.1 | <0.001 |

CG: control group; CVD: cardiovascular disease; CI: confidence interval; IG: intervention group; NR: not reported; OR: odds ratio.

© 2024 Elias S et al. *JAMA Network Open.*

**eFigure 1.** Forest Plot of Hemoglobin $A_{1c}$ ($HbA_{1c}$)

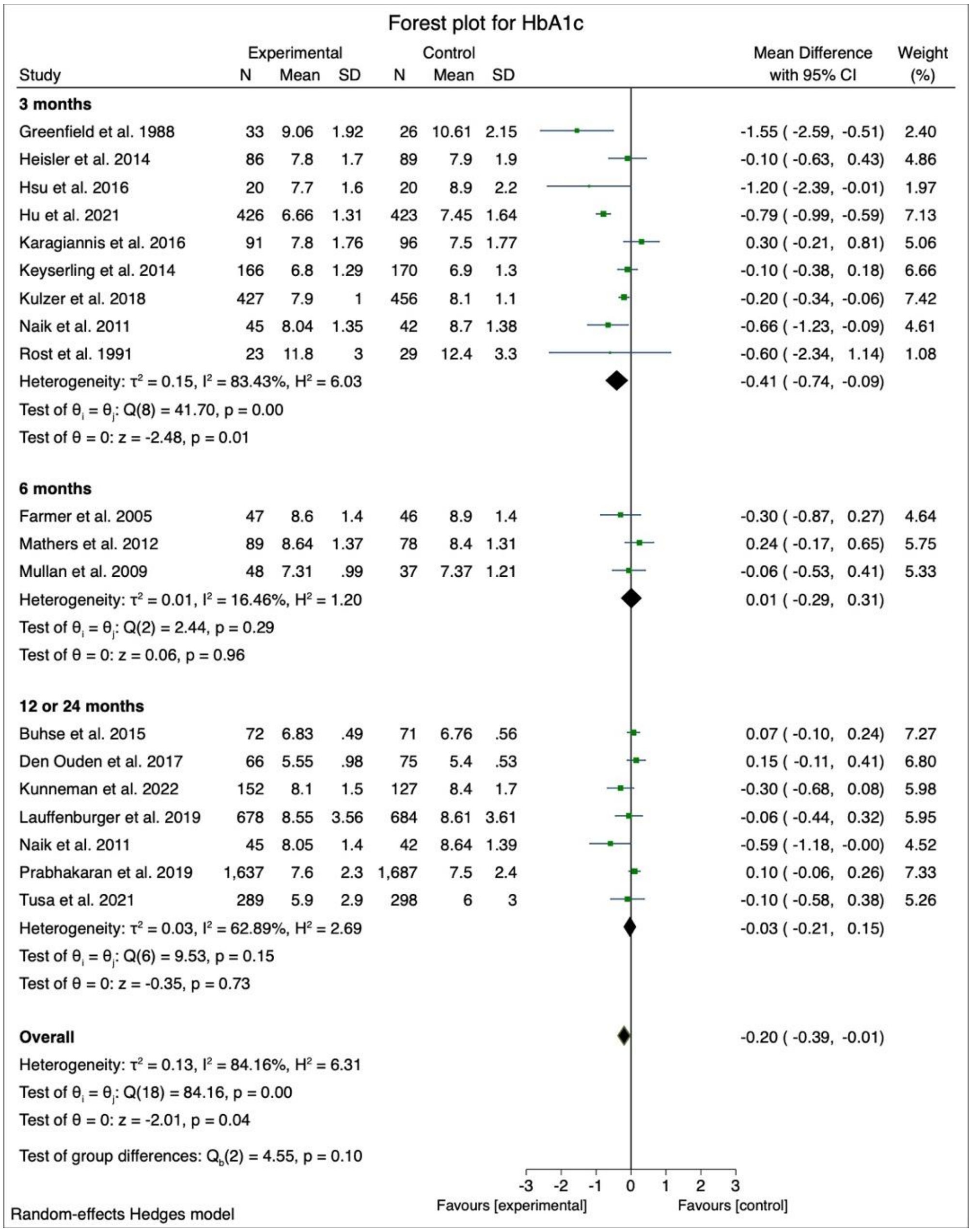


SD: Standard Deviation; CI: Confidence Interval; HbA1c: Hemoglobin A1c. The size of the squares is proportional to the weight of each study. Horizontal lines indicate the 95% CI of each study; diamond, the pooled estimate with 95% CI; and the vertical line is the line of no effect.

© 2024 Elias S et al. *JAMA Network Open.*

**eFigure 2.** Forest Plot of Systolic Blood Pressure

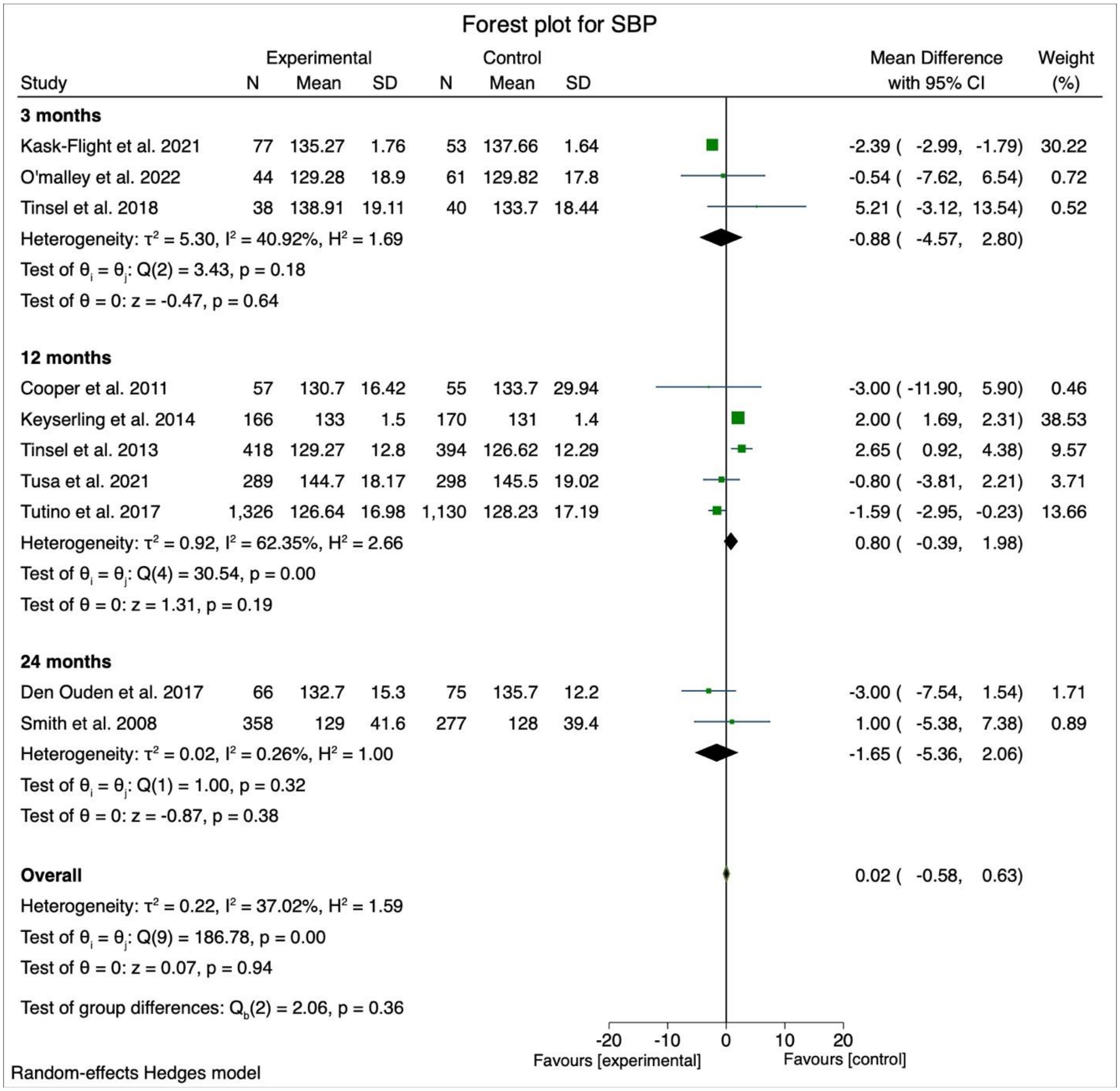


SD: Standard Deviation; CI: Confidence Interval. The size of the squares is proportional to the weight of each study. Horizontal lines indicate the 95% CI of each study; diamond, the pooled estimate with 95% CI; and the vertical line is the line of no effect.

© 2024 Elias S et al. *JAMA Network Open.*

**eFigure 3.** Funnel Plot of Decisional Conflict

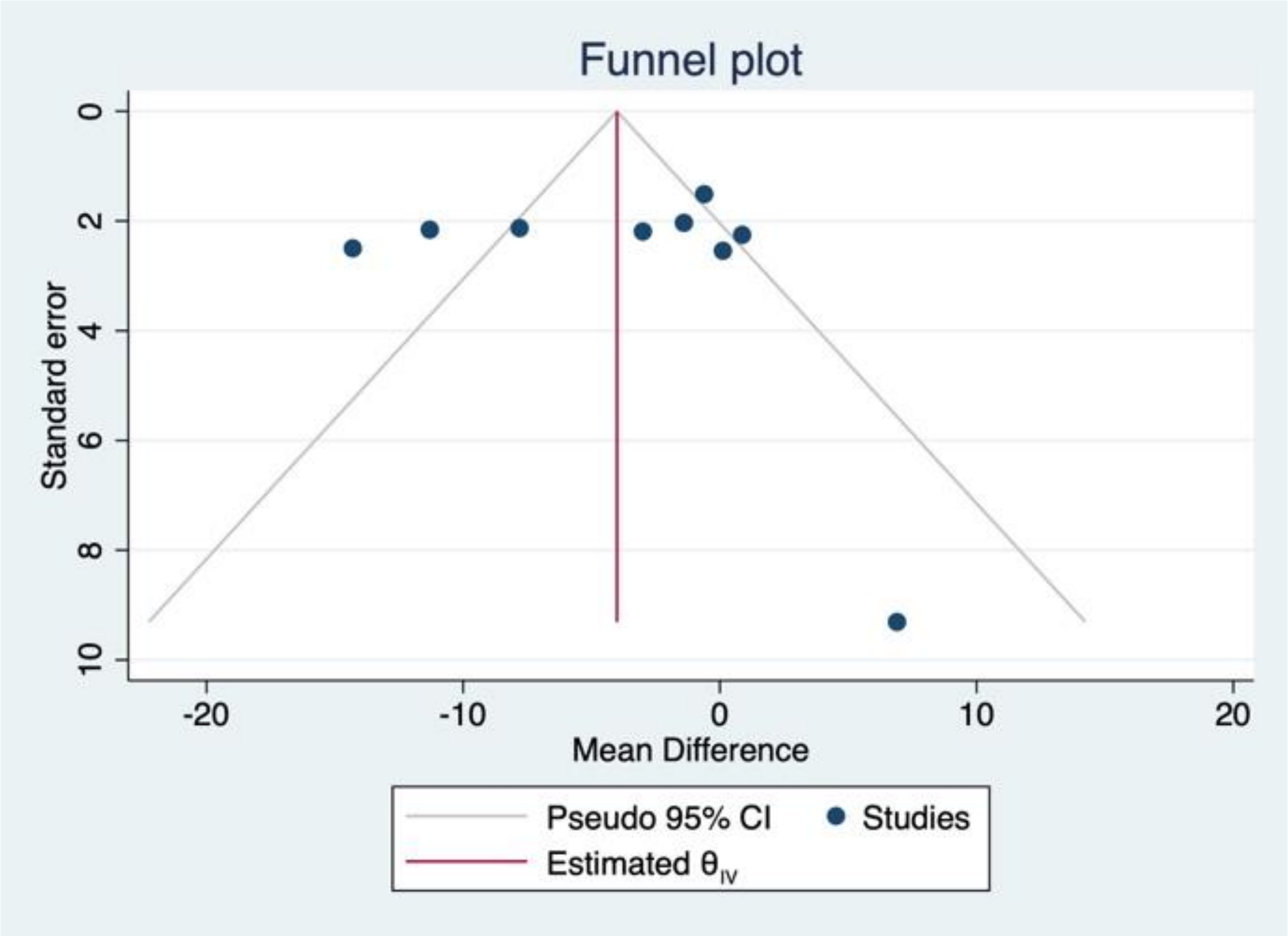


CI: Confidence Interval.

The funnel plot was generally symmetrical (Egger test, P=.890), which implied no publication bias existed in the included studies.

**eFigure 4.** Funnel Plot of Hemoglobin $A_{1c}$

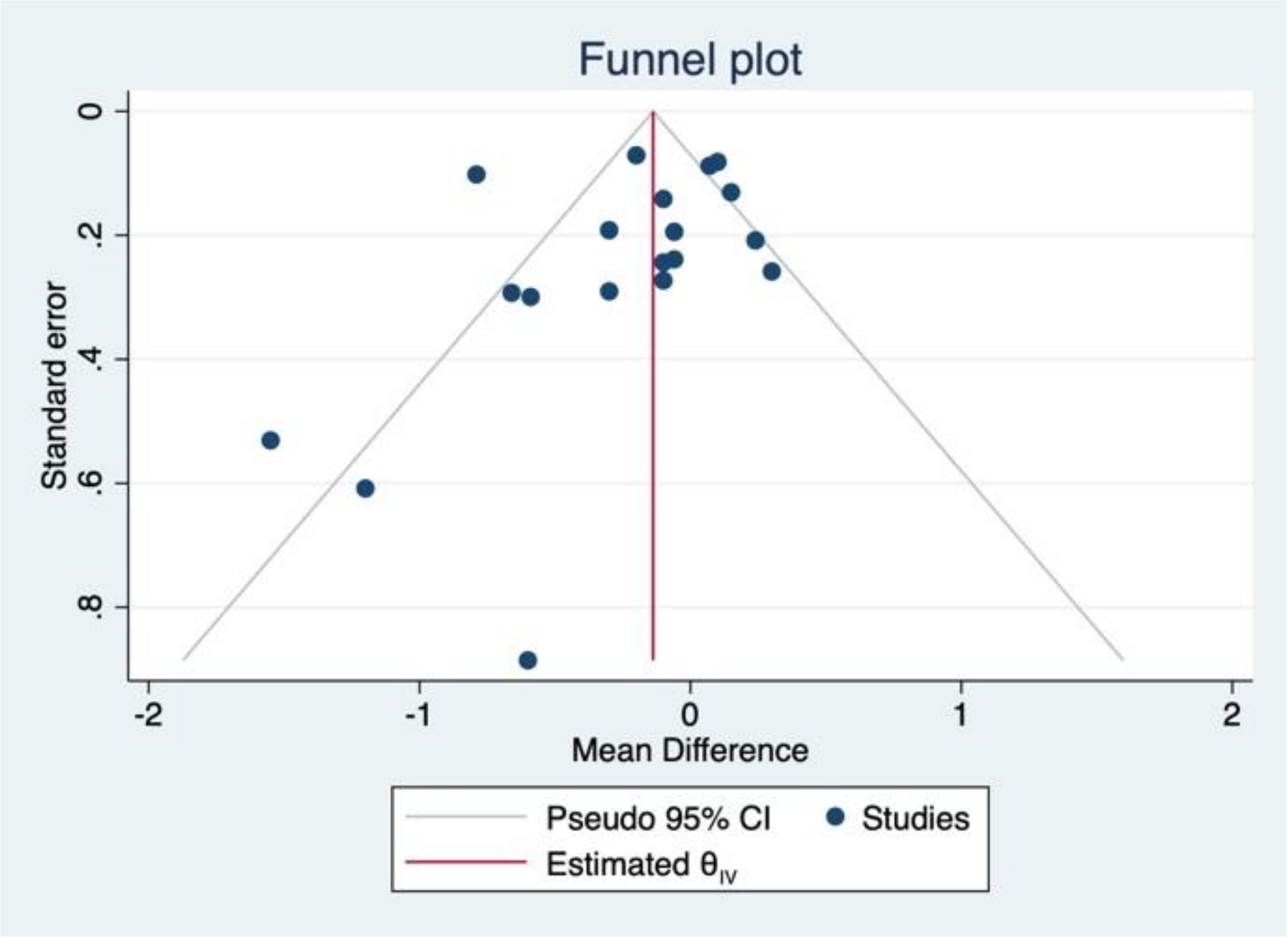


CI: Confidence Interval; HbA1c: Hemoglobin A1c

The funnel plot was generally symmetrical (Egger test, P=.376), which implied no publication bias existed in the included studies.

**eFigure 5.** Funnel Plot of Systolic Blood Pressure

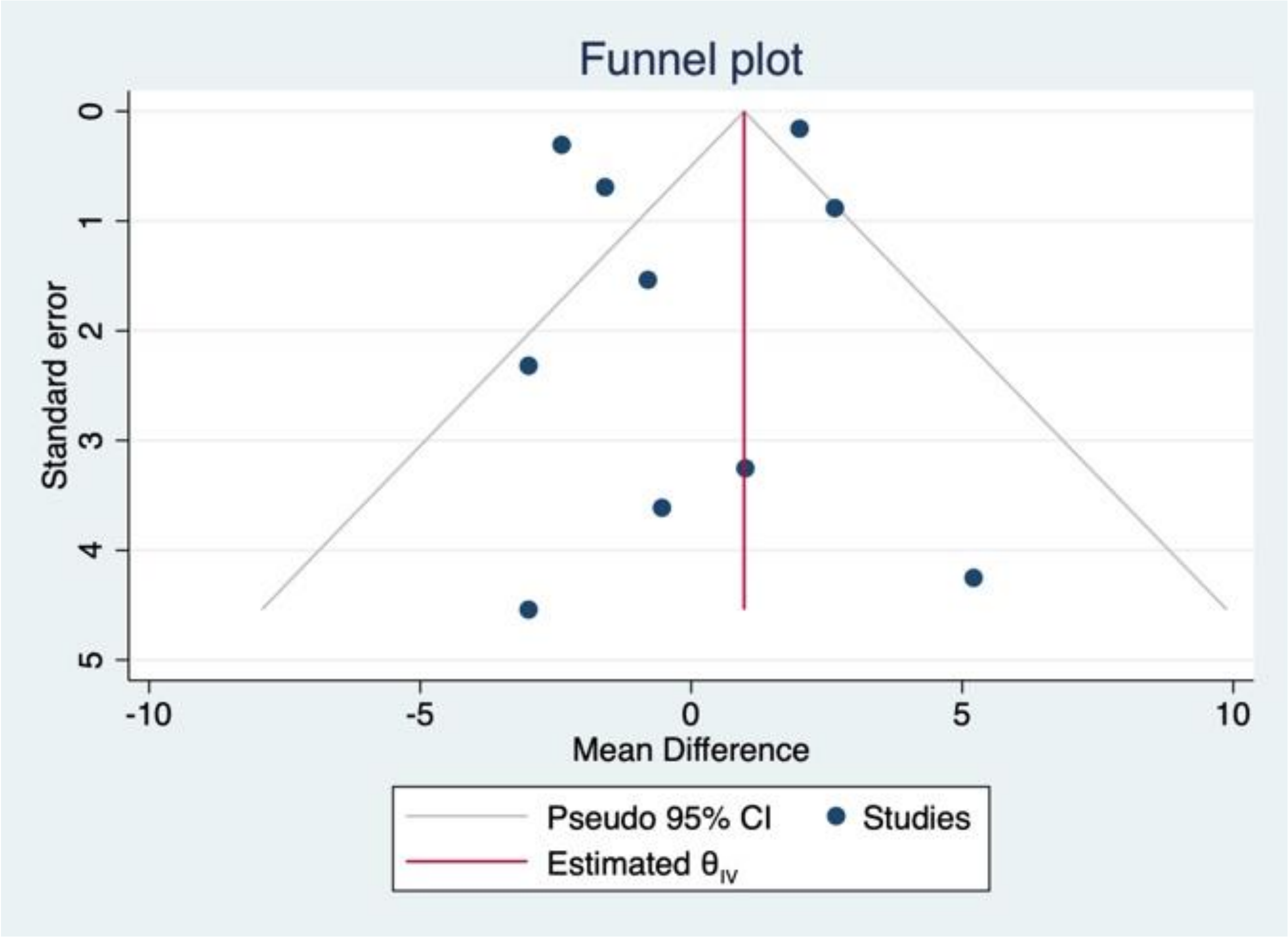


CI: Confidence Interval

The funnel plot was generally symmetrical (Egger test, P=.436), which implied no publication bias existed in the included studies.

© 2024 Elias S et al. *JAMA Network Open.*

**eFigure 6.** Forest Plot of Satisfaction About the Decision or Treatment

| Study | Experimental N | Experimental Mean | Experimental SD | Control N | Control Mean | Control SD | Standardized Mean Difference with 95% CI | Weight (%) |
|---|---|---|---|---|---|---|---|---|
| Greenfield et al. 1988 | 33 | 83.90 | 14.38 | 26 | 83.54 | 16.56 | 0.36 (−7.54, 8.27) | 7.30 |
| Heisler et al. 2014 | 92 | 89.00 | 17.50 | 95 | 88.40 | 16.00 | 0.60 (−4.20, 5.40) | 11.14 |
| Hsu et al. 2016 | 20 | 87.36 | 7.90 | 20 | 75.71 | 18.51 | 11.65 ( 2.83, 20.47) | 6.44 |
| O'malley et al. 2022 | 51 | 100.00 | 11.50 | 69 | 100.00 | 9.50 | 0.00 (−3.76, 3.76) | 12.65 |
| Perestelo−Pérez et al. 2016 | 80 | 70.40 | 17.62 | 73 | 61.56 | 17.37 | 8.84 ( 3.29, 14.39) | 10.10 |
| Heterogeneity: $\tau^2 = 19.72$, $I^2 = 70.75\%$, $H^2 = 3.42$ | | | | | | | 3.77 (−0.97, 8.52) | |
| Test of $\theta_i = \theta_j$: Q(4) = 11.70, p = 0.02 | | | | | | | | |
| Test of $\theta = 0$: z = 1.56, p = 0.12 | | | | | | | | |
| Jouni et al. 2017 | 104 | 99.52 | 2.83 | 103 | 99.85 | 1.67 | −0.33 (−0.97, 0.30) | 15.98 |
| Koelewijn−Van Loon et al. 2010 | 264 | 94.75 | 21.00 | 258 | 88.50 | 21.50 | 6.25 ( 2.60, 9.90) | 12.82 |
| Kulzer et al. 2018 | 370 | 64.27 | 11.44 | 399 | 62.40 | 12.69 | 1.87 ( 0.16, 3.58) | 15.25 |
| Yu et al. 2020 | 72 | 63.20 | 22.00 | 79 | 64.40 | 21.60 | −1.20 (−8.16, 5.76) | 8.32 |
| Heterogeneity: $\tau^2 = 6.85$, $I^2 = 87.06\%$, $H^2 = 7.73$ | | | | | | | 1.75 (−1.26, 4.77) | |
| Test of $\theta_i = \theta_j$: Q(3) = 16.97, p = 0.00 | | | | | | | | |
| Test of $\theta = 0$: z = 1.14, p = 0.25 | | | | | | | | |
| | | | | | | | 2.67 (−0.22, 5.56) | |
| Heterogeneity: $\tau^2 = 13.48$, $I^2 = 87.29\%$, $H^2 = 7.87$ | | | | | | | | |
| Test of $\theta_i = \theta_j$: Q(8) = 32.91, p = 0.00 | | | | | | | | |
| Test of $\theta = 0$: z = 1.81, p = 0.07 | | | | | | | | |
| Test of group differences: $Q_b(1) = 0.50$, p = 0.48 | | | | | | | | |

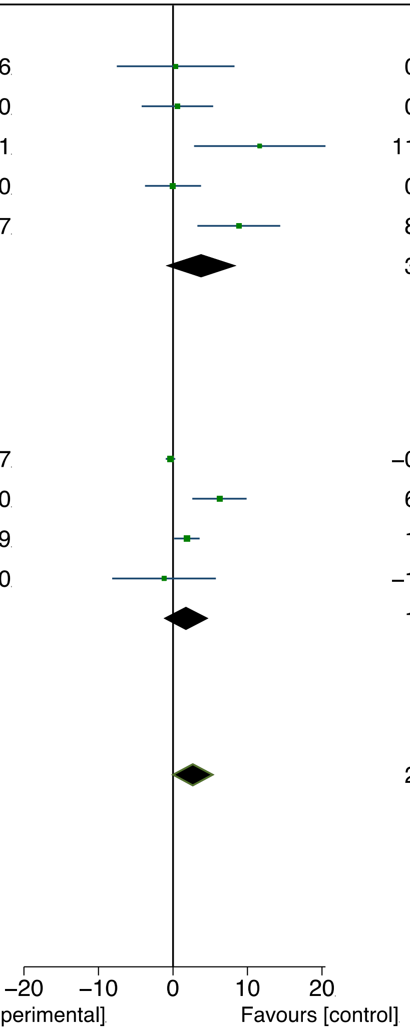


Random−effects Hedges model

SD: Standard Deviation; CI: Confidence Interval. Data were pooled using standardized mean difference, and their corresponding 95% CIs were computed after standardizing to a scale from 0 to 100, where a higher score indicated a better outcome. The size of the squares is proportional to the weight of each study. Horizontal lines indicate the 95% CI of each study; diamond, the pooled estimate with 95% CI; and the vertical line is the line of no effect.

© 2024 Elias S et al. *JAMA Network Open.*

**eFigure 7.** Funnel Plot of Satisfaction About the Decision or Treatment

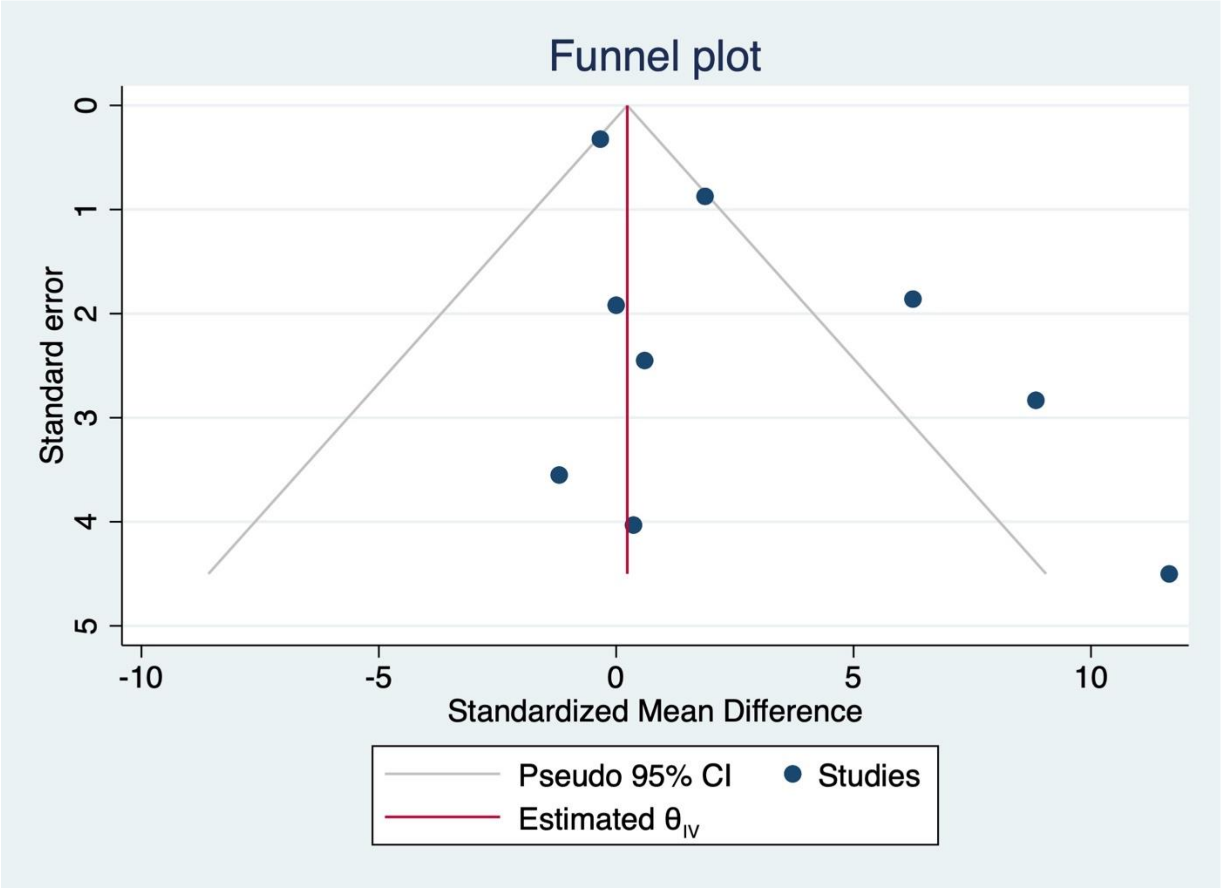


CI: Confidence Interval

The funnel plot was not generally symmetrical (Egger test, P=.052), which implied potential publication bias existed in the included studies.

© 2024 Elias S et al. *JAMA Network Open.*

## eReferences


1. Assessing Cardiovascular Risk: Systematic Evidence Review from the Risk Assessment Work Group. Nov 2013. https://www.nhlbi.nih.gov/health-topics/assessing-cardiovascular-risk.
2. Uffelman CN, Chan NI, Davis EM, Wang Y, McGowan BS, Campbell WW. An Assessment of Mushroom Consumption on Cardiometabolic Disease Risk Factors and Morbidities in Humans: A Systematic Review. *Nutrients*. 2023;15(5). doi:10.3390/NU15051079
3. Dettori JR, Norvell DC, Chapman JR. Fixed-Effect vs Random-Effects Models for Meta-Analysis: 3 Points to Consider. *Global Spine J*. 2022;12(7):1624-1626. doi:10.1177/21925682221110527/ASSET/IMAGES/LARGE/10.1177_21925682221110527-FIG3.JPEG
4. Higgins JPT TJCJCMLTPMWV (editors). Cochrane Handbook for Systematic Reviews of Interventions version 6.4 (updated August 2023). Cochrane, 2023. Available from www.training.cochrane.org/handbook.
5. Sterne JAC, Egger M. Funnel plots for detecting bias in meta-analysis: Guidelines on choice of axis. *J Clin Epidemiol*. 2001;54(10):1046-1055. doi:10.1016/S0895-4356(01)00377-8


© 2024 Elias S et al. *JAMA Network Open.*
