# Supplementary material for: Shared Decision-Making in Cardiovascular Risk Factor Management: A Systematic Review and Meta-Analysis
Source: JAMA Netw Open. 2024 Mar 26;7(3):e243779. doi: 10.1001/jamanetworkopen.2024.3779 (PMC10966415; doi:10.1001/jamanetworkopen.2024.3779)
Supplement: Supplement 2. — Data Sharing Statement [file jamanetwopen-e243779-s002.pdf]

Data Sharing Statement

Elias. Shared Decision-Making in Cardiovascular Risk Factor Management. *JAMA Netw Open*. Published March 26, 2024. doi:10.1001/jamanetworkopen.2024.3779

Data
Data available: No

Additional Information
Explanation for why data not available: For purposes of reproducing the results, the data, analytical methods, and study materials are available from the corresponding author upon reasonable request.
